# A novel approach to sequence validating protein expression clones with automated decision making

**DOI:** 10.1186/1471-2105-8-198

**Published:** 2007-06-13

**Authors:** Elena Taycher, Andreas Rolfs, Yanhui Hu, Dongmei Zuo, Stephanie E Mohr, Janice Williamson, Joshua LaBaer

**Affiliations:** 1 Harvard Institute of Proteomics, Harvard Medical School, 320 Charles St., Cambridge, MA 02141, USA; 2 DF/HCC DNA Resource Core, Harvard Medical School, 320 Charles St., Cambridge, MA 02141, USA

## Abstract

**Background:**

Whereas the molecular assembly of protein expression clones is readily automated and routinely accomplished in high throughput, sequence verification of these clones is still largely performed manually, an arduous and time consuming process. The ultimate goal of validation is to determine if a given plasmid clone matches its reference sequence sufficiently to be "acceptable" for use in protein expression experiments. Given the accelerating increase in availability of tens of thousands of unverified clones, there is a strong demand for rapid, efficient and accurate software that automates clone validation.

**Results:**

We have developed an Automated Clone Evaluation (ACE) system – the first comprehensive, multi-platform, web-based plasmid sequence verification software package. ACE automates the clone verification process by defining each clone sequence as a list of multidimensional discrepancy objects, each describing a difference between the clone and its expected sequence including the resulting polypeptide consequences. To evaluate clones automatically, this list can be compared against user acceptance criteria that specify the allowable number of discrepancies of each type. This strategy allows users to re-evaluate the same set of clones against different acceptance criteria as needed for use in other experiments. ACE manages the entire sequence validation process including contig management, identifying and annotating discrepancies, determining if discrepancies correspond to polymorphisms and clone finishing. Designed to manage thousands of clones simultaneously, ACE maintains a relational database to store information about clones at various completion stages, project processing parameters and acceptance criteria. In a direct comparison, the automated analysis by ACE took less time and was more accurate than a manual analysis of a 93 gene clone set.

**Conclusion:**

ACE was designed to facilitate high throughput clone sequence verification projects. The software has been used successfully to evaluate more than 55,000 clones at the Harvard Institute of Proteomics. The software dramatically reduced the amount of time and labor required to evaluate clone sequences and decreased the number of missed sequence discrepancies, which commonly occur during manual evaluation. In addition, ACE helped to reduce the number of sequencing reads needed to achieve adequate coverage for making decisions on clones.

## Background

The impact of the genome sequencing projects will emerge from elucidating protein function. Diseases result from protein dysfunction and are managed with drugs that alter protein function. Although protein function has been inferred from sequence similarities, it has not been directly studied in most cases. There is a substantial need to develop high throughput (HT) tools that will accelerate the study of the thousands of proteins not yet examined, a field referred to as *functional proteomics*.

Protein function studies start by producing proteins using cloned copies of the genes that encode them. Recognition of this has led to the production of large collections of cloned genes configured in a protein expression-ready format (ORF collections). To ensure accurate conclusions, the coding sequences must first be validated, a process that includes comparing the clones' sequences to the expected sequences at the nucleotide and amino acid levels. Yet, despite the well-recognized importance of sequence verifying cloned genes, it has been performed on less than a handful of protein-expression clone collections currently in existence [1-6].

Assembling clones is now well-established, relatively inexpensive and automated. However, several key steps – oligonucleotide synthesis, reverse transcription, and PCR – are unavoidably mutagenic, emphasizing the importance of sequence verification. The ultimate goal of validation is to determine if a given clone is "acceptable" for use in biological experiments. Acceptance criteria may vary based on the experiment but typically a clone is rejected when its coding sequence contains one or more mutations that might adversely affect protein activity.

In contrast to building the clones, the process of verifying their sequences is still handled manually. Whereas excellent software has been written to manage and automate the *de novo *sequencing of DNA, and although elements of that software can be employed (such as sequence alignment, contig assembly, primer design, etc.), there is no software that manages the process of validating clone sequences, particularly for large projects. There are a number of attributes unique to the clone validation process that distinguish it from *denovo *sequencing and require the development of novel software. First, whereas the goal of *de novo *sequencing is to make the best sequence prediction for an unknown, the goal of validation is to determine if an unknown matches *a pre-defined reference sequence*. Second, clone validation software should provide an automated mechanism for sorting clones into either the accepted or rejected categories. Third, the evaluation process must also consider the polypeptide sequence because, in general, the amino acid sequence dominates in determining the clone's overall value. Fourth, in genomic sequencing, discrepancies among reads arise primarily due to sequencing errors, whereas with clone validation, discrepancies arise not only from sequencing errors but also because of polymorphisms in the source material, PCR errors, oligonucleotide synthesis errors, reverse transcription errors and even mistakes or ambiguity in the reference sequence. Finally, the availability of a reference sequence affects the strategies that software would employ in managing the projects, for example, favoring primer walk strategies over transposon and shotgun alternatives for full length sequencing.

Software for clone validation should thus be able to: 1) determine the sequence of each clone accurately; 2) identify if and where that sequence varies from the intended target sequence; 3) evaluate and annotate the polypeptide consequences of any variations; and 4) determine if these observed differences are acceptable based on user defined criteria.

Thus far, software developed to aid in DNA sequencing has focused on the *de novo *sequence determination of DNA fragments, such as in genomic sequencing projects. Some basic sequencing programs handle pre-processing of sequence trace files, base calling [7], quality clipping, vector trimming and removal [8], contig assembly (phrap [8] or TIGR assembler [9]) and primer design [10]. These programs are often used as integral parts of sophisticated software packages (Staden [11], Gasp [12], Lucy [13,14]) which include additional functionality like clustering, gene annotation and finishing tools (Autofinish [15] from the Staden package). Several packages have been developed to manage expressed sequence tag (EST) projects (ESTIMA [16], ESTAP [17], ESTWeb [18]). Recently comprehensive modular software packages have been developed that provide a graphical user interface (GUI) for biologists with minimum software experience to define their own processing pipelines for sequencing, managing sequencing results and performing basic analyses like sequence alignment and computing quality statistics (MAGIC_CP [19], Pegasys [20]). These packages typically rely upon the basic bioinformatics programs mentioned above to perform unit tasks and relational databases as backend storage for processed data.

Software tools used in "re-sequencing" projects (such as sequencing the same gene in many individuals to find polymorphisms) bear some similarity to the concept of clone validation (e.g., PolyBayes [21], PolyPhred [22], noSnp [23], SNPdetector [24]). However none of these programs consider polypeptide consequences of differences nor do they provide a mechanism for applying acceptance criteria within the workflow. Moreover, SNP discovery software has been designed to operate with dense sequence coverage of specific target regions whereas clone validation software must deal with the minimum possible coverage achieved for many different genes (clones).

In this paper, we present the Automated Clone Evaluation (ACE) system, which is an automated software application that simultaneously manages the process of sequence validation for thousands of plasmid clones. ACE has been used successfully to evaluate more than 55,000 clones at the Harvard Institute of Proteomics, providing full automation for all processes and assisting in building sequence verified clone collections useful for HT proteomic studies.

## Implementation

### Conceptual approach

The two central requirements for automated clone validation software are the abilities: (1) to identify discrepancies between the clones' determined sequence and the expected sequence at the nucleotide and polypeptide level; and (2) to automatically sort clones into acceptability categories (i.e., acceptable, reject, needs further review) based on user defined criteria. A discrepancy is any difference between a clone and its expected sequence and may arise because of cloning artifacts, mistakes in determining the clone's sequence, natural occurring polymorphisms or errors in the reference sequence. Moreover, discrepancies will have varying effects on the predicted polypeptide, from silent (no amino acid) changes to truncations. Both the causes and consequences of discrepancies are important to end users. Thus, for the software to make informed decisions regarding the acceptability of clones, it must also collect and use this ancillary information.

The strategy employed by ACE is to describe each clone as a list of multidimensional discrepancy objects. ACE populates multiple properties of discrepancy objects including: discrepancy type (see Table 1), sequence confidence level (low or high), and position on the reference sequence. Additionally, discrepancy objects fully describe the predicted consequences on both the nucleotide and polypeptide levels (e.g. sequences locations, inserted/substituted bases and amino acids, number of deleted bases and amino acids).

**Table 1:** Definitions of Discrepancy Types.

***Discrepancies defined on nucleotide and polypeptide levels***	
Nucleotide changes	Polypeptide changes

Substitution	Silent substitution
	Conservative substitution
	Non-Conservative substitution
	Missense substitution – any amino acid change
	Truncation – inframe Stop codon
Frameshift deletion	Frameshift deletion
Frameshift insertion	Frameshift insertion
Inframe deletion	Inframe deletion
Inframe insertion	Inframe insertion
No Stop codon	Post-elongation
No Start codon	No Translation

***Discrepancies defined on nucleotide level only***	

**Discrepancies introduced by sequence ambiguity**	

Start codon substitution	
Stop codon substitution	
Substitution in CDS region	
Frameshift insertion	
Inframe insertion	
**Discrepancies introduced by reference sequence ambiguity**	
Substitution in CDS region	
**Flanking sequence region (5' and 3' regions described separately)**	
Substitution – replacement of one base by another	
Deletion/insertion – deletion/insertion of several bases	
Ambiguous substitution – replacement of a base by ambiguous base	
Ambiguous insertion – insertion of ambiguous base	

Discrepancy types for cDNA and flanking sequences defined at the nucleotide and polypeptide level by the Discrepancy Finder.	

To automatically sort the clones, ACE compares the list of discrepancy objects for each clone against the user's acceptance/rejection criteria for discrepancies of various types. The existence of any discrepancy is always deleterious; but users consider some more deleterious than others. For example, some users require an exact match to their expected sequence (no discrepancies) whereas others would allow one amino acid change and still others might also allow that change only if it represents a natural polymorphism. It is also important to consider the base confidence of discrepancy objects because the most common cause of low confidence discrepancies is base calling errors (i.e., the clone is correct, but its predicted sequence is wrong).

The strategy of describing clones as lists of discrepancy objects separates the clone *annotation *process from the clone *sorting *process. Annotation defines the discrepancies, which are inherent and objective features of the clones. The sorting process compares lists of discrepancies according to a subjective value system defined by the user. Thus, the same set of clones can be sorted based on different user criteria for different applications. Finding the "best" among several clones for the same gene is reduced to comparing their discrepancy lists. Moreover, it allows these comparisons to be made at the level of polypeptide consequences, which is most relevant for the functional proteomics purposes.

### Application overview

ACE is a comprehensive, multi-platform and multi-user, web-based sequence verification software system. The key novel aspects of ACE include: (1) finding and annotating discrepancies between clone and reference sequences, including clones with incomplete sequence assemblies; (2) describing each clone sequence as a list of multidimensional discrepancy objects; (3) implementing an automated decision making process that compares each clone's discrepancy object list to user-defined clone acceptance criteria; (4) embedding in each discrepancy object information about the polypeptide consequences of the discrepancy; (5) automating selection of the best isolate when multiple isolates for a target sequence are available.

ACE provides an integrated environment that relieves the user from routine process management tasks, such as bookkeeping of all project- and clone-related information, re-entering of parameters and criteria, and history tracking. It automates all steps of sequence verification, including primer design, sequence contig assembly, gap mapping, demarcating low confidence regions in sequence coverage and identifying polymorphisms.

Whenever possible, ACE implements existing well-established algorithms and utilizes third-party programs via custom wrappers that adapt them to the specific tasks. For example, we modified a Phred/Phrap [7,8] script to allow users to remove low-quality reads from an assembly and/or to trim tails of the reads according to user defined criteria. NCBI BLAST [25,26] and *needle *[27] are used, respectively, for local and global sequence alignments. Primer3 [10] is launched iteratively using the clone's reference sequence as a guide to enable primer design for automated primer walking.

**Figure 1:**
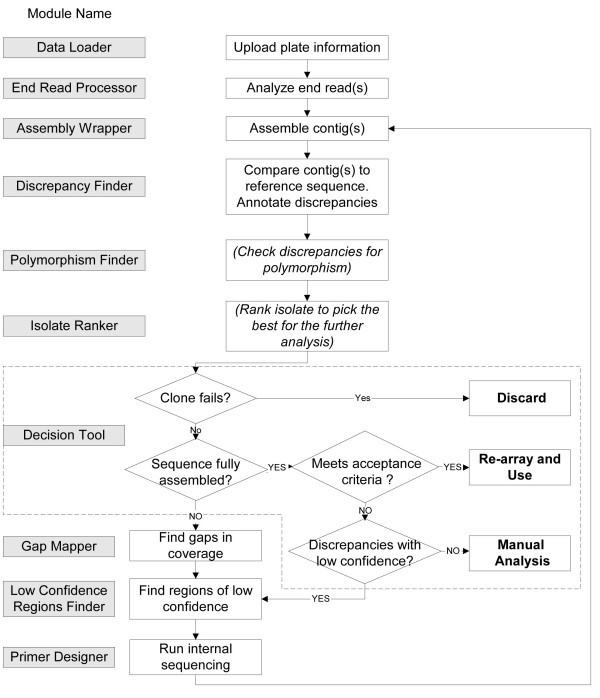
**Block diagram of typical clone sequence verification workflow**. The diagram illustrates a typical workflow for the full length sequence validation of a protein coding clone. The process, which is described in detail in the text, is an iterative process that collects sequence reads, assembles contigs, finds gaps in coverage, finds regions of low confidence, compares the contig sequence with the expected sequence, and determines the overall acceptability of the clone. Processing steps in parentheses and italics are optional.

**Figure 2:**
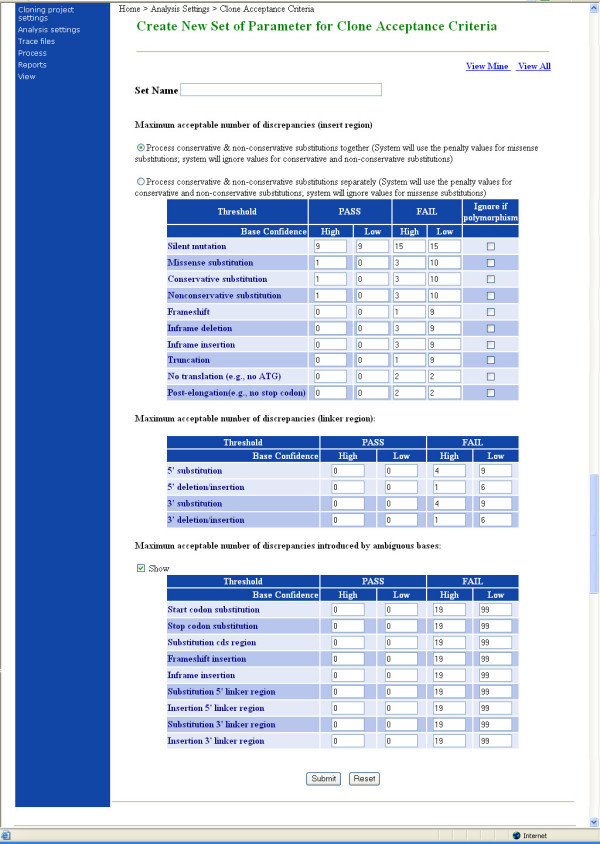
**Clone Acceptance Criteria**. This screenshot shows the interface to establish acceptance criteria by setting the maximum allowed number of discrepancies of each type. Different values can be set for discrepancies of low and high confidence. The user sets values for two thresholds – one that triggers a manual review, and one that automatically rejects the clone. Users can also opt to handle conservative and non-conservative amino acids substitutions separately or to treat all amino acid changes as one type. Once the settings are created, users can name the set and store it for future use. In this way, users may create different acceptance criteria for different purposes. Thus, a single collection of clones can be evaluated by different acceptance criteria by invoking these named sets. The criteria shown here are used routinely for determining final acceptance of clones. The numbers in the boxes indicate the absolute number of the indicated type of discrepancy for inclusion in that category. As indicated, this set of criteria does not distinguish between conservative and non-conservative missense mutations. Any clones with 1 or 0 high confidence missense substitution(s) are automatically accepted (as long as they have no other discrepancies that prevent automatic acceptance). Clones with 3 or more high-confidence missense substitutions are automatically rejected; if the clones have 2 they are triaged for additional sequencing or manual analysis. A higher bar is set to automatically reject clones based on low-confidence substitutions (10 or more), because many of these will be resolved with further sequencing. Similarly, this parameter set automatically passes clones only if they have no frameshift discrepancies of any type. Clones with 1 high-confidence or 9 low-confidence frameshift discrepancies or more are automatically rejected. Clones must meet all the pass criteria for automatic acceptance, whereas clones that meet any automatic fail criteria are automatically failed.

ACE is structured for maximum flexibility to support different approaches to clone validation and sequencing management. Users do not need to follow a single path in clone sequence verification, but rather can invoke each module individually. A typical ACE-based workflow used in our laboratory is shown in Figure 1. A project begins with end read sequencing of one or more clonal isolates per target. End reads are acquired, assigned to their corresponding clone (End Read Processor), and then processed by the assembler to determine if end reads alone are sufficient to yield a complete contig assembly (Assembly Wrapper). Whether or not the assembly yielded a single contig covering the full-length CDS, clone contig(s) are analyzed to detect differences or "discrepancies" compared with the reference/target sequence (Discrepancy Finder). ACE can compare any discrepancies with one or more sequence database, such as GenBank, to determine if they correspond to naturally occurring polymorphisms (Polymorphism Finder). During the final decision process, users can optionally configure the software to avoid penalizing clones for discrepancies that represent polymorphisms (Figure 2). If more than one isolate exists for a given clone, an optional module (Isolate Ranker) can rank isolates based on user-defined preferences specified in the form of penalties associated with different types of discrepancies.

Clones that failed to assemble into a single contig covering the CDS can be scanned to find the remaining gaps in sequence coverage (Gap Mapper). In addition, sequence regions of low confidence can be analyzed to demarcate their boundaries (Low Confidence Regions Finder). Subsequently, clones with low confidence regions or gaps in sequence coverage can be processed to define appropriate internal sequencing primers to cover those regions (Primer Designer). At any stage during the clone verification process, a set of clones can be processed by the Decision Tool in order to determine how far each clone has progressed in the analysis pipeline and its acceptance/rejection status.

Representative output files and user interfaces for each of the key modules in this application can be found in Additional files [see Additional files 1 and 2].

### Software Architecture

In its implementation ACE comprises Core Classes, Wrapper Classes and Processing Modules. Core Classes represent subject domain objects, such as *clone *or *plate*, as well as process control objects, such as *project *or *analysis spec *(set of parameters used for analyzing sequence). A relational database is used as a repository of all persistent data except for trace files. Trace files are stored on a server hard drive (see Trace File Distributor module for more details).

Core Classes serve as a layer of abstraction to this database by encapsulating SQL queries for retrieving their data and for updating the database as processing proceeds.

Wrapper Classes encapsulate third party programs and are responsible for adjusting their parameters and parsing results into objects that can be processed by Processing Modules.

Processing Modules contain user interfaces and encapsulate processing algorithms. They are responsible for interpreting user requests, creation, modification or deletion of Core Objects and providing feedback to the user. To prevent accidental loss or corruption of data, several levels of access rights are defined in ACE and some functionality is available only to the users with higher level of privileges. Typically, processing modules operate on user-specified clones or collections of clones. Although ACE does not impose a strict workflow on the user, some restrictions on the order of operations still apply. Most processing modules check whether necessary conditions are met for a selected group of clones. For example, neither Decision Tool nor Isolate Ranker can process the clones if their available sequence coverage was not analyzed first by Discrepancy Finder.

The first group of processing modules is responsible for data entry and direct data management by the user. The creation of a new project requires entering the project description and defining the cloning strategy, which includes a description of the cloning vector, linkers, and the intended start and/or stop codons. Multiple projects can refer to the same cloning strategy. Data load modules are responsible for loading: (a) clone related information using XML files [see Additional files 3, 4, 5]; (b) trace files (see detailed description of Trace File Distributor and End Read Processor below); (c) clone sequences that may be provided in FASTA format.

The second group of modules is responsible for the analysis of clone data, annotating the clone, assisting the user with any required additional sequencing and finishing the clone if necessary. These modules constitute the core of ACE and are described in more detail in the next section.

**Figure 3:**
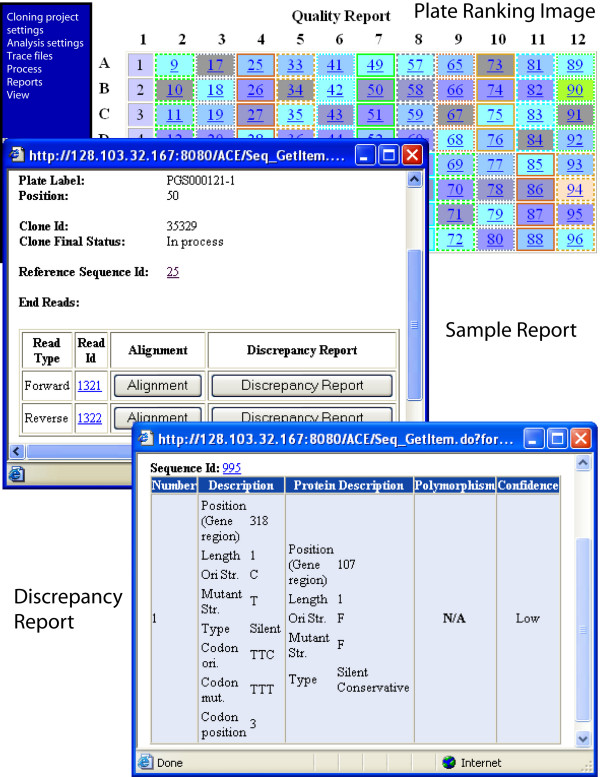
**Isolate ranking and sample reports**. A screenshot of ACE showing the plate presentation produced by the Isolate Ranker showing a color-coded clone rank; a Sample Report that provides information about the available end reads, contig collections and fully assembled sequences for the clone; and a Discrepancy Report that provides details about each discrepancy for the contig.

The third group of modules generates ACE views and reports. ACE provides user feedback in the form of interactive views, reports and email notifications. For processes that involve manageable screen content or that require real-time management, interactive views give user-friendly access to currently available information. These include: Plate Viewer (Figure 3 and Additional file 1, Figure 1) that gives accesses to complete information for specific plates and the Designed Primers viewer to select which automatically generated primers to use [see Additional file 1, Figure 2], etc. For processes involving thousands of clones or whenever a documented result is requested, each analysis tool or report can be launched in an asynchronous way, so that the user is notified at the end of the operation by email with attached report. Some reports represent direct database queries, whereas others, like the Decision Tool, Trace File Quality Assessment, and Mismatch Report include complex data processing.

**Figure 4:**
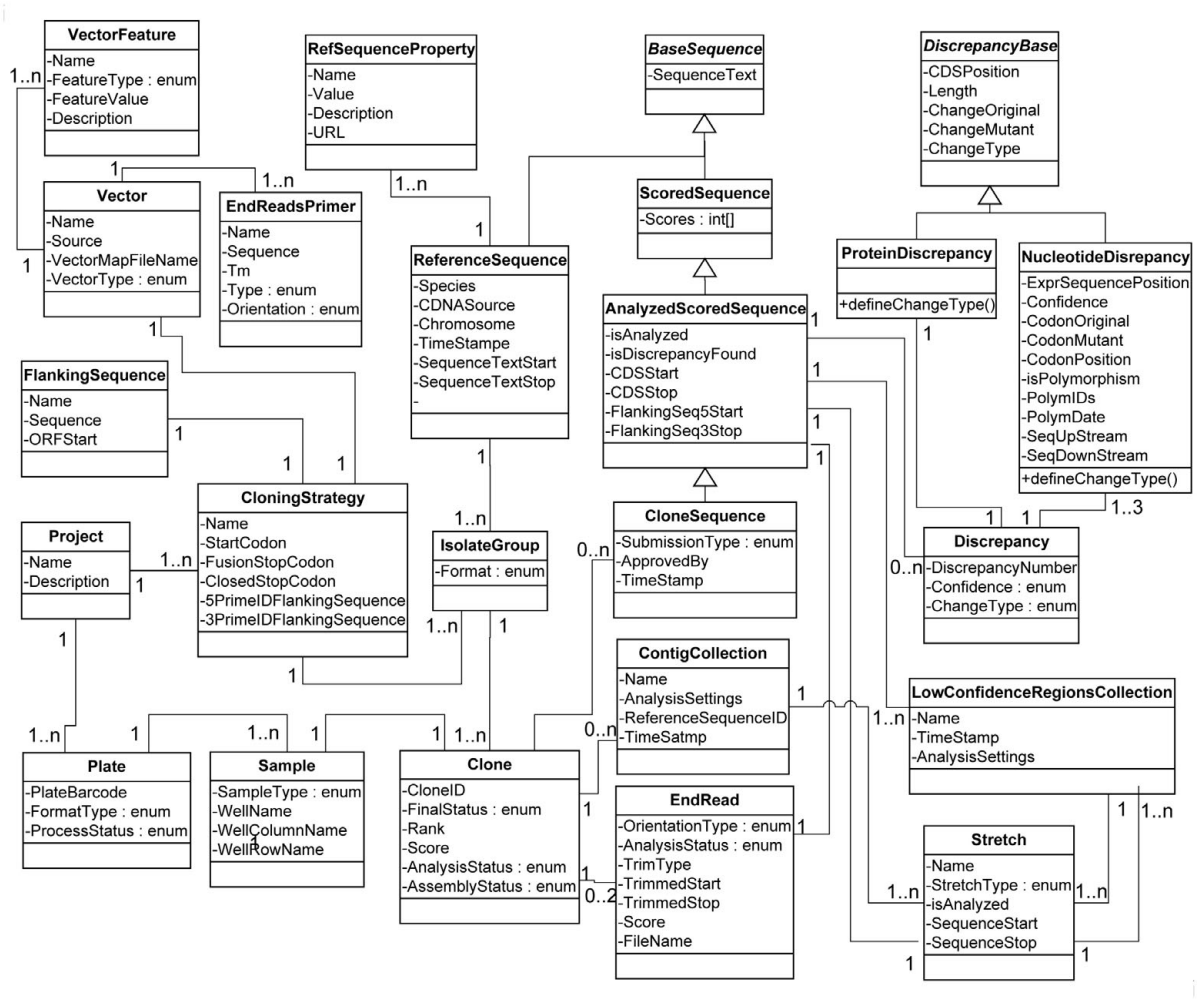
**Overview of conceptual structure and relations between most important Core Classes**. This diagram represents the Core Classes that describe clone-related information. Classes supporting process control and primer design are omitted from the figure for simplicity.

A detailed structure and the relationships among the most important Core Classes are presented in Figure 4. For simplicity, classes that support process control and primer design are omitted from the figure.

In ACE we define a class hierarchy for the description of cDNA sequences. The *BaseSequence *class is a nucleotide string. *RefSequence *is a description of the target sequence to be verified; it contains a set of literature identifiers associated with the target sequence, such as GI, GenBank Accession Number, Gene Name. Reference sequences frequently represent GenBank records, where the record sequence is longer than the clone's insert sequence. Consequently, *RefSequence *in ACE also contains the coordinates of the target sequence on the nucleotide sequence. The *ScoredSequence *class is derived from *BaseSequence *by adding the confidence score (as defined by Phred/Phrap [7,8]) to each nucleotide. *AnalysedSequence *is derived from the *ScoredSequence *and adds the collection of *DiscrepancyDescription *objects, the collection of low confidence regions (LCR) as well as the mapping of *AnalysedSequence *on the range of the target sequence. *AnalysedSequence *is the parent class for *CloneSequence *that represents full clone coverage and specifies how the sequence was obtained.

A discrepancy in ACE refers to any mismatch between *ScoredSequence *and corresponding target sequence. The *Discrepancy *class aggregates nucleotide and polypeptide level descriptions of the mismatch. In general, *Discrepancy *objects that describe nucleotide substitutions correspond to one codon and, hence, can contain up to three *NucleotideDiscrepancy *objects and one *ProteinDiscrepancy *object. Table 1 describes the correspondence between nucleotide and protein discrepancies. In some cases (e.g. for non-coding flanking sequences or for ambiguities in the sequence), *ProteinDiscrepancy *is absent and *Discrepancy *consists of a single *NucleotideDiscrepancy*. *Discrepancy *objects are created by the Discrepancy Finder and are discussed in more detail in the corresponding section.

The *Clone *object aggregates all information about the insert subject to validation. Data in the *Clone *object are accumulated in the course of processing. The class *Clone *contains: (a) physical location data (plate and sample information); (b) reference sequence information (*RefSequence *object); (c) one or two *EndRead *objects; (d) contig collection(s); (e) clone sequence(s).

*Plate *objects represent physical plates of predefined format (96- or 384-well).*Plate *contains multiple *Sample *objects, where a sample is a physical substance sitting in a particular well. The *Sample *can be a control or a clone. It is important to underscore that a single *Clone *object corresponds to a single *Sample*. If the workflow involves creation of several candidate isolates for the same target gene, each isolate is represented as a separate *Clone *object. The *IsolateGroup *object corresponds to a set of isolates with a common target.

### Core Modules

#### 1. Clone Analysis

During a large scale sequence validation project, the analysis of clones is an ongoing and iterative process. Each cycle includes acceptance of good clones, the elimination of failed clones and identification of gaps in the remaining, potentially acceptable clones.

##### 1.1 Discrepancy Finder

This module detects mismatches between the clone contig(s) and reference sequence, creating a list of *Discrepancy *objects for each clone.

Discrepancy Finder first builds a global alignment between the clone's contig(s) and its reference nucleotide sequence using the Needleman Wunsch algorithm [28](needle program from EMBOSS package [27]). Mismatches are identified via base-by-base comparison and a *NucleotideDiscrepancy *object is created for each, except that contiguous mismatches (e.g., a multiple base-pair deletion in one region) are grouped together to form a single *NucleotideDiscrepancy *object. When up to three adjacent *NucleotideDiscrepancies *belong to one codon inside the ORF sequence and none of them is an ambiguous substitution, we create a corresponding *ProteinDiscrepancy *object (see Figure 4).

ACE assigns low-confidence status to a discrepancy if the Phred confidence score [29,30] of at least one base used to define the discrepancy or one out of four bases on either side of the discrepancy is below the user-defined threshold. All discrepancy information is stored in the ACE database.

##### 1.2 Polymorphism Finder

This optional module determines if discrepancies are attributable to natural sequence variations of the gene, which is particularly relevant for human genes cloned using different tissue samples from those used to make the reference sequences. The process requires two steps: (1) a relatively short sequence segment comprising the discrepancy and its flanking sequence is compared using NCBI BLAST [25,26] to data from all user-selected GenBank databases to find an identical match; (2) each 100% hit is verified by comparing the entire clone target sequence with the hit sequence to ask if it came from the same gene using Pairwise BLAST [31]. A match provides good evidence that the observed sequence variation occurs naturally, but the absence of a match is inconclusive [32].

The Polymorphism Finder in ACE will analyze all discrepancies detected for a set of clones and store the NCBI GI number for each verified hit as part of the discrepancy description. As this operation requires sending numerous BLAST queries against large databases, its implementation requires a local copy of the appropriate GenBank database(s) installed on a dedicated high-performance computer or cluster.

##### 1.3 Decision Tool

This tool sorts clones into a finished-and-accepted group, a rejected group, and a set of groups slated for further processing. Fundamentally, the Decision Tool accomplishes this by comparing each clone's discrepancies list to a user-defined list of acceptance criteria, which define how many discrepancies of each type are permitted. Users can set thresholds to: (1) automatically accept clones that meet some high level criteria and (2) automatically reject clones that fail to meet some minimal criteria. Moreover, users can set independent thresholds for high and low confidence discrepancies (see Figure 2 and Additional file 1, Figure 3).

**Figure 5:**
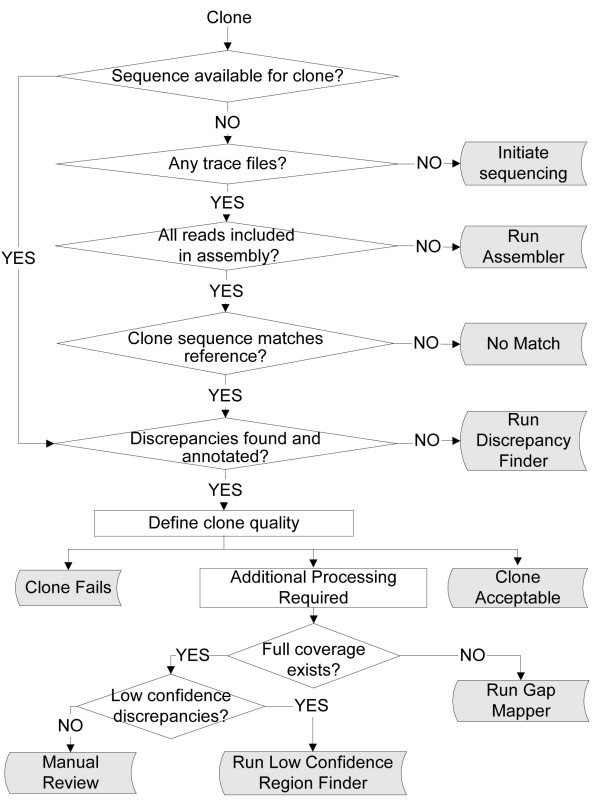
**Block diagram of Decision Tool**. This figure illustrates the logic used by the Decision Tool to assign clones to their various action groups indicated by the colored blocks. The tool will process any clones in the system. However, as the definition of clone quality is based upon comparing the list of discrepancies for the clone with the users' specifications for discrepancies, sorting clones to certain sub-groups requires a previous run through the Discrepancy Finder. Each action group is a separate tab-delimited list of clones that can be used to initiate the corresponding process.

Any clones not accepted nor rejected remain incomplete until additional processing has been performed. The flowchart in Figure 5 illustrates the logic path for assigning these pending clones to different action groups. The goal here is to bin clones into groups that require the same next step(s) in processing and report them in a single flat file [see Additional file 2, Pages 2 and 3]. This tab-delimited output can be used as input for the next processing step.

Users may wish to apply alternate acceptance criteria to the same set of clones for different experimental purposes. User specifications for acceptance criteria are stored as named sets in the software and can be invoked and applied to any collection of clones.

##### 1.4 Isolate Ranker

Some cloning workflows produce several isolates for the same gene with the expectation that at least one of these isolates will be of acceptable quality. Isolate Ranker selects the best isolate to carry forward by comparing isolates based on end reads, or (when available) partial or full sequencing data, and applying user defined penalties for different discrepancy types. For each combination < discrepancy type, confidence > (where "confidence" can be "low" or "high"), the user specifies two values: maximum permitted number of such discrepancies and the penalty per discrepancy combination [see Additional file 1, Figure 4]. Isolates that exceed the maximum permitted number of mutations of at least one type are rejected. For each remaining isolate, the overall score is computed by normalizing the sum of the penalties over the number of bases covered. These scores determine the rank order among the surviving isolates of the same gene and are displayed as a color coded virtual plate map (see Figure 3 and Additional file 1, Figure 1) so that users can quickly identify the best candidate clones.

#### 2. Clone Sequencing Support

The automation of clone sequence validation requires tools that manage the sequencing process itself. Unlike genomic sequencing projects that are often tasked with combining all available sequence reads into a single large contig, clone validation can be likened to thousands of independent micro-sequencing projects that must be maintained and managed separately.

##### 2.1 Trace File Distributor

**Figure 6:**
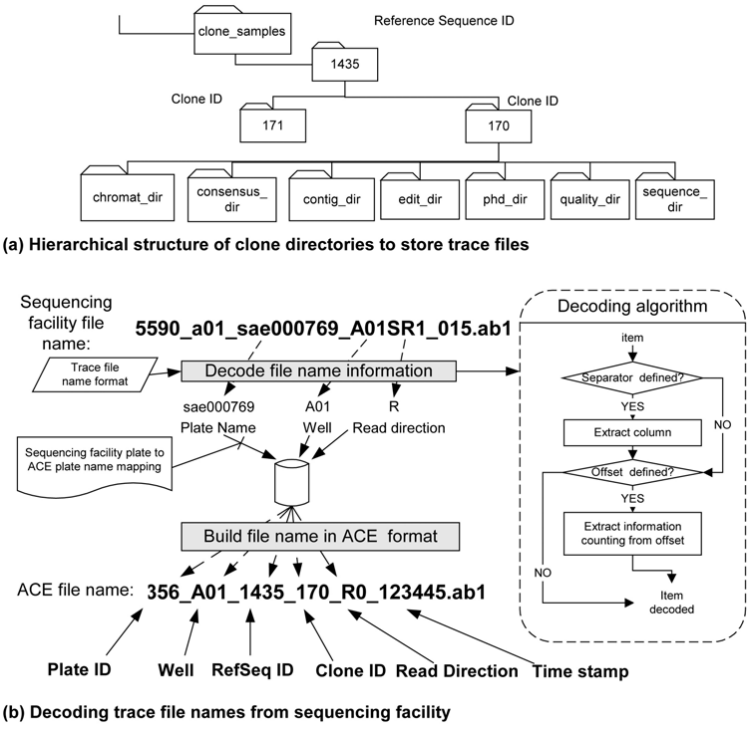
**Storage of trace file information**. (a) Hierarchical structure of reference sequence and clone directories. (b) Decoding trace file names from sequencing facility. Sequence trace filenames usually contain plate name, well and read direction in various configurations. The user describes the location of each of these items separately by specifying the separator, section number, offset inside each section and the expected length. The separator is a string (often a single character such as the underscore in this example) that may be repeated in the name, breaking it into several sections. The section number identifies the section that contains desired item; offset from the beginning of the section and length allow ACE to extract the item. When the sequencing facility applies its own name to the plate, it provides a lookup table indicating the correspondence between the users' and the sequencing facility's plate names.

Clone validation projects often include sequence reads from different clones representing similar genes (e.g., paralogs, multiple isolates of the same gene, etc.). It is essential that reads from closely related clones do not end up in one another's analysis. The ACE package creates a hierarchical directory structure and stores all files related to a single clone in a directory specific to that clone, as required by the Phred/Phrap package [7,8] (Figure 6a).

The Trace File Distributor parses identifier information encoded in the filename for each trace file, embedded there by the sequencing facility, that indicates which clone it belongs to (Figure 6b). However, because different facilities utilize different naming conventions, the Trace File Distributor stores each facility's format and uses it to convert the filename into a format that ACE can use to process these files automatically. This approach has worked efficiently for files from five different sequencing centers.

##### 2.2 End Read Processor

End reads are treated slightly differently from internal reads because some users employ them to select the best candidate from multiple isolates for a clone. We have found that poor quality end reads will lead to the rejection of about 25–35% of good clones based on discrepancies that turn out to be sequencing errors. To mitigate this the End Read Processor assesses read quality and disregards any end reads that do not meet minimum quality criteria, whereas end reads of sufficient quality are submitted into the database. To satisfy minimum quality criteria, a read must be longer than the user-defined minimum length (default = 250 nt) and the average confidence score for all non-ambiguous bases between the first and last base (default: first base = 50, last base = 700) must be above the user-defined minimum confidence score (default = 25). The trace files for the rejected end reads can be optionally added later as internal reads for inclusion in the clone sequence assembly.

##### 2.3 Assembly Wrapper

This tool automates contig assembly by calling the Phred/Phrap [7,8] package for every clone on a user-submitted list. Contig assembly for multiple clones can be a significant bottleneck in HT projects. When simultaneously assembling thousands of clones, experience has demonstrated that some fraction of clones will fail to assemble despite the availability of adequate sequence coverage. However, by adjusting a variety of settings involving trimming and quality requirements, many of them can be encouraged to assemble.

Vector trimming during contig assembly is performed using Cross_match [8]. In some cases, trimming is essential for contig assembly, whereas in others, it interferes with it. For example, a high degree of similarity between gene sequence and sequences in the vector library causes Phred/Phrap to mask valid (i.e. gene) sequence when vector trimming is applied blindly. This problem can be partially alleviated by editing vector sequences down to about 300 bp of insert-flanking sequence and removing irrelevant vector sequences from the vector library. Occasionally it is necessary to turn off vector trimming altogether to get an assembly for particular clones. We also found that aggressive quality- and/or read length-based trimming helps to improve contig assembly. When prompted, ACE trims all reads prior to assembly by removing bases prior to base 50 and after base 900 (user-adjustable values). Reads with a low average confidence score or below a minimum length can also be excluded from the assembly.

#### 3 Clone Finishing

For practical reasons, sequence coverage for validating clones is usually much lower than that used for *de novo *sequencing projects (typically several fold instead of 10 fold or more). Thus, failed reads lead readily to gaps in coverage or regions of low sequence confidence. As there are often hundreds to thousands of clones to track, software is needed to automate the finishing process. Autofinish[15] is excellent software for finishing in *de novo *sequencing projects. However, this program is not the optimal tool for the verification workflow, because it assumes dense coverage produced by shotgun sequencing and it does not exploit the existing reference sequence.

##### 3.1 Gap Mapper

**Figure 7:**
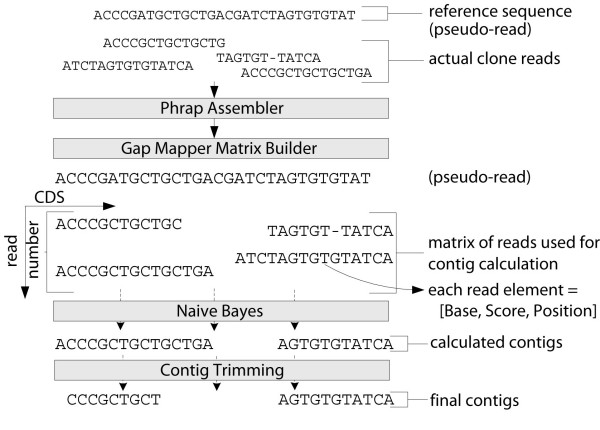
**Illustration of Gap Mapper algorithm for contig calculation**. Clone reads deemed to be of sufficient quality are supplemented by a pseudo read generated from the reference sequence with all bases set to a confidence score of 19 and submitted to the Phrap assembly process. The alignment matrix of clone reads is retrieved from Phrap output in order to determine the positions of each read relative to the reference sequence. Consensus sequences and confidence scores are computed for the various contigs using a naïve Bayes classifier, and the results are reported. The calculated contigs are trimmed to drop low confidence tails.

This module uses a strategy that exploits the existence of the reference sequence to demarcate gaps in sequence coverage that arise from short or failed reads [see Additional file 1, Figure 5]. The module uses the assembler to align clone trace files with the reference sequence, which is included as a "pseudo-read" with the same preset confidence score used for every base in its sequence (arbitrarily set at 19). This ensures complete assembly without forcing the contig to be identical to the target sequence. The assembler output is parsed to determine the alignment of each sequence read relative to the reference sequence. Using this positional information, a two-dimensional matrix is created wherein each position is described by base and confidence score (Figure 7). At each position along the matrix (which is numbered according to the reference sequence), a consensus base is determined for the clone by assessing all of the bases at that position and their confidence scores using a naïve Bayesian calculation [11]. The reference sequence and assembled contig are disregarded at this step, so the computed confidence scores reflect the actual clone sequence. The resulting contigs are optionally trimmed at both ends to remove bases with low confidence scores (using the sliding window algorithm described below). Tight trimming ensures that the new coverage will include the junction regions. Once the contigs are assembled, trimmed and mapped to the reference sequence, the gaps are defined as stretches of reference sequence not covered by contigs. Gap and contig information is stored in the database and used to assess clone status and quality, and can be passed to Primer Designer to design primers for clone finishing.

##### 3.2 Low Confidence Regions Finder

Not surprisingly, discrepancies most frequently occur where sequence confidence is low. The Low Confidence Regions Finder was designed to identify low confidence regions (LCRs) in contigs by applying a 'sliding window' algorithm. The user defines the width of the window (default = 25 nt), cut-off Phred score for low-confidence bases (default = 25) and maximum allowable number of low-confidence bases (default = 3 nt). Using the default values, an LCR is defined as a region in which a window of 25 consecutive bases contains at least three low-confidence bases. LCRs located close (default = 50 nt) to each other are joined [see Additional file 1, Figure 6]. Like gaps, LCRs can be processed by the Primer Designer to design a primer walk to obtain better coverage for these regions.

##### 3.3 Primer Designer

This set of modules exploits the availability of the reference sequence to allow users to: (a) design gene-specific sequencing primers; (b) select specific primer(s) for placement on a vendor order; (c) view all primers (both designed and ordered) and their design specifications using a convenient user interface, and (d) track and manage primer plates and individual primers. In practice, the Primer Designer tool is among the most utilized tools in this application. It can be used *a priori *to design gene-specific primers for a complete primer walk or, more efficiently, to design only those primers needed to complete coverage (i.e., fill in gaps) or to re-sequence regions of low confidence defined by Gap Mapper or Low Confidence Regions Finder. The module collects essential information including: (1) type of coverage desired (single forward, single reverse, double coverage, etc.); (2) primer sequence related parameters (T_m_, window size, GC content, etc.); (3) sequence processing parameters (length of reliable part of sequencing reads); and which sequences to cover [see Additional file 1, Figure 7]. After removing any sequence covered by universal primers (optional), the module breaks the remaining sequence into fragments which are provided to Primer3 [10] for primer prediction. Fragment size takes into account the expected distance between the primer and reliable sequence, expected high quality read length, and the Primer3 window size provided as part of user specification for Primer Designer. The Primer Design module then collates the output of Primer3.

## Results

In order to test the performance of ACE, we compared the processing times and outcomes for an experienced operator using ACE with a researcher experienced at manual analysis using commercial software to analyze one plate of clones for 93 unique genes ranging in length from 130 to 2800 bp flanked by common linkers of ~100 bp. We did not include project setup time for either study arm so that the focus was on processing time for the traces. To simplify the comparison, we designed and ordered internal sequencing primers for all clones with an insert length above 900 bp at the start of the project based on the reference sequences and a uniform read spacing (154 primers and sequence reactions were ordered for 45 clones). Although commonly used for manual analysis projects, pre-ordering all of the internal primers is not ordinarily needed for ACE projects. Instead, internal primers are typically ordered *as needed *based on the gaps identified which significantly reduces the need for additional primers and reads.

The acceptance criteria that were used are shown in Figure 2. Basically clones were allowed to have no more than a single amino acid change. These final acceptance criteria are indicated by the far left column (Pass → High Confidence) and were used by both analysis methods. The other 3 columns were used by the software to automatically reject unacceptable clones or to indicate clones that need further analysis.

### Manual analysis

The manual analysis was performed by an experienced researcher, who analyzed all clones using Sequencher™ (Gene Code) software and tracked the results in Excel^® ^(Microsoft). Each clone was individually analyzed and annotated according to the observations made at the time. Any internal or repeat reads for a clone were uploaded into the previously generated file and the analysis was repeated where necessary. Insufficient coverage and only high confidence discrepancies were annotated by indicating the position based on the entire target sequence (insert plus 5'/3' linker) where the first base of the 5' linker was listed as '1'. The researcher passed clones based on the criteria indicated above (up to one amino acid change and no truncation mutations); everything else was failed or listed as pending.

In summary, the researcher accepted 72 clones; 1 clone was provisionally accepted after confirming that an in-frame deletion could be found in other GenBank entries by BLAST analysis (see Table 2). 13 clones were rejected (1 did not match target sequence; all others had changes leading to truncations or frame shift mutations). There were 6 clones that did not have adequate quality sequence coverage to allow a decision about the insert. One additional clone had a region requiring additional sequencing due to ambiguous nucleotides that could not be resolved. To resolve these pending clones in the manual workflow, the researcher will need to manually design and order additional sequencing primers, as well as execute the necessary re-array to repeat the sequencing.

**Table 2:** Comparison of automated vs. manual analysis on a sample plate of 93 unique genes

	ACE		Researcher	
Analysis	Automatic + manual (20%)		Manual (100%)	
Samples	93		93	
Clones for:	Samples	Time	Samples	Time
End Read Analysis	93	45 min	93	270 min
Internal Analysis	45	30 min	45	100 min
Special Handling^#^	20	65 min	1	5 min
**Time Total**	**93**	**140 min**	**93**	**275 min**
				
**Outcome**	**Clones**	**%**	**Clones**	**%**
Accepted Clones	73	78.5%	73	78.5%
Rejected Clones	17	18.3%	13	14.0%
Pending Clones	3	3.2%	7	7.5%

#: special handling includes the manual analysis of 19 clones in the ACE workflow

### Automated ACE analysis

The same plate of clones analyzed manually was used for the automated analysis following the general workflow outlined in Figure 1. After sequence traces were loaded and those of acceptable quality were distributed into their appropriate clone directories, we assembled the end reads after trimming them based on the vector sequences, on the read length (omit bases < 50 or > 900), and on the base calling confidence (at least Phred score 20 in the assembly). We then performed a discrepancy analysis and used the isolate ranking feature to identify clones that did not match their reference. Based on the report for this initial processing, we culled all of the accepted clones and repeated the procedures for the remaining clones without vector or quality trimming. Altering the trimming parameters often captures clones that were missed in a first assembly pass. We used the same rules for automatic acceptance/rejection listed in Figure 2 (default settings).

In an ordinary project, it would be necessary at this point to design internal sequencing primers to cover any gaps or LCRs; however, as mentioned above, in this project all of the internal reads had been designed and pre-ordered based upon the reference sequence. Nevertheless, to capture the actual time needed to execute the project, we went through the exercise of identifying regions requiring additional coverage ('Gap Mapper') for clones that were either not fully covered with end reads due to the insert length ('No full Coverage') or listed as having 'Persistent Low Confidence Discrepancies'. We then designed internal primers theoretically needed to complete this coverage (37 primers, 7 clones), though these were not actually ordered from a vendor. This approach required less than one third of the number of primers and reads than were needed for the upfront design approach.

After uploading the pre-ordered internal reads and any repeated end reads into ACE, we performed a "first pass" analysis. This lead to 57 accepted clones, 17 rejected clones, and 19 that required further review (Table 2). Based on the report generated by ACE, 9 of the accepted clones had at least one discrepancy, but within the range allowed. Of the rejected clones, 5 exhibited no match to the target sequences and 12 had high quality discrepancies leading to frame shift or truncation mutations. The 19 clones slated for further review were all handled in Sequencher and resulted in acceptance of 16 additional clones. The remaining 3 clones had either regions that were not covered by sufficiently good traces (1 clone), or no traces of quality were present in the first place (2 clones).

## Discussion

When the outcome of the two analyses is compared, only a small number of clones show differences. The same clones were accepted by both approaches. One clone harboring an in-frame deletion was accepted because a BLAST analysis against GenBank indicated that it represents a documented variant for that gene. All clones manually rejected were also rejected in ACE; however, ACE rejected 4 additional clones that were not rejected in the manual analysis. In ACE these 4 clones were reported as being 'no match' with their target sequence, and upon automatic BLAST analysis in ACE were marked as showing only significant homology to the cloning vector. A review by a senior researcher confirmed the call made by ACE.

The somewhat higher rejection rate by the ACE software is fairly typical, as the parameters used for this study tuned the software to be conservative about accepting clones. The parameters demanded high sequence confidence across the clone and required resolution of all low confidence discrepancies. This has the effect that projects done with the automated workflow will end up with a subset of clones that require manual review. Obviously, by defining different parameter sets, users can employ less stringent criteria and obtain a different outcome. One of the advantages of using ACE is that every analysis has a well-defined and well-documented set of acceptance criteria attached to it. There are no ambiguities about why a clone was included or excluded.

The automated analysis took close to half the amount of time of the manual approach. This was surprising because such a large difference might not be expected for a small project (most projects using ACE have more than 1000 clones). As projects grow in size, the relative time savings grows considerably. The time it takes to analyze clones manually increases linearly with more clones. There is no advantage to increasing the scale; ten plates of clones take about 10 times longer than one plate of clones. However, this is not true for the automated analysis. Although there are some exceptions (e.g., approving internal primers), most of the ACE steps demonstrate no significant difference in the user operational time whether the operation is performed on one plate or ten. As the projects increase in size, the amount of researcher time spent per clone drops precipitously. Because the operational steps are straightforward, most large automated projects include a second round of automated analysis before referring pending clones for manual analysis, resulting in about 90% of the clones processed automatically. Because there were only 19 clones requiring further processing, all of them were done manually in this project.

There are some additional advantages to the automated analysis that might not be evident from this comparison. First, the automated analysis captures information on all clones including detailed annotation about all discrepancies for all available sequence. The manual review process focuses only on finding discrepancies that will cause a clone to be rejected. Once these are discovered, the clone is dropped and the researcher moves onto the next clone. Second, ACE produces a detailed report about all discrepancies and their polypeptide effects in a format that is easy to upload into a database for further analysis. This report is useful for understanding where mutations occur and improving cloning conditions. In cases of manual analysis one runs either the risk of human miss-annotations or having to export all assembled, finished sequences to cross-check them with a text comparison tool to create a standardized annotation for any discrepancy. Finally, ACE automatically manages the file transfers, the data tracking and the management of all internal sequencing primers. This includes automatically designing any necessary clone re-arrays for additional sequencing steps. In large scale projects, these processes are both very tedious and error prone when executed manually.

With the increasing construction of large cDNA and ORF clone sets slated for use in protein-based experiments comes the need to fully sequence validate the clones. Sequencher[33] and the Staden package[11,33] are widely used software tools for sequence assembly, editing and ORF analysis. Sequencher is a commercial desktop application which runs only on Windows and Macintosh OS. It was not designed to handle high-throughput processing for thousand clones and cannot be automated for this purpose. The Staden package has been developed to help automate the task of deriving a consensus sequence for genome sequencing efforts [8,9,34], but cannot be used to automate the process of validating plasmid clones. This is due, largely, to the fact that sequence derivation and sequence validation are two fundamentally different goals. Whereas *de novo *sequencing software focuses on producing the best consensus sequence, validation software must first determine a consensus sequence for each clone and then compare it to its appropriate existing reference sequence to determine whether the clone meets the user's standards.

To accomplish this goal, a novel strategy was employed in which each clone is described as a list of discrepancy objects. Essentially, this may be considered as alternative language to describe relationships between clone sequences and their reference standards.

In this strategy, clone validation is divided into the interplay between two separate processes: annotation and sorting. Annotation is an objective process that merely documents which discrepancies exist and what are their properties (location, polypeptide consequences, sequence confidence, polymorphism). In contrast, the sorting process is by its nature a subjective one. In this setting, each discrepancy becomes a liability for the clone that may be used to determine whether the clone should be rejected. The penalty appropriate for each type of discrepancy depends upon the intended experimental application. Indeed, even which attributes dominate the decision making process may vary – for some applications it is most important to achieve precise polypeptide matches whereas for others the emphasis is on clone sequences that occur naturally. Thus, the same objective list of discrepancies can be evaluated using different subjective scoring criteria.

The ACE software application was designed to meet the challenges of high-throughput clone verification projects. This software has dramatically reduced the amount of time and labor required to evaluate clone sequences, enabling many weeks worth of manual validation to be completed in a few hours. Moreover, the results are stored in a database that allows users to reassess the same set of clones based on different acceptance criteria and create detailed reports on accepted and rejected clones. For institutional reasons, we chose Oracle as a backend database for this project, though it could be adapted to other SQL-based databases. Nevertheless, given the number of clones, sequence traces, discrepancies and contigs that are typically tracked in these projects, some form of relational database is required. This is generally not a problem as most groups engaged in the validation of a large set of clones are likely to have database capabilities already.

An advantage of using automated tools is significantly reducing the number of missed sequence discrepancies, which can be overlooked by researchers during manual evaluation. However, the cost of this stringency is that the software will maintain some clones as "needs further review" (or occasionally rejected) that would otherwise get accepted by manual analysis, because human eyes can sometimes resolve errors made by the automated base caller. It is also true that for some sequence reads, manual analysis can resolve subtle issues (such as base compression or suppressed peaks) that are considered discrepancies by the automated tool. Although this problem can be mitigated to some extent by read trimming and by using confidence scores to indicate which sequences to trust, it is clear that there is still a role for manual analysis for about 10% of a typical large scale clone analysis project. Fortunately, these manual checks can be facilitated by ACE, which informs the user of which specific discrepancies and/or regions to check in order to expedite the manual review.

To accommodate the many possible workflows used in different projects, the ACE package was designed as a set of modules that can be invoked individually. For example, if full length sequences are obtained elsewhere, users can skip the sequencing workflow and proceed directly to contig analysis. The assembled sequences can be submitted as text files (with or without confidence scores) and compared to the expected sequences to generate a discrepancy list and sorted for acceptance (n.b., without confidence scores all discrepancies are assumed to be high confidence).

## Conclusion

The ACE software application was designed to meet the challenges of high-throughput clone verification projects. It uses a novel strategy to describe each clone as a list of multidimensional discrepancy objects that can be used to automatically determine the acceptability of the clone based on user defined acceptance criteria. Its major advantages include reducing the number of sequencing reads needed to achieve adequate coverage for making decisions on clones, reducing the need for manual analysis of numerous clone sequences, reducing the process time required to complete a project and significantly reducing human error when annotating discrepancies on nucleotide and protein level. ACE has being used by the Harvard Institute of Proteomics for finishing and validating of over 55,000 clones with ORFs ranging in size from 75 to 12,000 bp. Finished and validated clones are available to the community at Plasmid Information Database [35].

## Availability and requirements

Project name: ACE – Automated Clone Evaluation

**Project home page: **

Project help and installation instructions are supplied as an additional file [see Additional file 6].

Operating system(s): Linux: RedHat ; Windows: Microsoft Windows Server 2003

Programming language: Java, JSP, javaScript

**Other requirements: **Oracle 8i or 10g, Sun's J2SE version 1.4.1_02 or above, Tomcat 5.5.9, NCBI BLAST 2.0.14, EMBOSS 2.5.1 (for needle – global alignment program), Primer3 and Phred/Phrap package, Cygwin for installations on Windows server.

**Any restrictions to use by non-academics: **All the analysis tools are freely available for academics.

**Validated clone web page: **

## Authors' contributions

ET was the lead architect of the system, designed database schema and implemented application. AR was the lead alpha tester of this software who provided critical input on its accuracy and usability and suggestions for database design. JW and YH were additional alpha testers. DZ contributed to database design and manuscript revision. SM contributed to documentation for the software and manuscript revision. JL conceived of the project, conceptualized its overall structure, guided its development and edited this manuscript.

## Supplementary Material

Additional file 1 **Screen-shots of ACE interfaces**. Figure `. IsolateRanking Report. Figure 2. Request for Approval of Specific Primers. Figure 3. Decision Tool Execution. Figure 4. Create New Set ofParameters for Clone Ranking. Figure 5. Online example of Gap MapperResult. Figure 6. Online example of Low Confidence Region Finder Results. Figure 7. Parameter Settings for Sequencing Primer Design. Click here for file

Additional file 2 **Samples of ACE reports**. The file contains sample reports for Decision Tool, Primer Designer, Primer Order, Gap Mapper and Low Confidence Finder. Click here for file

Additional file 3 **ACE Data Import and Export**. The file contains the organization and type of data that is either imported into or exported from ACE. Click here for file

Additional file 4 **XML file with reference sequence descriptions**. Example of XML file contains descriptions of reference sequences for all clones on the plate 'YGS000374-1'. Click here for file

Additional file 5 **XML file with clone mapping information**. Example of XML file contains clone mapping data for the plate 'YGS000374-1'. Click here for file

Additional file 6 **Help file**. This is the pdf version of the online help files in the software. It includes both a tutorial and an overview of the software, as well as installation instructions. Click here for file
