# Supplementary material for: A novel approach to sequence validating protein expression clones with automated decision making
Source: BMC Bioinformatics. 2007 Jun 13;8:198. doi: 10.1186/1471-2105-8-198 (PMC1914086; doi:10.1186/1471-2105-8-198)
Supplement: Additional file 1 — Screen-shots of ACE interfaces. Figure `. IsolateRanking Report. Figure 2. Request for Approval of Specific Primers. Figure 3. Decision Tool Execution. Figure 4. Create New Set ofParameters for Clone Ranking. Figure 5. Online example of Gap MapperResult. Figure 6. Online example of Low Confidence Region Finder Results. Figure 7. Parameter Settings for Sequencing Primer Design. [file 1471-2105-8-198-S1.pdf]

# Additional File 1

# A novel approach to sequence validating protein expression clones with automated decision making

Elena Taycher[1], Andreas Rolfs[1], Yanhui Hu[1], Dongmei Zuo[1,2], Stephanie E. Mohr[2], Janice Williamson[1], Joshua LaBaer[1,2§]

[1] Harvard Institute of Proteomics, Harvard Medical School, 320 Charles St., Cambridge, MA 02141
[2] DF/HCC DNA Resource Core, Harvard Medical School, 320 Charles St., Cambridge, MA 02141

Contents

Additional File 1, Figure 1. Isolate Ranking Report ……………………………………….2
Additional File 1, Figure 2. Request for Approval of Specific Primers …………………3
Additional File 1, Figure 3. Decision Tool Execution……………………………………4
Additional File 1, Figure 4. Create New Set of Parameters for Clone Ranking………….5
Additional File 1, Figure 5. Online example of Gap Mapper Result……………………..6
Additional File 1, Figure 6. Online example of Low Confidence Region Finder Results..7
Additional File 1, Figure 7. Parameter Settings for Sequencing Primer Design …………8

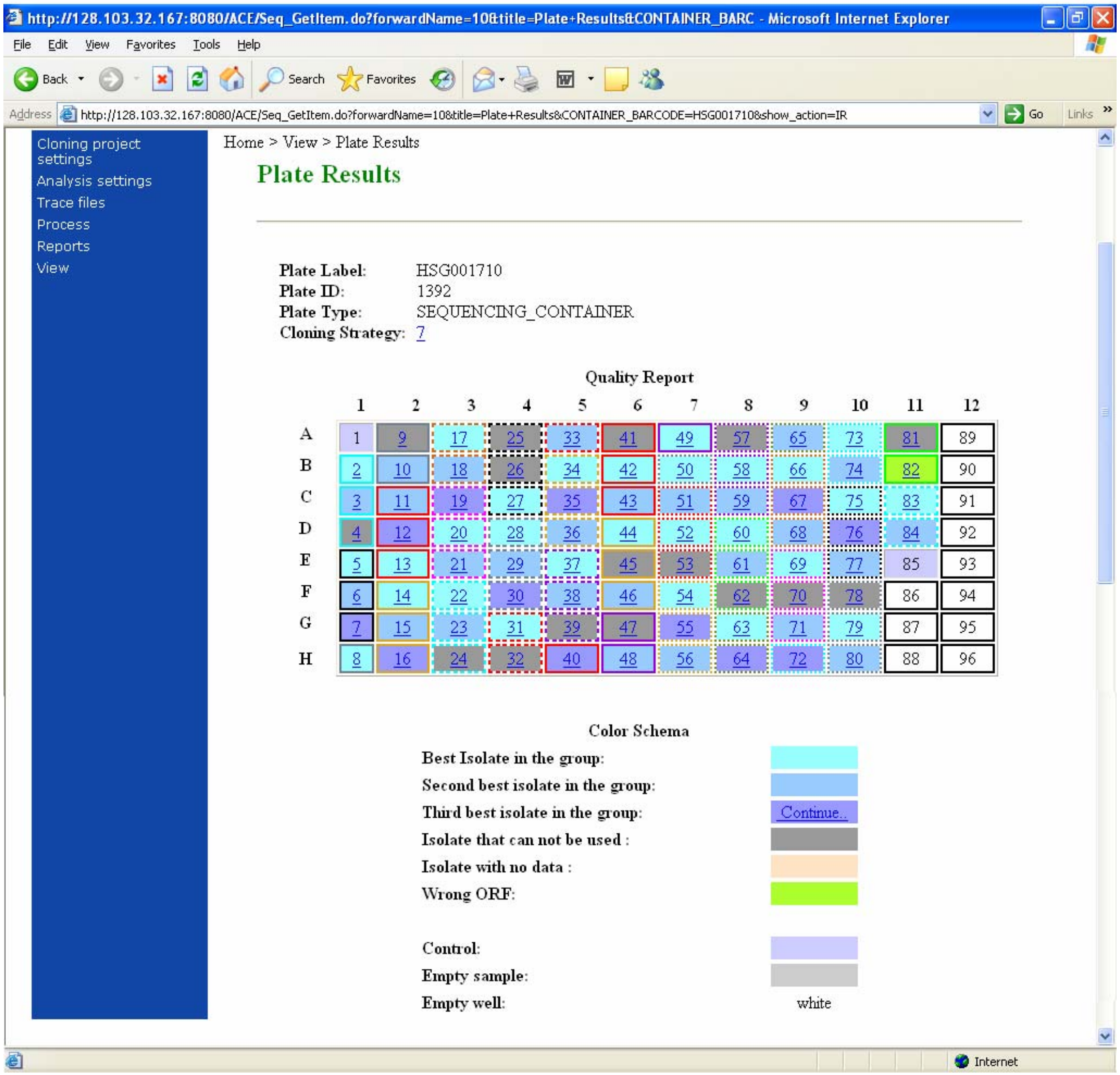

**Additional File 1, Figure 1. Isolate Ranking Report.**

Screenshot of ACE showing plate results of Isolate Ranker with color-coded clone rank. Identical border style (color and line style) is used to show wells that represent multiple isolates (clones) of the same gene. The color code is indicated below the plate map. As shown here, all isolates for the gene can be placed together, but it is not required. Clicking on any well number will open up a window with detailed information about the clone including the end reads, clone sequence and their alignments with the expected reference sequence.

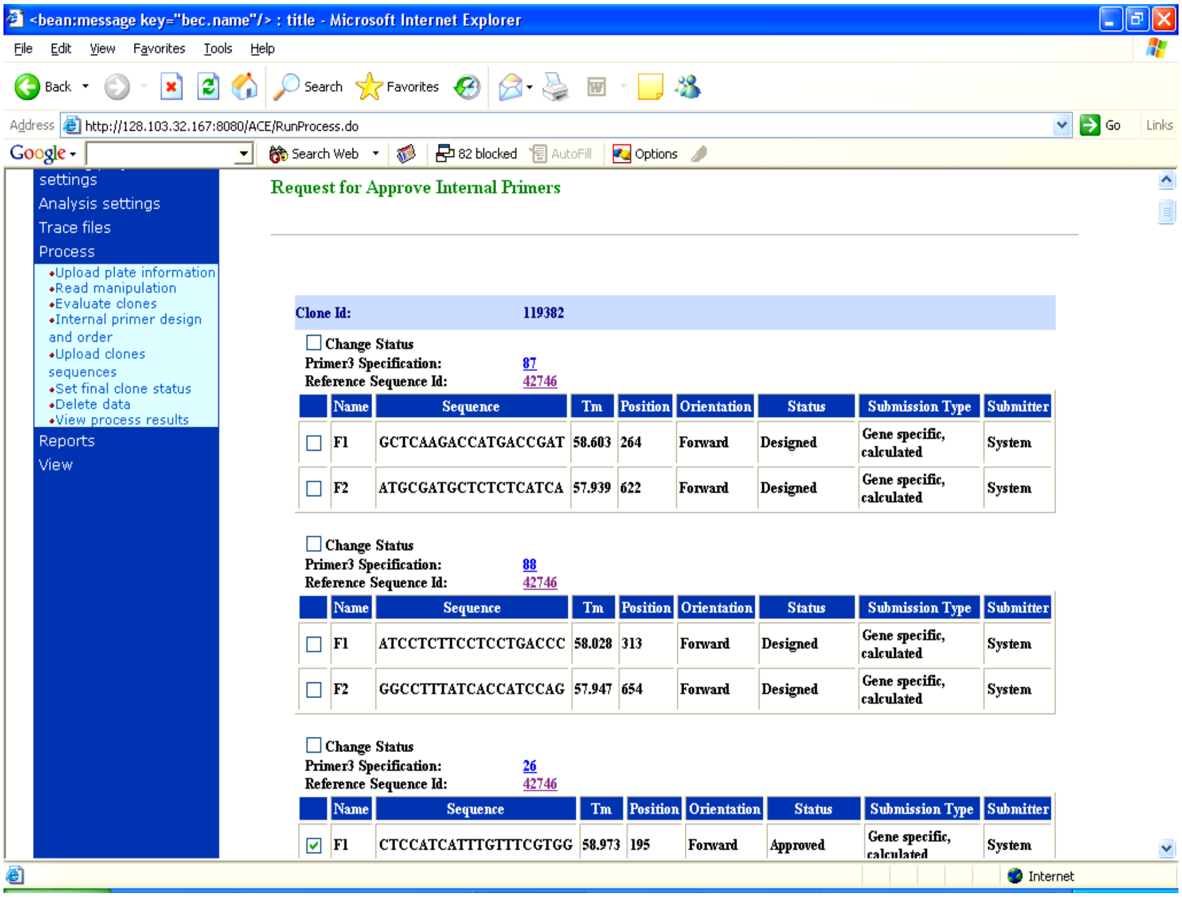


**Additional File 1, Figure 2. Request for Approval of Specific Primers.**

Screenshot shows the interface for selecting primers for the vendor order. ACE allows the user to design several sets of gene-specific primers for the clone using different Primer Designer specification. Primers from different sets can be put on the same vendor order. Upon placing the order ACE selects all primers approved for the specified clones and puts them in 96-well plate format. The user has an option to specify range of wells in which primers should be placed (preserving empty wells for controls if desired). ACE provides instructions for re-arraying the clone templates so that they match the vendor order and tracks all primers ordered for a particular clone.

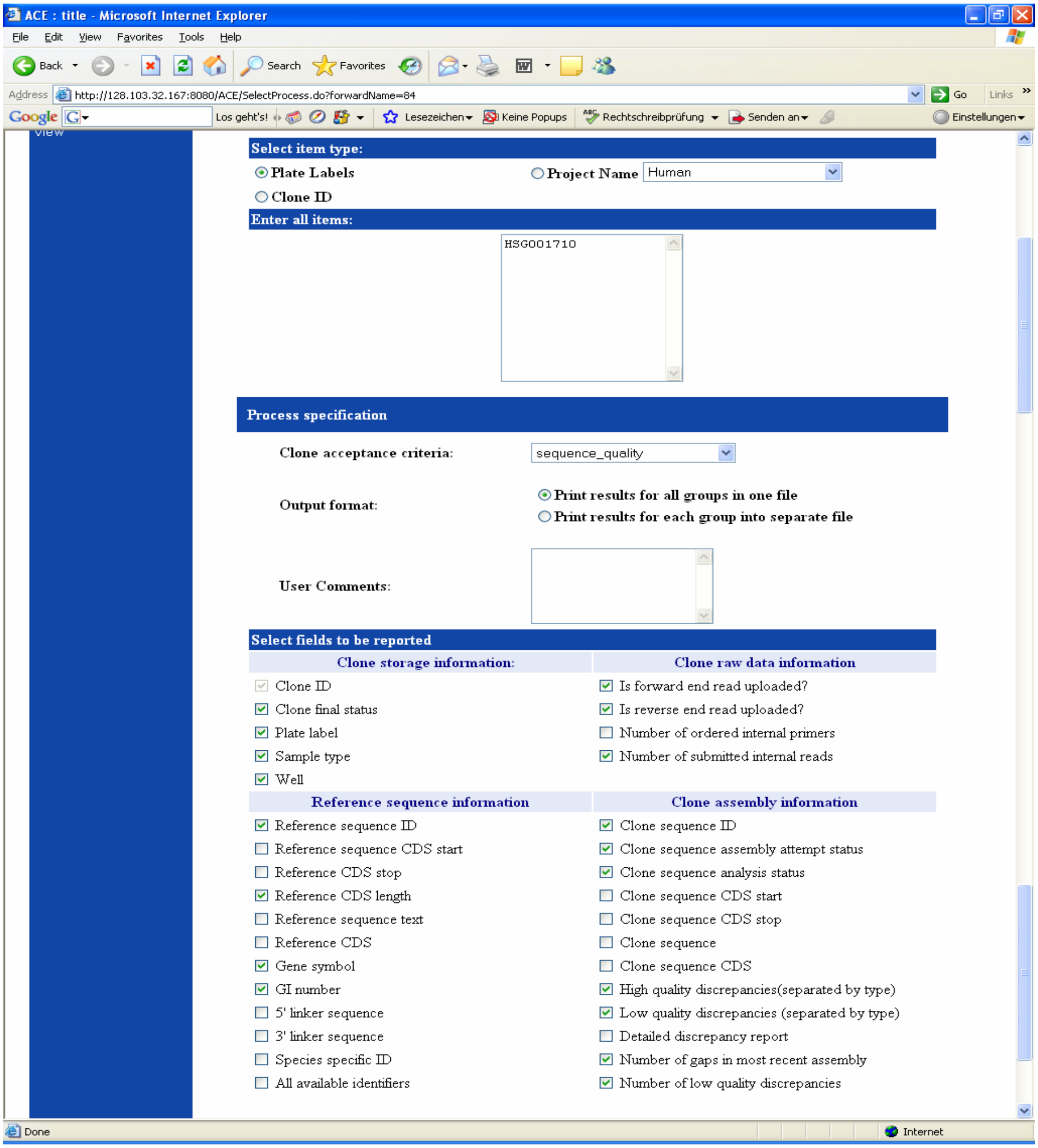


**Additional File 1, Figure 3. Decision Tool Execution.**

Screenshot of user interface that initiates execution of Decision Tool which sorts clones submitted for the analysis into appropriate categories (details in the manuscript). User can define a set of clones for analysis by providing: (1) plate labels; (2) unique clone ID(s); (3) specifying a project. Users invoke predefined acceptance criteria for the analysis by indicating the name of the saved set by selecting it from a pull down list of available sets (see text and Supplemental Figure 3). The output can be directed to separate files (useful for managing groups of clones for downstream steps) or to a single file (to obtain an overall project summary). Users can also specify which information they would like to include in the Decision Tool Report (e.g., clone sequence, clone CDS, discrepancy descriptions). An example of a decision tool report is included in the supplementary material.

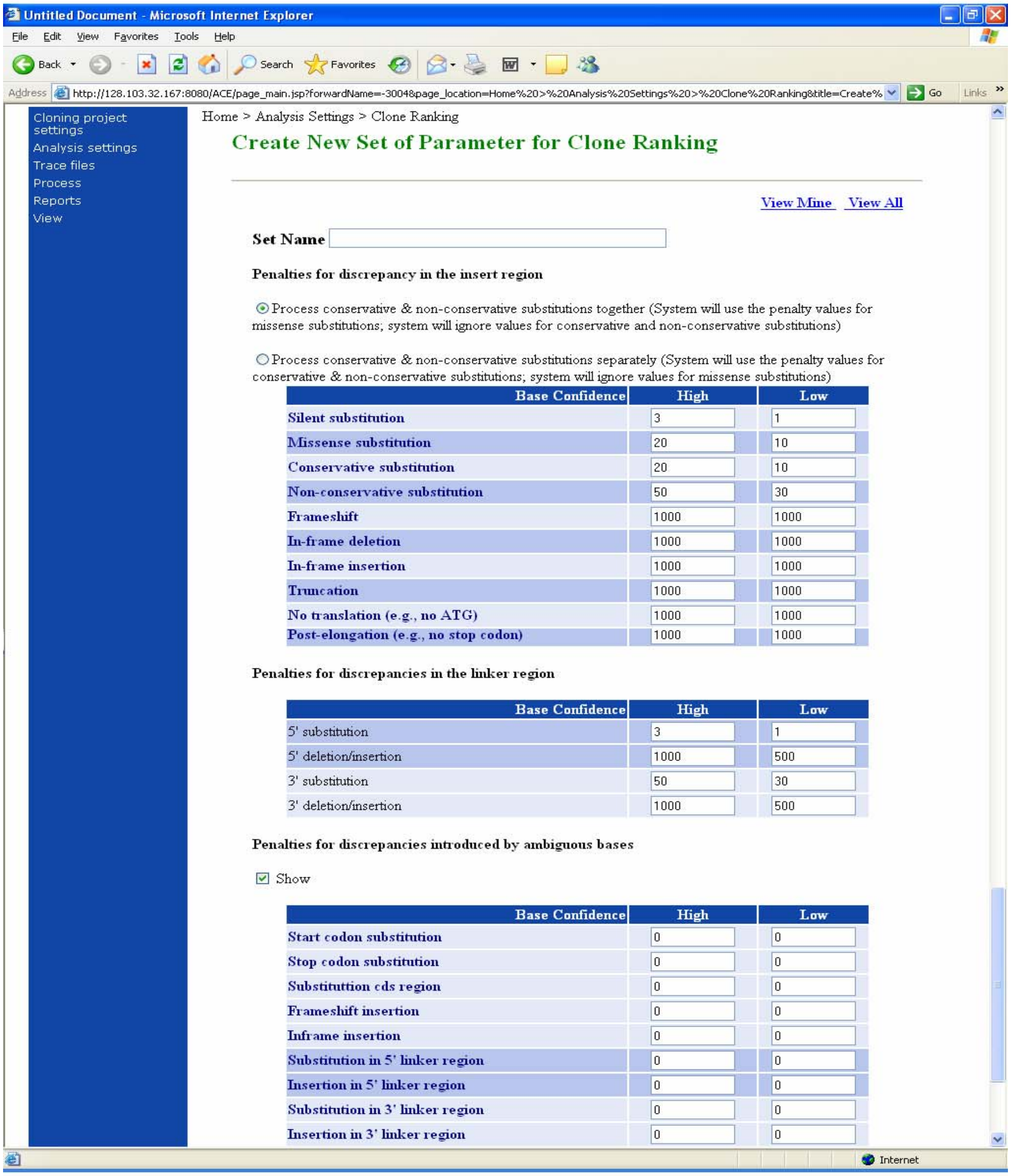


**Additional File 1, Figure 4. Create New Set of Parameters for Clone Ranking.** Screenshot of interface to create a set of parameters for isolate ranking. This is particularly relevant for workflows in which multiple isolates for each gene are selected with the intent to pick one “best candidate”. For each combination <discrepancy type, confidence> (where “confidence” can be “low” or “high”), the user specifies the penalty per discrepancy combination.

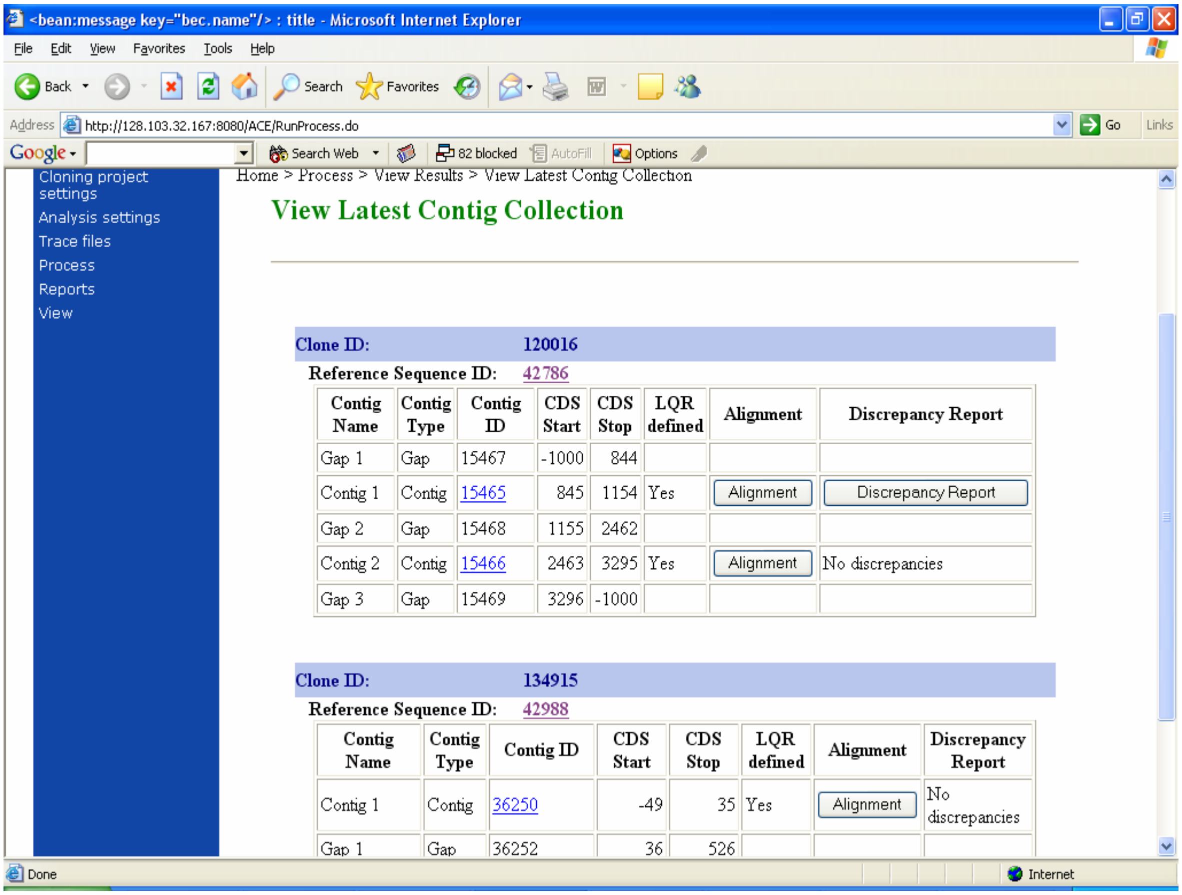


**Additional File 1, Figure 5. Online example of Gap Mapper Result.**

Screenshot shows contigs and gaps defined for a clone by Gap Mapper. Contigs are characterized by the position on the clone reference CDS and their sequences are analyzed by Discrepancy Finder to allow assessment of clone quality. LQR indicates Low Quality Region.

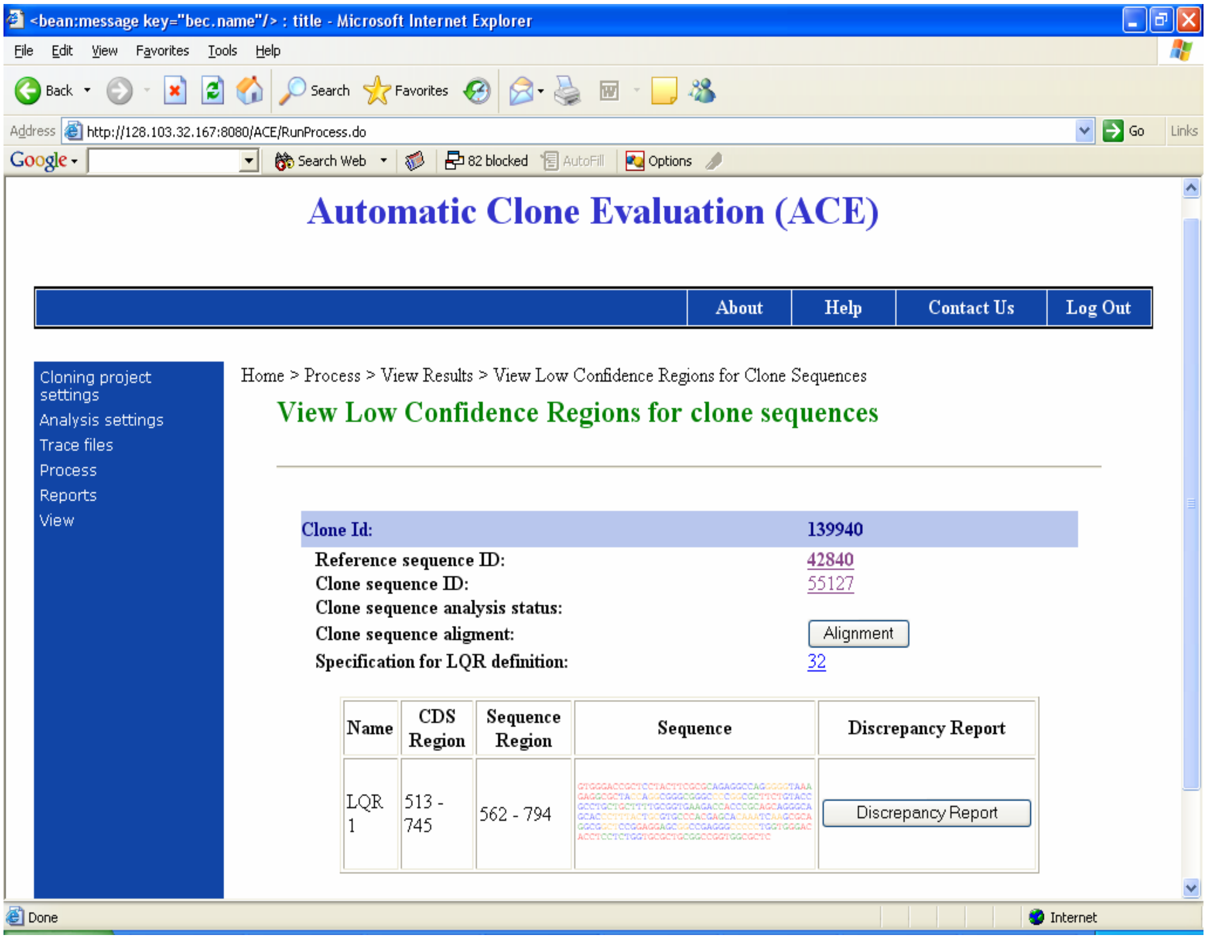


**Additional File 1, Figure 6. Online example of Low Confidence Region Finder Results.**

Screenshot shows example of low confidence regions for clone sequence. Low confidence regions are defined for a sequence by applying a ‘sliding window’ algorithm (see manuscript for details), each region is characterized by position on clone reference CDS.  Not viewable because it is at the bottom of the screen is the color code indicating the confidence score.

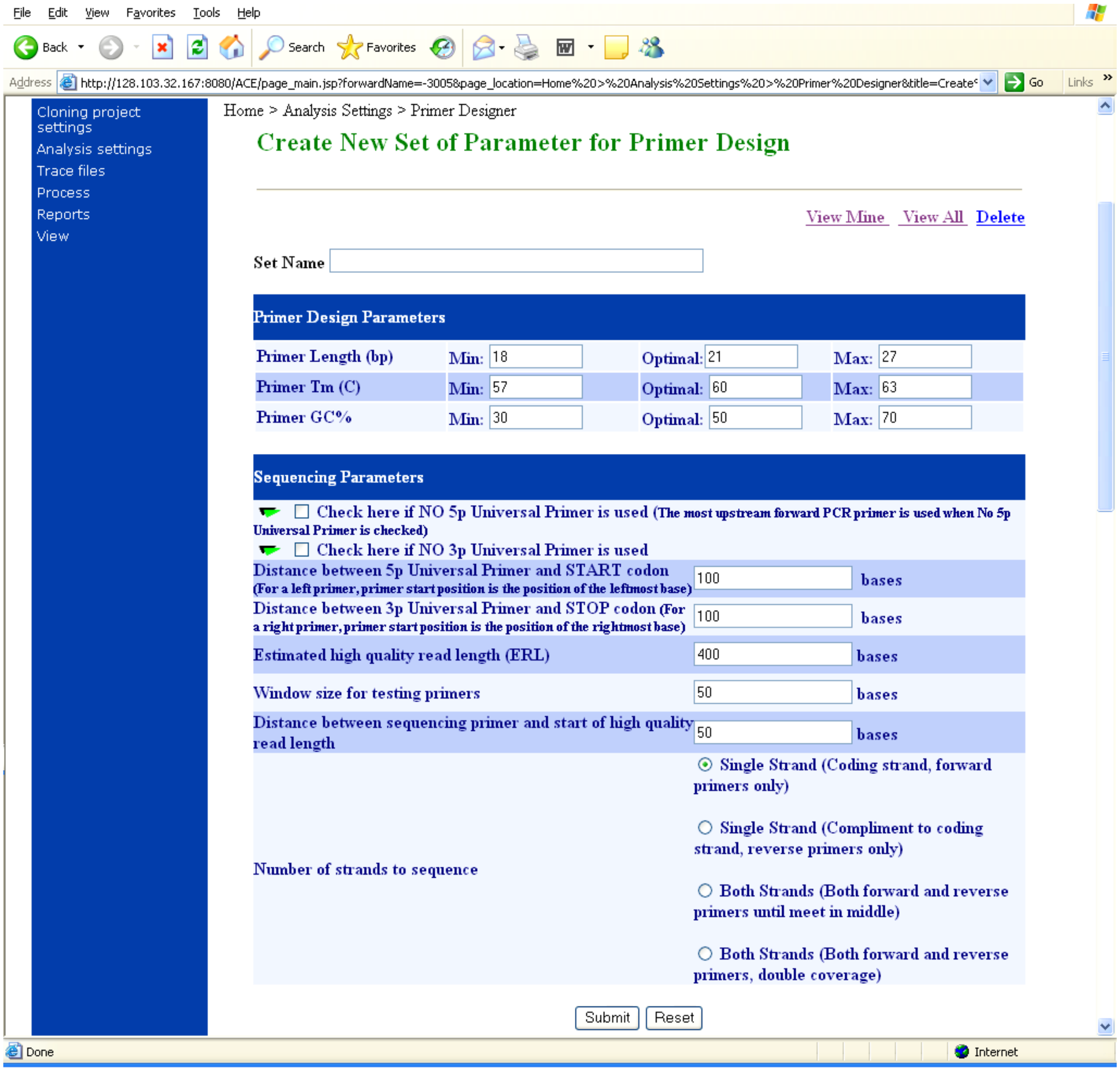


**Additional File 1, Figure 7. Parameter Settings for Sequencing Primer Design.**

Screenshot shows interface that allows users to create a set of parameters for primer design which include: (1) sequence related parameters (e.g., Tm, GC content); (2) sequence processing parameters (e.g., length of reliable part of sequencing reads); (3) type of desired coverage (e.g., single forward, single reverse, double coverage).
