# Supplementary material for: A novel approach to sequence validating protein expression clones with automated decision making
Source: BMC Bioinformatics. 2007 Jun 13;8:198. doi: 10.1186/1471-2105-8-198 (PMC1914086; doi:10.1186/1471-2105-8-198)
Supplement: Additional file 2 — Samples of ACE reports. The file contains sample reports for Decision Tool, Primer Designer, Primer Order, Gap Mapper and Low Confidence Finder. [file 1471-2105-8-198-S2.pdf]

Additional File 2

A novel approach to sequence validating protein expression clones with automated decision making

Elena Taycher[1], Andreas Rolfs[1], Yanhui Hu[1], Dongmei Zuo[1,2], Stephanie E. Mohr[2], Janice Williamson[1], Joshua LaBaer[1,2§]

[1] Harvard Institute of Proteomics, Harvard Medical School, 320 Charles St., Cambridge, MA 02141

[2] DF/HCC DNA Resource Core, Harvard Medical School, 320 Charles St., Cambridge, MA 02141

Contents

Decision Tool Summary …………………………………………………………….2
Decision Tool Report Details ……………………………………………………..…4
Low Confidence Finder Report…………………………………………………...6
Detailed Low Quality Finder Report……………………………………………..……7
Gap Mapper Report……………………………………………………………………..8
Primer Designer Report……………………………………………………………...9
Detailed Primer Information ………………………………………………………….12
Primer Order……………………………………………………………………..13

Decision Tool Summary

User ID: Tester
Date: 2-Feb-07
Comment:
User spec: Test_spec

Maximum acceptable number of discrepancies (gene region)

	High	Low	High	Low
Base Confidence:	High	Low	High	Low
Silent mutation	3	4	6	6
Conservative substitution	3	10	6	10
Nonconservative substitution	1	1	4	1
Frameshift	0	0	0	0
Inframe deletion	1	2	1	2
Inframe insertion	0	0	0	0
Truncation	0	0	0	0
No translation (e.g., no ATG)	0	0	0	0
Post-elongation(e.g., no stop codon)	0	0	0	0

Maximum acceptable number of discrepancies (linker region):

Base Confidence	High	Low	High	Low
5' substitution	1	5	3	5
5' deletion/insertion	1	1	1	1
3' substitution	1	5	3	5
3' deletion/insertion	1	1	1	1

Maximum acceptable number of discrepancies introduced by ambiquous bases

Base Confidence:	High	Low	High	Low
Start codon substitution	100	100	100	100
Stop codon substitution	100	100	100	100
Substituttion cds	100	100	100	100
Frameshift Insertion	100	100	100	100
Inframe Insertion	100	100	100	100
Substitution 5'	100	100	100	100
Insertion 5' linker	100	100	100	100
Substitution 3'	100	100	100	100
Insertion 3' linker	100	100	100	100

Submitted Items:
HSG001710
Total clone number: 85
Report Summary:

Accepted	62
Rejected	13
No trace files: Need further analysis	0
Assembly not attempted: Need further analysis	0
Clone data not analyzed: Need further analysis	0
No full sequence coverage: Need further analysis	7
Persistent LQ Discrepancy: Need further analysis	0
Other: Need further analysis	0

No match	1
Manul Review High Quality Discrepancies	0
Not clone sample	2

Decision Tool Report Details

Clone Id	Group	Next step	Clone Final Status	Plate Label	Sample Type	Position	Ref Sequence ID	Ref CDS Length	Gene Symbol	GI Number	Clone Sequence Id	Assembly attempt status - clone sequence	Analysis Status - clone sequence	5' High Quality Discrepancies	CDS High Quality Discrepancies	3' High Quality Discrepancies	5' Low Quality Discrepancies	CDS Low Quality Discrepancies	3' Low Quality Discrepancies	Forward End Reas Upload	Reverse End Reas Upload	Number of Internal Traces	Number of Gaps	Total Number of Low Quality Discrepancies
0	Not clone sample	None	In process	HSG001710	CONTROL_POSITIVE	1	N/A	N/A	N/A	N/A	N/A	N/A	N/A	N/A	N/A	N/A	N/A	N/A	N/A	N/A	N/A	N/A	N/A	N/A
141618	Accepted	Clone finished	Accepted	HSG001710	ISOLATE	2	31755	2492		15079680	112535	Assembled	Analyzed, no discrepancies found							Uploaded	Uploaded	7	N/A	0
141619	Accepted	Clone finished	Accepted	HSG001710	ISOLATE	3	31755	2492		15079680	112536	Assembled	Analyzed, no discrepancies found							Uploaded	Uploaded	7	N/A	0
141620	Rejected	Clone rejected	Rejected	HSG001710	ISOLATE	4	31755	2492		15079680	113009	Assembled	Analyzed, discrepancies found	Frameshift(High): 1|						Uploaded	Uploaded	8	N/A	0
141630	Accepted	Clone finished	Accepted	HSG001710	ISOLATE	5	34502	2708		21040398	112533	Assembled	Analyzed, no discrepancies found							Uploaded	Uploaded	8	N/A	0
141631	Accepted	Clone finished	Accepted	HSG001710	ISOLATE	6	34502	2708		21040398	112970	Assembled	Analyzed, no discrepancies found							Uploaded	Uploaded	8	N/A	0
141632	Accepted	Clone finished	In process	HSG001710	ISOLATE	7	34502	2708		21040398	112971	Assembled	Analyzed, discrepancies found	Silent Substitution(High): 1|						Uploaded	Uploaded	8	N/A	0
141642	Accepted	Clone finished	Accepted	HSG001710	ISOLATE	8	42580	1082	MAPK	17389605	97457	Assembled	Analyzed, no discrepancies found							Uploaded	Uploaded	0	N/A	0
141643	Rejected	Clone rejected	Rejected	HSG001710	ISOLATE	9	42580	1082	MAPK	17389605	97458	Assembled	Analyzed, discrepancies found	No translation(High): 1|						Uploaded	Uploaded	0	N/A	0
141644	Accepted	Clone finished	Accepted	HSG001710	ISOLATE	10	42580	1082	MAPK	17389605	97459	Assembled	Analyzed, no discrepancies found							Uploaded	Uploaded	0	N/A	0
141686	Accepted	Clone finished	Accepted	HSG001710	ISOLATE	11	34121	1148		18490285	113008	Assembled	Analyzed, no discrepancies found							Uploaded	Uploaded	2	N/A	0
141687	Accepted	Clone finished	In process	HSG001710	ISOLATE	12	34121	1148		18490285	111035	Assembled	Analyzed, discrepancies found	Conservative Substitution(High): 1|						Uploaded	Uploaded	1	N/A	0
141688	Accepted	Clone finished	Accepted	HSG001710	ISOLATE	13	34121	1148		18490285	97463	Assembled	Analyzed, no discrepancies found							Uploaded	Uploaded	0	N/A	0
141704	Accepted	Clone finished	Accepted	HSG001710	ISOLATE	14	33468	1148		17391431	113010	Assembled	Analyzed, no discrepancies found							Uploaded	Uploaded	2	N/A	0
141705	Accepted	Clone finished	Accepted	HSG001710	ISOLATE	15	33468	1148		17391431	112973	Assembled	Analyzed, no discrepancies found							Uploaded	Uploaded	1	N/A	0
141706	Accepted	Clone finished	Accepted	HSG001710	ISOLATE	16	33468	1148		17391431	97466	Assembled	Analyzed, no discrepancies found							Uploaded	Uploaded	0	N/A	0
172223	Accepted	Clone finished	Accepted	HSG001710	ISOLATE	17	20383	1445	CLK4	10190705	112978	Assembled	Analyzed, no discrepancies found							Uploaded	Uploaded	1	N/A	0
172224	Accepted	Clone finished	Accepted	HSG001710	ISOLATE	18	20383	1445	CLK4	10190705	111040	Assembled	Analyzed, no discrepancies found							Uploaded	Uploaded	3	N/A	0
172226	Accepted	Clone finished	In process	HSG001710	ISOLATE	19	29343	896	KHK	13623266	113015	Assembled	Analyzed, discrepancies found	Silent Substitution(High): 1|						Uploaded	Uploaded	2	N/A	0
172227	Accepted	Clone finished	Accepted	HSG001710	ISOLATE	20	29343	896	KHK	13623266	113016	Assembled	Analyzed, no discrepancies found							Uploaded	Uploaded	2	N/A	0
172228	Accepted	Clone finished	Accepted	HSG001710	ISOLATE	21	29343	896	KHK	13623266	113017	Assembled	Analyzed, no discrepancies found							Uploaded	Uploaded	2	N/A	0
172246	Accepted	Clone finished	Accepted	HSG001710	ISOLATE	22	29060	1505		13543325	112979	Assembled	Analyzed, no discrepancies found							Uploaded	Uploaded	3	N/A	0
172247	Accepted	Clone finished	Accepted	HSG001710	ISOLATE	23	29060	1505		13543325	112980	Assembled	Analyzed, no discrepancies found							Uploaded	Uploaded	2	N/A	0
172248	Rejected	Clone rejected	In process	HSG001710	ISOLATE	24	29060	1505		13543325	112981	Assembled	Analyzed, discrepancies found					Nonconservative Substitution(Low): 1|		Uploaded	Uploaded	2	N/A	1
172258	Rejected	Clone rejected	Rejected	HSG001710	ISOLATE	25	20617	941	PIM1	4505810	97472	Assembled	Analyzed, discrepancies found	Silent Substitution(High): 1|Nonconservative Substitution(High): 1|Truncation(High): 1|			5' deletion/insertion(Low): 1|			Uploaded	Uploaded	0	N/A	1
172259	No full sequence coverage: Need further analysis	Partial coverage Forward End read needed Run Gap Mapper	In process	HSG001710	ISOLATE	26	20617	941	PIM1	4505810	0	Assembly failed: more than one contig	N/A							Not uploaded	Not uploaded	1	No stretch collection defined	0
172260	Accepted	Clone finished	In process	HSG001710	ISOLATE	27	20617	941	PIM1	4505810	113014	Assembled	Analyzed, discrepancies found	Silent Substitution(High): 1|Nonconservative Substitution(High): 1|						Uploaded	Uploaded	2	N/A	0
141117	Accepted	Clone finished	Accepted	HSG001710	ISOLATE	28	40814	1394	CCNA	23271352	112967	Assembled	Analyzed, no discrepancies found							Uploaded	Uploaded	1	N/A	0
141118	Accepted	Clone finished	Accepted	HSG001710	ISOLATE	29	40814	1394	CCNA	23271352	112968	Assembled	Analyzed, no discrepancies found							Uploaded	Uploaded	2	N/A	0
141119	Accepted	Clone finished	Accepted	HSG001710	ISOLATE	30	40814	1394	CCNA	23271352	112969	Assembled	Analyzed, no discrepancies found							Uploaded	Uploaded	1	N/A	0
141125	Accepted	Clone finished	Accepted	HSG001710	ISOLATE	31	33396	293		16359017	97947	Assembled	Analyzed, no discrepancies found							Uploaded	Uploaded	0	N/A	0
141126	Rejected	Clone rejected	Rejected	HSG001710	ISOLATE	32	33396	293		16359017	97948	Assembled	Analyzed, discrepancies found	Frameshift(High): 1|						Uploaded	Uploaded	0	N/A	0
141127	Accepted	Clone finished	Accepted	HSG001710	ISOLATE	33	33396	293		16359017	97460	Assembled	Analyzed, no discrepancies found							Uploaded	Uploaded	0	N/A	0
141149	Accepted	Clone finished	Accepted	HSG001710	ISOLATE	34	34458	1427		21040479	111036	Assembled	Analyzed, no discrepancies found							Not uploaded	Uploaded	7	N/A	0
141150	Accepted	Clone finished	Accepted	HSG001710	ISOLATE	35	34458	1427		21040479	111037	Assembled	Analyzed, no discrepancies found							Not uploaded	Uploaded	7	N/A	0
141151	Accepted	Clone finished	Accepted	HSG001710	ISOLATE	36	34458	1427		21040479	112972	Assembled	Analyzed, no discrepancies found							Not uploaded	Uploaded	4	N/A	0
134468	Accepted	Clone finished	Accepted	HSG001710	ISOLATE	37	41140	1859	ELF	34191107	112974	Assembled	Analyzed, no discrepancies found							Not uploaded	Uploaded	7	N/A	0

134469	Accepted	Clone finished	Accepted	HSG001710	ISOLATE	38	41140	1859	ELF	34191107	111039	Assembled	Analyzed, no discrepancies found			Uploaded	Uploaded		4	N/A		0
134472	Rejected	Clone rejected	In process	HSG001710	ISOLATE	39	41140	1859	ELF	34191107	112977	Assembled	Analyzed, discrepancies found		Nonconservative Substitution(Low): 1|	Uploaded	Uploaded		4	N/A		1
134475	No full sequence coverage: Need further analysis	Partial coverag e Reverse End read needed Run primers design	In process	HSG001710	ISOLATE	40	41140	1859	ELF	34191107	0	Assembly pass: sequence is not submitted: cds not covered	N/A			Uploaded	Uploaded	1	0		0	
134476	Rejected	Clone rejected	In process	HSG001710	ISOLATE	41	41140	1859	ELF	34191107	112976	Assembled	Analyzed, discrepancies found	Silent Substitution(High): 1|	Frameshift(Low): 1|	Uploaded	Uploaded		4	N/A		1
134479	Accepted	Clone finished	Accepted	HSG001710	ISOLATE	42	41140	1859	ELF	34191107	112975	Assembled	Analyzed, no discrepancies found			Uploaded	Uploaded		3	N/A		0
134480	No full sequence coverage: Need further analysis	Partial coverag e Reverse End read needed Run primers design	In process	HSG001710	ISOLATE	43	41140	1859	ELF	34191107	0	Assembly pass: sequence is not submitted: cds not covered	N/A			Uploaded	Uploaded	4	0		0	
140012	Accepted	Clone finished	Accepted	HSG001710	ISOLATE	44	43947	1670	GPC	23398481	112960	Assembled	Analyzed, no discrepancies found			Uploaded	Uploaded		5	N/A		0
140013	Rejected	Clone rejected	In process	HSG001710	ISOLATE	45	43947	1670	GPC	23398481	112961	Assembled	Analyzed, discrepancies found		Inframe(Low): 1|Nonconservative Substitution(Low): 2|Frameshift(Low): 3|Truncation(Low): 1|	Uploaded	Uploaded		5	N/A		7
140014	Accepted	Clone finished	Accepted	HSG001710	ISOLATE	46	43947	1670	GPC	23398481	112962	Assembled	Analyzed, no discrepancies found			Uploaded	Uploaded		3	N/A		0
140042	Rejected	Clone rejected	Rejected	HSG001710	ISOLATE	47	34005	1673		17390084	0	Assembly pass: sequence is not submitted: cds not covered	N/A	Silent Substitution(High): 1|Frameshift(High): 1|		Uploaded	Uploaded	5	1		0	
140043	Accepted	Clone finished	Accepted	HSG001710	ISOLATE	48	34005	1673		17390084	112964	Assembled	Analyzed, no discrepancies found			Uploaded	Uploaded		5	N/A		0
140044	Accepted	Clone finished	Accepted	HSG001710	ISOLATE	49	34005	1673		17390084	112963	Assembled	Analyzed, no discrepancies found			Uploaded	Uploaded		3	N/A		0
135071	Accepted	Clone finished	In process	HSG001710	ISOLATE	50	32905	1610		15079459	112966	Assembled	Analyzed, discrepancies found	Silent Substitution(High): 1|		Uploaded	Uploaded		3	N/A		0
135142	No full sequence coverage: Need further analysis	Partial coverag e Reverse End read needed Run primers design	In process	HSG001710	ISOLATE	51	40988	1739	REL	20380824	0	Assembly pass: sequence is not submitted: cds not covered	N/A			Not uploaded	Uploaded	6	0		0	
135143	Accepted	Clone finished	Accepted	HSG001710	ISOLATE	52	40988	1739	REL	20380824	111034	Assembled	Analyzed, no discrepancies found			Not uploaded	Uploaded		4	N/A		0
135144	Rejected	Clone rejected	In process	HSG001710	ISOLATE	53	40988	1739	REL	20380824	112965	Assembled	Analyzed, discrepancies found		Frameshift(Low): 2|	Uploaded	Uploaded		3	N/A		2
172691	Accepted	Clone finished	Accepted	HSG001710	ISOLATE	54	18824	1154	MAPK 8	4506094	113021	Assembled	Analyzed, no discrepancies found			Uploaded	Uploaded		2	N/A		0
172692	Accepted	Clone finished	Accepted	HSG001710	ISOLATE	55	18824	1154	MAPK 8	4506094	97481	Assembled	Analyzed, no discrepancies found			Uploaded	Uploaded		0	N/A		0
172693	Accepted	Clone finished	Accepted	HSG001710	ISOLATE	56	18824	1154	MAPK 8	4506094	113022	Assembled	Analyzed, no discrepancies found			Uploaded	Uploaded		2	N/A		0
172767	Rejected	Clone rejected	Rejected	HSG001710	ISOLATE	57	28808	2630	DDR1	14250529	0	Assembly pass: sequence is not submitted: cds not covered	N/A	Frameshift(High): 1|		Uploaded	Uploaded	9	2		0	
172768	Accepted	Clone finished	Accepted	HSG001710	ISOLATE	58	28808	2630	DDR1	14250529	111041	Assembled	Analyzed, no discrepancies found			Not uploaded	Uploaded		7	N/A		0
172769	Accepted	Clone finished	Accepted	HSG001710	ISOLATE	59	28808	2630	DDR1	14250529	113020	Assembled	Analyzed, no discrepancies found			Uploaded	Uploaded		1 0	N/A		0
172167	Accepted	Clone finished	In process	HSG001710	ISOLATE	60	19092	704	TK2	10281329	113012	Assembled	Analyzed, discrepancies found	Conservative Substitution(High): 1|		Uploaded	Uploaded		2	N/A		0
172168	Accepted	Clone finished	In process	HSG001710	ISOLATE	61	19092	704	TK2	10281329	113013	Assembled	Analyzed, discrepancies found	Conservative Substitution(High): 1|		Uploaded	Uploaded		2	N/A		0
172169	No full sequence coverage: Need further analysis	Partial coverag e Forwar d End read needed Run Gap Mapper	In process	HSG001710	ISOLATE	62	19092	704	TK2	10281329	0	Assembly failed: more than one contig	N/A			Not uploaded	Not uploaded	0	No stretch collection defined		0	

172361	Accepted	Clone finished	Accepted	HSG001710	ISOLATE	63	26254	1112	FLJ12899	15489194	97477	Assembled	Analyzed, no discrepancies found							Uploaded	Uploaded	0	N/A		0
172362	Accepted	Clone finished	Accepted	HSG001710	ISOLATE	64	26254	1112	FLJ12899	15489194	113018	Assembled	Analyzed, no discrepancies found							Uploaded	Uploaded	2	N/A		0
172363	Accepted	Clone finished	Accepted	HSG001710	ISOLATE	65	26254	1112	FLJ12899	15489194	113019	Assembled	Analyzed, no discrepancies found							Uploaded	Uploaded	2	N/A		0
141742	Accepted	Clone finished	Accepted	HSG001710	ISOLATE	66	32668	1256		15079838	97467	Assembled	Analyzed, no discrepancies found							Uploaded	Uploaded	0	N/A		0
141743	Accepted	Clone finished	In process	HSG001710	ISOLATE	67	32668	1256		15079838	97468	Assembled	Analyzed, discrepancies found	Nonconservative Substitution(High): 1|						Uploaded	Uploaded	0	N/A		0
141744	Rejected	Clone rejected	In process	HSG001710	ISOLATE	68	32668	1256		15079838	123685	Assembled	Analyzed, discrepancies found				Silent Substitution(Low): 1|Frameshift(Low): 1|			Uploaded	Uploaded	2	N/A		2
135150	Accepted	Clone finished	Accepted	HSG001710	ISOLATE	69	43298	1754	KRT1	21961604	113003	Assembled	Analyzed, no discrepancies found							Uploaded	Uploaded	5	N/A		0
135151	Rejected	Clone rejected	In process	HSG001710	ISOLATE	70	43298	1754	KRT1	21961604	113004	Assembled	Analyzed, discrepancies found	Inframe Deletion(High): 1|						Uploaded	Uploaded	6	N/A		0
135152	Accepted	Clone finished	Accepted	HSG001710	ISOLATE	71	43298	1754	KRT1	21961604	111033	Assembled	Analyzed, no discrepancies found							Uploaded	Uploaded	9	N/A		0
135396	Accepted	Clone finished	In process	HSG001710	ISOLATE	72	42105	1205	TNFRSF11	33878056	113005	Assembled	Analyzed, discrepancies found	Nonconservative Substitution(High): 1|						Uploaded	Uploaded	2	N/A		0
135397	Accepted	Clone finished	Accepted	HSG001710	ISOLATE	73	42105	1205	TNFRSF11	33878056	97455	Assembled	Analyzed, no discrepancies found							Uploaded	Uploaded	0	N/A		0
135398	Accepted	Clone finished	Accepted	HSG001710	ISOLATE	74	42105	1205	TNFRSF11	33878056	97456	Assembled	Analyzed, no discrepancies found							Uploaded	Uploaded	0	N/A		0
172508	Accepted	Clone finished	Accepted	HSG001710	ISOLATE	75	26412	593	AK5	15214672	97483	Assembled	Analyzed, no discrepancies found							Uploaded	Uploaded	0	N/A		0
172509	Accepted	Clone finished	Accepted	HSG001710	ISOLATE	76	26412	593	AK5	15214672	97484	Assembled	Analyzed, no discrepancies found							Uploaded	Uploaded	0	N/A		0
172510	Accepted	Clone finished	Accepted	HSG001710	ISOLATE	77	26412	593	AK5	15214672	97485	Assembled	Analyzed, no discrepancies found							Uploaded	Uploaded	0	N/A		0
172624	No full sequence coverage: Need further analysis	Partial coverage Reverse End read needed Run primers design	In process	HSG001710	ISOLATE	78	29668	1964	SPHK2	13544054	0	Assembly pass: sequence is not submitted: cds not covered	N/A							Uploaded	Uploaded	7	0	0	
172625	Accepted	Clone finished	Accepted	HSG001710	ISOLATE	79	29668	1964	SPHK2	13544054	113023	Assembled	Analyzed, no discrepancies found							Uploaded	Uploaded	4	N/A		0
172626	Accepted	Clone finished	Accepted	HSG001710	ISOLATE	80	29668	1964	SPHK2	13544054	113024	Assembled	Analyzed, no discrepancies found							Uploaded	Uploaded	4	N/A		0
119982	No full sequence coverage: Need further analysis	Partial coverage Forward End read needed Run Gap Mapper	In process	HSG001710	ISOLATE	81	43712	3074	CARD1	21594975	0	Assembly pass: sequence is not submitted: no match or gaps in coverage	N/A							Not uploaded	Not uploaded	2	No stretch collection defined	0	
119983	No match	Run report 'show no match to the expected ORF'.	In process	HSG001710	ISOLATE	82	43712	3074	CARD1	21594975	N/A	N/A	N/A	N/A	N/A	N/A	N/A	N/A	N/A	N/A	N/A	N/A	N/A		
135252	Accepted	Clone finished	Accepted	HSG001710	ISOLATE	83	34234	2051		22713591	113006	Assembled	Analyzed, no discrepancies found							Uploaded	Uploaded	5	N/A		0
135253	Accepted	Clone finished	In process	HSG001710	ISOLATE	84	34234	2051		22713591	113007	Assembled	Analyzed, discrepancies found	Nonconservative Substitution(High): 1|						Uploaded	Uploaded	5	N/A		0
0	Not clone sample	None	In process	HSG001710	CONTROL_NEGATIVE	85	N/A	N/A	N/A	N/A	N/A	N/A	N/A	N/A	N/A	N/A	N/A	N/A	N/A	N/A	N/A	N/A	N/A	N/A	

Low Confidence Finder Report

Clone ID	Ref Sequence ID	CDS Length	Region Type	CDS Start	CDS Stop	LQR Start	LQR Stop
119565	42808	1412	LQR	535	787	584	836
119612	42773	1505	LQR	630	947	679	996

Detailed Low Quality Finder Report

Clone ID	Ref Sequence ID	CDS Length	Clone Sequence ID	Sequence Analysis Status	Discrepancy ID	Discrepancy Type	Quality	CDS start	Discrepancy Length	Forward Read Needed	Internal Reads Needed	Reverse Read Needed
119565	42808	1412	55117	ADY	125952	Silent	Low	687	1	No	Yes	No
119612	42773	1505	55108	ADY	125768	Frameshift: Insertion	Low	726	0	No	Yes	No
119612	42773	1505	55108	ADY	125770	Amb. substitution	Low	865	1	No	Yes	No

Gap Mapper Report

Clone ID	Ref Sequence ID	CDS Length	Region Type	CDS Start	CDS Stop	Sequence ID
134915	42988	1319	Contig	-49	34	206907
134915	42988	1319	LQR	17	34	206907
134915	42988	1319	Gap	35	549	N/A
134915	42988	1319	Contig	550	-<br>1000	206908
134915	42988	1319	LQR	550	600	206908

Primer Designer Report

Clone ID	119721
Ref Sequence Length	1799
Ref Sequence ID	40842

Primer3 Spec ID 23	Name	Position	Sequence	Tm	Direction
(Forward only)	F1	225	CTGGCTCCGGAATTCACT	58.7	Forward
	F2	490	ACCAAAGGGAAGAAGCAGTT	57.9	Forward
	F3	806	CTGTGTTGATGCACTACCCC	59.0	Forward
	F4	1095	GTTCGGTGTCCAGTTCCAATAC	60.7	Forward
	F5	1387	AATGAGTACCGCAAGAGGTTT	57.9	Forward

Primer3 Spec ID 36	Name	Position	Sequence	Tm	Direction
(Both forward and reverse	F1	10	GAGTCTCTTGCTCCGGTTC	57.5	Forward
primers, double coverage)	F2	304	TGTCAATGCCACCTTCATC	57.9	Forward
	F3	611	TTCACCCACCAGTTCTTCA	57.5	Forward
	F4	910	CTGGCTACGTGAGCACAAC	58.0	Forward
	F5	1235	ATGTTGGTGGACTATGGGG	58.1	Forward
	R1	1477	ATTCCTCCAACTCTGCTGC	58.0	Reverse
	R2	1190	CCCACCTTGAAGGAGTCAG	58.2	Reverse
	R3	900	ATACAGCATGAGCCCAGGA	59.2	Reverse
	R4	590	AACATGAGGTTGGTGCCTT	58.0	Reverse

Primer3 Spec ID 42	Name	Position	Sequence	Tm	Direction
(Both forward and reverse	F1	10	AGTCTCTTGCTCCGGTTCTT	58.1	Forward
primers, single coverage)	F2	225	CTGGCTCCGGAATTCACT	58.7	Forward
	F3	410	GCTGGGAGTCTTTCTCCAAC	58.9	Forward
	F4	607	CACTTCACCCACCAGTTCTT	57.6	Forward
	F5	806	CTGTGTTGATGCACTACCCC	59.0	Forward
	R5	957	GTGCTCAGCCTTCAGCAG	58.3	Reverse

	Name	Position	Sequence	Tm	Direction
	R4	1160	TGCCAGTGGTAGAGATGGTT	58.2	Reverse
	R3	1361	GACTCCCTGATGACATCCAC	57.8	Reverse
	R2	1580	GGAGCCCCAATCTCTATCAT	58.0	Reverse
	R1	1764	ATCATCCTGACTGGCATCC	58.4	Reverse

Primer3 Spec ID 40 (Both forward and reverse primers, single coverage)	Name	Position	Sequence	Tm	Direction
	F1	108	TTACTATCCATGCCAGCACC	58.6	Forward
	F2	299	AGTTTGTCAATGCCACCTTC	57.6	Forward
	F3	489	AACCAAAGGGAAGAAGCAGT	57.9	Forward
	F4	688	GGCCACATTTATGGAGACAA	58.4	Forward
	R4	1116	GTATTGGAACTGGACACCGA	58.4	Reverse
	R3	1290	AGCAATCTGGCGAGAGAAG	58.3	Reverse
	R2	1529	TTTTCAAGAAGCAGTCCAGG	58.1	Reverse
	R1	1723	GACAGGTCTTGGTGTTGAGG	58.1	Reverse

Primer3 Spec ID 47 (Both forward and reverse primers, single coverage)	Name	Position	Sequence	Tm	Direction
	F1	90	GCCAGTGAATCCCTGTTG	57.5	Forward
	F2	225	CTGGCTCCGGAATTCACT	58.7	Forward
	F3	356	GCTCCAACCTTATCCCCA	58.4	Forward
	F4	506	AGTTGCCAGATGCCCAG	58.2	Forward
	F5	687	CGGCCACATTTATGGAGA	58.0	Forward
	F6	809	TGTTGATGCACTACCCCC	57.8	Forward
	R6	986	TGGAAAAGCTGCTCATCG	58.6	Reverse
	R5	1144	GGTTGAACTCCATGGCAA	57.9	Reverse
	R4	1287	AATCTGGCGAGAGAAGGC	58.0	Reverse
	R3	1439	AGCTCCTGGAAGGAGGTG	57.9	Reverse
	R2	1600	GGAGACCCTTGAGGGAAA	57.5	Reverse
	R1	1740	ACGGAAGGAAACGTAGGG	57.6	Reverse

Primer3 Spec ID 62 (Both forward and reverse	Name	Position	Sequence	Tm	Direction
	F1	124	CACCAGGGCATCTGTGTC	58.5	Forward

	Name	Position	Sequence	Tm	Direction
primers, double coverage)					
	F2	305	TCAATGCCACCTTCATCC	57.9	Forward
	F3	506	AGTTGCCAGATGCCCAG	58.2	Forward
	F4	730	CGGCTCTTTAAGGATGGG	58.2	Forward
	F5	935	GTGACCTGCTGAAGGCTG	58.0	Forward
	F6	1104	CCAGTTCCAATACCGCAA	58.0	Forward
	F7	1318	GACCACCACATCCTGCAT	57.7	Forward
	F8	1509	CCCTGGACTGCTTCTTGA	57.3	Forward
	R1	1600	GGAGACCCTTGAGGGAAA	57.5	Reverse
	R2	1354	TGATGACATCCACAGCCA	57.9	Reverse
	R3	1191	GCCCACCTTGAAGGAGTC	58.1	Reverse
	R4	957	GTGCTCAGCCTTCAGCAG	58.3	Reverse
	R5	777	TTCTCCATCCAGCACCTG	58.2	Reverse
	R6	588	CATGAGGTTGGTGCCTTG	58.6	Reverse
	R7	373	TGGGGATAAGGTTGGAGC	58.4	Reverse
Primer3 Spec ID 82	Name	Position	Sequence	Tm	Direction
(Reverse only)	R1	1397	CGGTACTCATTGAAGGGCT	57.8	Reverse
	R2	986	TGGAAAAGCTGCTCATCG	58.6	Reverse
	R3	590	AACATGAGGTTGGTGCCTT	58.0	Reverse
Primer3 Spec ID 84	Name	Position	Sequence	Tm	Direction
(Both forward and reverse primers, single coverage)	F1	10	GAGTCTCTTGCTCCGGTTCTT	60.0	Forward
	F2	410	AGCTGGGAGTCTTTCTCCAAC	59.9	Forward
	F3	805	GCCTGTGTTGATGCACTACC	59.2	Forward
	R3	991	CGTCTGGAAAAGCTGCTCAT	60.5	Reverse
	R2	1362	GACTCCCTGATGACATCCACA	61.0	Reverse
	R1	1766	CATCATCCTGACTGGCATCC	61.5	Reverse

Detailed Primer Information

Oligo Sample ID	Plate Name	Well ID	Well Index	Clone ID	Primer ID	Primer Sequence	Tm	Primer Length
219531	OPLATE001013	2	B01	251474	212777	TTAAGCCTGGCAAATGTCTC	58.0	20
219532	OPLATE001013	3	C01	251474	212778	TGGCGATTGCTTTCTTCTTA	58.7	20
219533	OPLATE001013	4	D01	251474	212779	CGGAGGCAGATCTGTTCTTA	58.0	20
219534	OPLATE001013	5	E01	251474	212780	AGTGACGGATTTAGTGGCAG	57.8	20
219535	OPLATE001013	6	F01	251474	212781	ACAGATTCTGCAAGCGTTTC	58.1	20
219536	OPLATE001013	7	G01	251474	212782	CGAATGGCCACAGCTTAATA	58.8	20
219537	OPLATE001013	8	H01	251474	212783	CAATCAGGCATTGATGAAGA	57.2	20
219538	OPLATE001013	9	A02	251474	212784	AAGCAAGTAATTTGCCGATG	57.9	20
219539	OPLATE001013	10	B02	251474	212785	GAGGTAATTCGCTCTGGTGA	57.9	20
219540	OPLATE001013	11	C02	251474	213623	ACTGTTGTTGATCTTGCGAA	56.9	20
219541	OPLATE001013	12	D02	251214	212795	AATTTAAATCACATTGCGGG	57.5	20
219542	OPLATE001013	13	E02	251214	212796	TTACTTGCGTTAGGGCATTC	57.9	20
219543	OPLATE001013	14	F02	251214	212797	GATTCAAAATGCAACAGCGT	58.8	20
219544	OPLATE001013	15	G02	251214	212798	GGGCAATTGGATTAAAGGAT	57.9	20
219545	OPLATE001013	16	H02	251214	212799	ACAAAGGGCAATAAATTGGT	56.2	20
219546	OPLATE001013	17	A03	251214	212800	GGGAGAATCATGCCACAGTA	58.5	20
219547	OPLATE001013	18	B03	251214	212801	CCAGGTGCGTTTTATATTAGG	56.5	21
219548	OPLATE001013	19	C03	251214	213632	GATGATTGAAGAGAATGCCC	57.1	20
219549	OPLATE001013	20	D03	251216	213642	CACTTCTTCAAAACGCTCG	57.2	19
219550	OPLATE001013	21	E03	251216	213641	GTCATCACCTGCGATAAACA	57.1	20
219551	OPLATE001013	22	F03	251216	213640	AGGTTGTACACCTTCTTCGC	56.9	20
219552	OPLATE001013	23	G03	251216	213639	TGCTTAATGAACGGGATTTT	57.3	20
219553	OPLATE001013	24	H03	251216	213638	GAGAAGGAAAACGACCTTCA	57.0	20
219554	OPLATE001013	25	A04	251216	213637	GCATCGTAAAGGAAGAACGA	58.0	20
219555	OPLATE001013	26	B04	251216	213636	AAGAAGTGTGGGGCAGAATA	57.3	20
219556	OPLATE001013	27	C04	251216	213635	CAGGTAAATCTTTTCGCACA	56.5	20

Primer Order

Plate Name	Row	Column	Oligo Sample ID	Primer Sequence
OPLATE001013	A	01	*****	.
OPLATE001013	B	01	219531	TTAAGCCTGGCAAATGTCTC
OPLATE001013	C	01	219532	TGGCGATTGCTTTCTTCTTA
OPLATE001013	D	01	219533	CGGAGGCAGATCTGTTCTTA
OPLATE001013	E	01	219534	AGTGACGGATTTAGTGGCAG
OPLATE001013	F	01	219535	ACAGATTCTGCAAGCGTTTC
OPLATE001013	G	01	219536	CGAATGGCCACAGCTTAATA
OPLATE001013	H	01	219537	CAATCAGGCATTGATGAAGA
OPLATE001013	A	02	219538	AAGCAAGTAATTTGCCGATG
OPLATE001013	B	02	219539	GAGGTAATTCGCTCTGGTGA
OPLATE001013	C	02	219540	ACTGTTGTTGATCTTGCGAA
OPLATE001013	D	02	219541	AATTTAAATCACATTGCGGG
OPLATE001013	E	02	219542	TTACTTGCGTTAGGGCATTC
OPLATE001013	F	02	219543	GATTCAAAATGCAACAGCGT
OPLATE001013	G	02	219544	GGGCAATTGGATTAAAGGAT
OPLATE001013	H	02	219545	ACAAAGGGCAATAAATTGGT
OPLATE001013	A	03	219546	GGGAGAATCATGCCACAGTA
OPLATE001013	B	03	219547	CCAGGTGCGTTTTATATTAGG
OPLATE001013	C	03	219548	GATGATTGAAGAGAATGCCC
OPLATE001013	D	03	219549	CACTTCTTCAAAACGCTCG
OPLATE001013	E	03	219550	GTCATCACCTGCGATAAACA
OPLATE001013	F	03	219551	AGGTTGTACACCTTCTTCGC
OPLATE001013	G	03	219552	TGCTTAATGAACGGGATTTT
OPLATE001013	H	03	219553	GAGAAGGAAAACGACCTTCA
OPLATE001013	A	04	219554	GCATCGTAAAGGAAGAACGA
OPLATE001013	B	04	219555	AAGAAGTGTGGGGCAGAATA
OPLATE001013	C	04	219556	CAGGTAAATCTTTTCGCACA
OPLATE001013	D	04	*****	.
OPLATE001013	E	04	*****	.
OPLATE001013	F	04	*****	.
OPLATE001013	G	04	*****	.
OPLATE001013	H	04	*****	.
