# Supplementary material for: A novel approach to sequence validating protein expression clones with automated decision making
Source: BMC Bioinformatics. 2007 Jun 13;8:198. doi: 10.1186/1471-2105-8-198 (PMC1914086; doi:10.1186/1471-2105-8-198)
Supplement: Additional file 3 — ACE Data Import and Export. The file contains the organization and type of data that is either imported into or exported from ACE. [file 1471-2105-8-198-S3.pdf]

Additional File 3, Table 1. ACE Data Import and Export.

Data are submitted into ACE organized by the multi-well plates they are in and exported as a collection of individual clones.

Imported / exported data structures		
Data structure	Data element	Element description
Reference sequence description	Sequence text	Reference sequence text
	CDS start	Position of CDS start
	CDS stop	Position of CDS stop
	Sequence annotations	Collection of public available annotations (GI, Accession Number,…)
Clone plate description	Project name	Project name. Project defined as set of plates
	Cloning strategy description	Description of cloning strategy
	Plate name	Plate name
	List of clones	Collection of clones sitting on the plate
Clone description	Clone ID	Unique clone ID
	Plate position	Well position for the clone
	Link to reference sequence	Link to reference sequence information, several isolates can be cloned for the same reference sequence
Exported data structures		
Data structure	Data element	Element description
Clone description*	Clone sequence	Clone sequence
	Clone analysis status	Clone status (Accepted / Rejected)
Clone Sequence	Sequence text	Clone sequence text with confidence score for each base
	CDS Start	Position of CDS start on sequence
	CDS stop	Position of CDS stop on sequence
	Collection of discrepancies	Collection of all discrepancies defined for the clone
Discrepancy	Position	Position of discrepancy on clone sequence and CDS position
	Length	Length of discrepancy in bases
	Discrepancy type (see Table 2)	Discrepancy type
	Confidence score	Confidence score (see Discrepancy Finder for how score is calculated)

* New data types are added to the clone description in ACE.
