# Supplementary material for: A novel approach to sequence validating protein expression clones with automated decision making
Source: BMC Bioinformatics. 2007 Jun 13;8:198. doi: 10.1186/1471-2105-8-198 (PMC1914086; doi:10.1186/1471-2105-8-198)
Supplement: Additional file 4 — XML file with reference sequence descriptions. Example of XML file contains descriptions of reference sequences for all clones on the plate 'YGS000374-1'. [file 1471-2105-8-198-S4.pdf]

<?xml version="1.0" encoding="ISO-8859-1" ?>
<!DOCTYPE web-app *(View Source for full doctype...)*>
- <!--
XML file with reference sequence descriptions.
-->
- <!--
XML file contains descriptions of reference sequences
of all clones on the plate 'YGS000374-1'
-->
- <sequence-info>
- <refsequence>
<refsequence-id>**4476**</refsequence-id>
<refsequence-species>**2**</refsequence-species>
<refsequence-cds-start>**1**</refsequence-cds-start>
<refsequence-cds-stop>**2019**</refsequence-cds-stop>
<refsequence-chromosome>**14**</refsequence-chromosome>
<refsequence-
sequence>**ATGAAGCAGGAGCAGTCCCACGAAGGCGACTCATACAGCACGGAATTCA
TAAATCTCTTTGGCAAAGATACCGCAACACACCCTAGCAGCAACAACGGTGCTAATA
ATAATGGCATGGGGAGCACGAACTCGTTGGACCAGTTTGTGGCAACAGCCTCATCGT
CATCTTCTCTGGTGACCAGCAGCGAGAATAGGCGCCCCCTAATAGGTGACGTTACCA
ATAGGGGCAACACTAACCTATATGACCACGCTGTCACGCCAGAAATACTCTTAGAAC
AGTTGGCCTACGTGGATAACTTCATACCATCTCTGGATAACGAGTTCTCTAATGTGGA
TTGGAATGTGAATACCACCCATAATAATGCAAACAATAATGGCGCGGACACTTTCAG
CAGCATAAATGCAAATCCTTTTGACTTGGATGAACAACTAGCCATTGAGTTGAGTGC
GTTTGCCGACGATTCTTTCATCTTCCCAGACGAGGATAAGCCGAGCAATAACAATAA
CAACAGCAATAATGGTAATGACGACCATAGCAACCACGACGTATTGCATGAGGACCC
TTCTACCAATAATAGACAAAGAAATCCTCACTTCTTGACTCAAAGAAGGAATACTTTC
CTAACTTCCCAATACGACCAATCAAAGTCTCGATTTTCGTCCAAAAACAAAAGAAAT
GGCAATAACGGCGAAACAAACAACTTTGGCGACAATATGCAAAATAACCATCCTTTT
GAGCCAAACTTTATGGGAAGCCCTTCTCAGTTTCCCGCTGACGCAACTAATATGACAT
CAATCGACCATGGCGGCTTCACAAATGTTGACATTACATCAACTGAGAACAATACTA
CCGGTGACAATGGAGTGGATGCGCTATCAAATCTACTACATAGGACAACACACACAC
CGAACCGCTCCTCCCCCCTAAGCAATGTCACTTCTGCTCAAAATTCCTCTTCACAACA
ACGAAAACATTCGGAAAGCAAAGTCGATAGTAACAGCGATAATAACAGCTCCAACA
AAGCCCCCAATATAACTGTTCCTGACTATTCAATTATACCAACCTCTGTTTTAGTAAC
TCTATTACCGAGGGTCAACGTGCCCAACGGCGCATATAACTCGTTGATCAGCGCGGG
ATTTGACAATGATCAAATAGATGCTATAGCCGCAATAATGGCGTATCATCATCAAAA
AAAGATTAGGGAAAATAACAGTAATAATAATAAAAACATCAACACCAATGACAGTCA
AGAGGCACCCATTCTAAAAAACATCAACGAACTTTTAAGTGTCTTAATACCACCCTCT
CCGGCTGAAACTCGTGGGCCAACTACCTTATCAACGTCGCCTTCGTTCAATGAGCAC
GGTGTAGTAGCAGAGGCTTCTTTTCTAAGCTCCATTTTGGAACTGGGCATAAAGCAT
CCAAAAAGTAATAATATTCACAATCAACGACAACCTTCACGAAACGATCATAAAATA
TCAAGAGAGAGTGACGGTAACAATGGAAACGATAATGTCCATCATAATAACGCTGTT
ATTAAGTCAAGTACGACGCGTGGAGACGAAATTGCCAAGATACGATCCGAGCCAACT
TTAAATGCAAGTTCTTCTGATCACAAGGAAAATAGTTTAAAAAGATCACACTCCGGA
GATTTGAAAAATAAAAAAGTACCCGTCGACCGCAAGTATTCTGATAATGAAGACGAT
GAATATGACGATGCAGATTTACACGGCTTTGAAAAGAAGCAACTGATCAAGAAAGA
GTTAGGGGACGACGATGAAGATTTATTGATACAGTCGAAAAAATCTCATCAAAAAAA
AAAACTAAAGGAAAAGGAGTTAGAATCATCGATACATGAACTGACCGAAATTGCAG
CATCCTTACAAAAACGGATACATACGTTAGAAACGGAAAACAAGCTTTTAAAGAATT**

TAGTTCTGAGTAGCGGTGAAACGGAAGGAATAAAAAAAGCTGAAAGCTTAAAGAAG
CAAATTTTTGAGAAGGTTCAGAAAGAATAA</refsequence-sequence>
– <refsequence-feature>
<name_type>GENBANK_ACCESSION</name_type>
<name_value>CAA95979</name_value>
</refsequence-feature>
– <refsequence-feature>
<name_type>SGD</name_type>
<name_value>YNL103W</name_value>
<description>member of the leucine zipper family of transcriptional activators</description>
</refsequence-feature>
– <refsequence-feature>
<name_type>GI</name_type>
<name_value>1302018</name_value>
</refsequence-feature>
– <refsequence-feature>
<name_type>GENE_SYMBOL</name_type>
<name_value>met4</name_value>
</refsequence-feature>
– <refsequence-feature>
<name_type>ORF_TYPE</name_type>
<name_value>Verified</name_value>
</refsequence-feature>
– <refsequence-feature>
<name_type>ORF_STATUS</name_type>
<name_value>No change</name_value>
</refsequence-feature>
</refsequence>
– <refsequence>
<refsequence-id>4500</refsequence-id>
<refsequence-species>2</refsequence-species>
<refsequence-cds-start>1</refsequence-cds-start>
<refsequence-cds-stop>2022</refsequence-cds-stop>
<refsequence-chromosome>14</refsequence-chromosome>
<refsequence-sequence>ATGTCAACGCACTCAAACGACTACTTTTCTGCTTCTTCCGGAATGGTCTCT
GAGACATCGTCCGAGGTTTCTTCGATAAACTCTTCACAGCCTGTATCATTCTCTAAGG
CTTCTATTGCTGCTCCGGTTCCATGCTCTGATCTACACAGCACCAAGTCGAACGATGC
ATCGAGAAAATTGTCTATTAGTAGGACGTTAACTAATCGGCTCAACGACATTAAAAA
GGCTGTCGATGACGACAACTTGCAGACGGAAGAAAATTCCGCAGACGTTAATAAAAT
ATTAGAATCTAGATTTGACGTGGCCGATGCCATTAGGCTACAGCACAATGAGTCAGT
CCAGTCAAAGTTAAACATCCCAGTCACACACACCACGACTGCAGGCGCCTCGTTGTC
GGCACCATCTTCCTCTGCTTTCTCTGCTTCTTCTATTCAAAATGATACTACAGAACATA
AAGCTTCCATGGACTCCAAACTCATGAGGAATAGACTATATCCGGCTTCCACGAAAC
ACTCCGGTAAGGATCTTGAGGCCCAAGGAATAACCGAATTCGAGCCTGATGAACCGA
CTGTAAAAAAAGTATTCACCAACAAGTCTACCGGGCAGCTGGAACTGCCCCCCGACG
GTGGTTATGGCTGGGTCGTGACATTCTGTGTGTTCTTGACCATGTTTTCCACGTGGGG
CTGCAACGCATCCTTCGGTGTCGACCTTGCCTACTACTTAAACCATGATACTTACCCT
GGTGCTTCGAAGTACGATTATGCCTTAATTGCTGGCCTAACTGTCTTTCTCGGTCAAC

TCTTATCCCCCCTTGTGATGGCACTGATGAGAATAATTGGTCTGCGGACCACCATGCT
TTTTGGTGATGCTGTAATGCTTGCCGCATATCTCTTGGCCTCCTTTACTACCAAGTTAT
GGCAATTGTATGTCACCCAAGGTTTTATGGTCGGTTGTTCAATATCACTGATTTTCGT
TCCAGCAACAACCGTCTTACCAGGATGGTTCTTGAAAAAAAGAGCTGTCGCAATGGG
TGTCTCATTATTGGGTACCGGTGCTGGTGGTGTCGTTTACGGTTTGGCTACAAACAAA
ATGCTTTCTGACTTTGGAAATACCCGGTGGTGCCTTCGTATCATAGGCATATCGTGTA
GCATAAGTGTTCTAGTTGCTATTGCGCTCTTAAAAGAAAGAAACCCTACACCTGCCAT
AGGATTGAAATCGCCTCGGGCCATGTTTGAACAGCTCAAAGCAATGTTTTCATTAAA
GGTTATAACTAAGCCATTTGTGGTACTTATTGCATTATGGTTCATGTTCGCATTATTT
GCCTACAATATGATGGTTTTTACTTTATCTTCATACGCAATCTCGAAAGGATTATCAT
CGCACGACGCTTCCACATTGACTGCCATTTTGAACGGCTCGCAATCCATCGGAAGAC
CTCTGATGGGTTTAGCGGGAGATAAGTTTGGTAGGGCAAACGTAACGATCGTATTAA
CCACTTTGTTAACAATATATATGTTTGCGTTCTGGATCCCCGCTCATACGTTTGTTCA
ACTCATCTTTTTTTCAATTCTAGTTGGCTCATGCGTTGGTGTCGCCAACGTCATGAAT
ACCGTTCTGATTGCCGATATGGTTAAACCAGAAGAGTTTTTGCCCGCTTGGGCCTTCG
TCAACTACTGTGGTGCGCCTTTCTTATTGGTTTGTGAGGTGATTGCCCAGGCATTGAC
GGTGGAGAAAGATAAGAGCAATCCTTACTTACATGCACAAATTTTTTGCGGTTGCTG
CTTTATTGCCGCACTAATTTTAATTTCTATCCTTCGTGAATATTCTATCAGGATGAAAT
TAACGGAAAGACAAGCTATGACAAACGAGAAGTTAAAAGAATGGAAGGCAAGCGAA
TACGATACCGATTCTGCCGATGAAGATTGGGGTAAATTAAAAGAAAGAAAGACTAAA
TATGACCTTCTTTTAGGTCCGGGCATTAAAAAATACTTCCTAAGAATGGTATATCCAA
TGAAGGTCTAG</refsequence-sequence>
- <refsequence-feature>
<name_type>GENBANK_ACCESSION</name_type>
<name_value>CAA96006</name_value>
</refsequence-feature>
- <refsequence-feature>
<name_type>SGD</name_type>
<name_value>YNL125C</name_value>
<description>Putative monocarboxylate permease</description>
</refsequence-feature>
- <refsequence-feature>
<name_type>GI</name_type>
<name_value>1302058</name_value>
</refsequence-feature>
- <refsequence-feature>
<name_type>GENE_SYMBOL</name_type>
<name_value>ESBP6</name_value>
</refsequence-feature>
- <refsequence-feature>
<name_type>ORF_TYPE</name_type>
<name_value>Verified</name_value>
</refsequence-feature>
- <refsequence-feature>
<name_type>ORF_STATUS</name_type>
<name_value>No change</name_value>
</refsequence-feature>
</refsequence>
- <refsequence>
<refsequence-id>4853</refsequence-id>
<refsequence-species>2</refsequence-species>

<refsequence-cds-start>1</refsequence-cds-start>
<refsequence-cds-stop>2031</refsequence-cds-stop>
<refsequence-chromosome>15</refsequence-chromosome>
<refsequence-
sequence>ATGCTCAGAAGACACGGATTATTCTGGTTAAAGACATGTCCCCGTCTAAA
TGTTCTTCTAAATCAATCCATACCGATACCTCATTTGCTTCATAGCCGTGATATTTGCC
AGCAAAGATGGTATGCAAAAGGGAAAAGAAGGAACCAGATTTCCAAGAAAGAGTTA
AAGCCTCTCAATTTTTCTATTCCGAATTATATTTCTGTGAATAAATTGGCCAATTTGTT
AAATTGTCGTGTGGAAAGATTAATCAAAGATTTGACAGCATTAGGTTTCGAAAATAT
TACAACCACGTATATATTATCAAAAGAATACGTCGAATTGATTTTACAAGAATACAAC
TTTGCCCTTCCGAATCTGTCAACGTCTACAAATTTAGATAATGTTTATGATGAGCTAA
AATCTCCTGTAAACCCAAAATTATTAACCAAAAGAGCCCCCGTGGTAACTATAATGG
GCCATGTTGATCATGGTAAGACCACTATCATAGACTATCTAAGGAAATCTTCTGTTGT
GGCTCAAGAACATGGTGGAATCACGCAACACATTGGTGCCTTTCAAATAACAGCTCC
GAAGTCTGGGAAAAAGATCACATTTTTAGACACCCCTGGCCATGCTGCCTTTTTGAA
AATGAGAGAAAGAGGTGCAAATATTACTGATATTATTGTTTTGGTGGTATCTGTAGA
AGATTCTCTGATGCCCCAGACATTAGAAGCCATAAAACATGCCAAAAATTCAGGAAA
TGAAATGATAATTGCAATAACGAAAATAGATAGAATTCCACAGCCAAAAGAACGTGA
AAAAAAAATTGAAAAAGTTATAAATGATTTGATTGTTCAAGGAATACCAGTAGAGAA
AATTGGCGGAGACGTTCAAGTGATCCCGATAAGTGCCAAAACTGGTGAGAACATGG
ACCTTTTAGAGGAATCAATAGTCCTATTAAGTGAAGTAATGGATATAAGAGCTGAAA
ACTCCCCTAAAACTATCGCAGAGGGTTGGATTATCGAAAGTCAAGTTAAAAAACAGG
TCGGAAATGTGGCGACCGTCTTGGTTAAGAAGGGCACTTTACAAAAAGGGAAAATTC
TAATTTGTGGTAATACATTCTGCAAGATCAAAAATTTAATTGATGATAAGGGCATAC
CGATTCTTAAGGCAACACCATCATATGCTACAGAGGTTCTTGGTTGGAAAGATGTAC
CCCATGTTGGAGATGAGGTCATTCAGGTAAAGAGCGAAGCGATCGCTAAAAAGTTCA
TCAGTAAAAGGCAAGATCTTATCGAAGTTCAAAAAAACTCTAGTATCGTTGAAAAAT
TAAATGAAGAAAGGGCCCTTGCCAAAGAGCAACACCTAAATAAGGAACTGGAACAT
GAGAATACTGTCCAGGAACACGAACAAAATACTGGTCCTAAACTTATTAATTACATC
ATCAAATGTGATGTCTCTGGCTCAGCAGAAGCGGTTTCAGAAAGCATTTCCTCTCTAG
GAAATGACGAAGTGAGATGTAATGTTATCTCTTCTTCTGTTGGTATACCTACGGAAA
GTGACTTAAAGATGGCGCAAATAACGGAGAGTACAATTCTATGCTTCAATTTGGGGA
ATTTGCCGAGTGAGGTGATCAACAATCGTGCTGGCATCAAAATAAAACAGTACAATG
TTATCTATAAGCTAATTGAAGACGTCACTGAAACACTAACTGAAAATCTAAAGCCCA
TCTTTGAAAAAAAGATCGTTTCAACTGTTGACGTTCGTGAAACTTTTGACTTTAGGTT
AAAGAAAAAAATTATTAGGATTGCAGGTTGCAAAGTAAACAACGGTGTCATCAAAAA
GAATTCATTGGTGCAGGTAGTTAGAGGTCCTAACGAAGATGTAATTTTTGATGGGAA
AATCTCTACTTTGAAGCACAATAAAGATGACGTAGCAGAAGTTTCAAAAGGGCATGA
ATGTGGAATCACTTTTGAGAGTGGATTCGAGGGTTTCAAACCAGGTGATAAGATCCT
AGTTTATGAAAACGTGCGAGTTCCTCGTTACTTGTAA</refsequence-sequence>
- <refsequence-feature>
<name_type>GENBANK_ACCESSION</name_type>
<name_value>CAA99023</name_value>
</refsequence-feature>
- <refsequence-feature>
<name_type>SGD</name_type>
<name_value>YOL023W</name_value>
<description>mitochondrial initiation factor 2</description>
</refsequence-feature>
- <refsequence-feature>
<name_type>GI</name_type>
<name_value>1419805</name_value>

</refsequence-feature>
- <refsequence-feature>
<name_type>**GENE_SYMBOL**</name_type>
<name_value>**IFM1**</name_value>
</refsequence-feature>
- <refsequence-feature>
<name_type>**ORF_TYPE**</name_type>
<name_value>**Verified**</name_value>
</refsequence-feature>
- <refsequence-feature>
<name_type>**ORF_STATUS**</name_type>
<name_value>**No change**</name_value>
</refsequence-feature>
</refsequence>
- <refsequence>
<refsequence-id>**4063**</refsequence-id>
<refsequence-species>**2**</refsequence-species>
<refsequence-cds-start>**1**</refsequence-cds-start>
<refsequence-cds-stop>**2055**</refsequence-cds-stop>
<refsequence-chromosome>**13**</refsequence-chromosome>
<refsequence-
sequence>**ATGGACACCAGCAAGAAAGATACTACTAGGTCGCCCTCACATTCCAACAG
TTCTTCTCCTTCATCTTCTTCTCTCTCTTCATCTTCTTCCAAAGAGAAAAAACGTCCTA
AGAGACTATCCTCCCAGAATGTAAATTACGATTTGAAAAGGAGAAAGATCATCACTT
CTGAAGGTATAGAAAGATCATTCAAGAACGAGCATAGCAATCTTGCAGTTGAAGACA
ATATCCCGGAAGAAGAACCCAAGGAACTTCTTGAAAAGGACTCCAAGGGTAATATCA
TCAAACTTAATGAGCCATCCACCATCTCGGAAGATTCGAAGGTATCTGTCACTGGATT
GCCGTTAAATAAAGGTCCTTCTGAGAAAATCAAGCGAGAGTCTCTTTGGAATTATAG
AAAAAATTTGGGAGGCCAATCGAACAACTCAGAAATGACGCTGGTCCCCAGTAAAA
GATTTACTCAGGTGCCGAAAAATTTTCAGGATTTGAACAGAAATGATTTGAAAACGT
TTTTAACTGAGAACATGACTGAAGAGAGCAATATACGGTCAACTATTGGTTGGAATG
GCGATATAATAAACAGAACTCGTGACCGTGAACCTGAAAGCGACCGCGATAATAAAA
AGCTATCAAATATTAGAACCAAAATAATACTTTCAACGAATGCTACCTATGATTCAAA
AAGTAAACTATTTGGCCAAAATTCCATCAAGTCAACATCGAATGCGAGTGAAAAAAT
TTTCAGGGACAAGAACAATTCAACCATAGATTTTGAGAATGAAGATTTTTGTTCGGC
TTGTAATCAGTCAGGTTCTTTTTTATGCTGTGATACATGCCCCAAATCCTTTCACTTTC
TTTGCTTAGACCCACCAATTGATCCGAACAACCTGCCTAAAGGTGATTGGCATTGTAA
TGAATGTAAGTTTAAAATCTTCATAAATAACTCAATGGCAACTTTAAAAAAAATTGA
ATCCAACTTCATTAAGCAAAATAACAACGTCAAGATTTTTGCTAAATTACTTTTCAAT
ATCGATTCCCACAACCCAAAACAGTTCCAGCTACCAAATTATATAAAAGAAACCTTTC
CTGCTGTGAAAACGGGTTCGAGAGGACAATACTCTGATGAGAATGATAAGATTCCAT
TAACTGATAGACAATTGTTTAACACTTCTTATGGCCAAAGCATAACTAAATTGGACTC
TTACAACCCGGATACACATATAGATTCAAATTCAGGTAAGTTTTTAATTTGTTACAAA
TGCAATCAAACCAGACTAGGTTCATGGTCTCATCCAGAAAATTCAAGATTAATAATG
ACTTGTGATTATTGTCAGACTCCATGGCATTTGGATTGTGTACCGAGGGCTTCTTTCA
AGAACTTAGGTTCAAAATGGAAATGTCCTCTACATTCCCCAACTAAAGTTTACAAAA
AGATACACCATTGTCAAGAAGATAATAGCGTAAATTATAAGGTGTGGAAAAAACAAC
GATTGATAAACAAGAAAAACCAACTTTATTATGAACCTTTACAAAAAATAGGCTACC
AAAATAACGGGAACATTCAAATCATACCAACAACTAGTCATACAGATTATGATTTCA
ATCAAGATTTCAAAATTACACAAATAGATGAAAATTCCATTAAATATGATTTTTTTGA
TAAAATTTACAAATCAAAAATGGTTCAAAAAAGAAAACTCTTTCAATTTCAAGAAAG**

TTTAATAGATAAGTTAGTATCAAATGGATCCCAAAATGGCAATAGCGAGGATAATAT
GGTCAAAGACATAGCCTCTTTAATATATTTCCAAGTTAGCAACAACGACAAGAGTAG
TAACAATAAATCGGCTTCCAAAAGCAATAACTTAAGAAAATTATGGGATTTGAAAGA
ACTGACTAACGTAGTCGTGCCGAACGAACTAGATTCTATACAATTCAATGACTTTTCC
AGCGATGAAATAAAGCATCTGTTATATTTAAAGAAGATTATCGAATCCAAACCAAAG
GAAGAACTGTTAAAATTTTTAAACATAGAAAATCCCGAAAACCAATCCGAATAG</re
fsequence-sequence>
– <refsequence-feature>
<name_type>GI</name_type>
<name_value>763022</name_value>
</refsequence-feature>
– <refsequence-feature>
<name_type>SGD</name_type>
<name_value>YMR075W</name_value>
</refsequence-feature>
– <refsequence-feature>
<name_type>GENBANK_ACCESSION</name_type>
<name_value>CAA88800</name_value>
</refsequence-feature>
– <refsequence-feature>
<name_type>ORF_TYPE</name_type>
<name_value>Uncharacterized</name_value>
</refsequence-feature>
– <refsequence-feature>
<name_type>ORF_STATUS</name_type>
<name_value>No change</name_value>
</refsequence-feature>
</refsequence>
– <refsequence>
<refsequence-id>4839</refsequence-id>
<refsequence-species>2</refsequence-species>
<refsequence-cds-start>1</refsequence-cds-start>
<refsequence-cds-stop>2061</refsequence-cds-stop>
<refsequence-chromosome>15</refsequence-chromosome>
<refsequence-
sequence>ATGATACGTCCATTATGTTCAAAAATTATTATCAGTTACATATTCGCAATT
TCTCAGTTTCTACTGGCCGCTAATGCGTGGTCGCCCACAGATAGTTATGTTCCTGGCA
CCGTGTCGTGTCCCGATGACATAAATCTGGTAAGAGAGGCTACGTCTATATCTCAGA
ATGAGAGCGCATGGTTGGAAAAGAGGAATAAAGTCACTAGTGTAGCTTTAAAAGAT
TTCTTGACTAGGGCTACTGCAAATTTTTCAGATAGCTCAGAAGTTTTGTCGAAGCTAT
TTAATGATGGCAACAGCGAAAACCTGCCGAAAATTGCTGTCGCCGTTTCAGGTGGGG
GCTATCGGTCCATGCTAACAGGTGCGGGTGTTCTAGCAGCAATGGATAACAGAACTG
AAGGTGCTTATGAGCATGGGCTGGGTGGACTTTTACAAAGCACAACATATTTATCTG
GTGCCTCGGGCGGCAACTGGCTAGTTGGTACATTAGCCTTGAACAATTGGACATCCG
TGCAAGACATTCTTAATAATATGCAGAACGACGATTCTATTTGGGATTTGTCAGATTC
TATTGTTACCCCCGGCGGCATTAATATATTCAAAACAGCCAAAAGGTGGGATCATAT
CTCTAATGCTGTCGAATCTAAGCAGAACGCCGATTACAATACTTCTTTGGCCGATATT
TGGGGCAGAGCCTTGGCGTATAATTTTTTCCCTTCTCTAAATAGAGGGGGTATAGGC
CTGACTTGGTCTTCCATTAGGGATTTCCCAGTGTTTCAAAATGCTGAAATGCCTTTTC
CAATTTCTGTTGCGGACGGTAGGTATCCTGGAACAAAAGTCATCAATTTGAACGCAA
CGGTTTTTGAATTTAATCCCTTTGAAATGGGATCCTGGGATCCCTCTTTGAACTCTTTT

GCCAACGTTAAGTACCTTGGAACGAATGTCTCCAATGGTGTACCATTGGAAAGGGGA
AAATGTACCGCAGGCTTTGATAATGCAGGTTTTATCATGGGTACTTCCTCCACCTTAT
TTAACCAGTTTCTTTTGAGAATAAACTCCACTCACTTACCTAGTTTCATCACAAGATT
AGCAAGACATTTTCTAAAGGATTTATCTCAAGATTTTAATGATATCGCTGTTTACAGT
CCTAATCCATTTAAAGACACGAAGTTTTTGGATAGTGACTATACGACAAGTATTGTTG
ATTCAGACAGCCTATTCTTAGTTGACGGCGGTGAAGACGACGAAAATGTTCCTGTGT
TACCGCTTATACAAAAGGAACGTGATGTGGACATTATTTTTGCGGTAGATAATTCTG
CCGATATGAGATTGGCTTGGCCTGACGGTTCTTCTTTAGTCCATACCTATGAGCGTCA
ATTCGTTAAGCAAGGTCAAGGTATGTCCTTCCCATATGTTCCAGATACGAATACATTC
GTTAATTTGGGTTTGAACAAGAAACCAACCTTCTTCGGTTGCGATGCTAATAATTTGA
CCGATCTTCAATATATTCCACCTCTAGTTGTTTATCTGCCAAATGCAGAATACTCTTTC
AATAGTAATCAGAGCGCGTTCAAGCTATCATATTCCGAATCCCAAAGAAGAAGTATG
ATTCAAAACGGGTTTGAAATTGCAACTAGGAACAACTTCACTGATGATCCTGAATTC
ATGGGCTGCGTCGGCTGCGCCATCATTAGACGTAAGCAACAAGCTTTGAATATTACT
TTGCCTCCGGAATGTGAAACCTGTTTTAAAAATTATTGCTGGAATGGTACCCTGGATA
CTACCCCATTACCAGATGTTGAAAAGGATGTTCACCACTCCTTTATCAACGTCAATAG
CTTCAACAGCTCCATAGGACAAGAAGAAAGTCTTTACGCCGGATCTTCAGCTTCCCA
GTCATCGTCTTCATCATCGTCTTCATCTTCGTCTTCGGAAATTCCTTCGGCTACTGCAA
CCCTTGAGAAAAAGGCAGCTACTAATTCTGGATCACATTTATCTGGCATAAGTGTAA
AGTTCTCTGCTATGATTATGCTTACTTTATTGATGTTTACCGGAGCAGTATAA</refse
quence-sequence>
- <refsequence-feature>
<name_type>GENBANK_ACCESSION</name_type>
<name_value>CAA99010</name_value>
<url>http://www3.ncbi.nlm.nih.gov/htbin-
post/Entrez/query?db=p&form=6&uid=CAA99010&Dopt=g</url>
</refsequence-feature>
- <refsequence-feature>
<name_type>SGD</name_type>
<name_value>YOL011W</name_value>
<description>phospholipase B\/lysophospholipase</description>
<url>http://genome-www.stanford.edu/cgi-
bin/dbrun/SacchDB?find+Sequence+%22YOL011W%22</url>
</refsequence-feature>
- <refsequence-feature>
<name_type>GI</name_type>
<name_value>1419781</name_value>
</refsequence-feature>
- <refsequence-feature>
<name_type>GENE_SYMBOL</name_type>
<name_value>PLB3</name_value>
</refsequence-feature>
- <refsequence-feature>
<name_type>ORF_TYPE</name_type>
<name_value>Verified</name_value>
</refsequence-feature>
- <refsequence-feature>
<name_type>ORF_STATUS</name_type>
<name_value>No change</name_value>
</refsequence-feature>
</refsequence>

- <refsequence>
<refsequence-id>770</refsequence-id>
<refsequence-species>2</refsequence-species>
<refsequence-cds-start>1</refsequence-cds-start>
<refsequence-cds-stop>2109</refsequence-cds-stop>
<refsequence-chromosome>15</refsequence-chromosome>
<refsequence-
sequence>ATGGGTATCCAAGGTCTTCTTCCTCAGTTAAAGCCCATACAGAATCCAGT
ATCACTACGTAGGTATGAAGGAGAAGTGTTAGCCATTGATGGCTATGCATGGCTACA
TAGAGCAGCCTGCTCTTGTGCTTATGAACTTGCAATGGGAAAACCAACTGATAAGTA
CCTGCAGTTTTTCATAAAAAGATTTAGTTTATTGAAAACCTTTAAAGTTGAACCGTAT
TTGGTCTTCGATGGTGATGCCATTCCAGTTAAAAAGTCTACTGAATCTAAAAGAAGG
GATAAGAGAAAAGAAAACAAAGCCATAGCTGAAAGACTGTGGGCCTGTGGCGAAAA
GAAAAATGCTATGGACTATTTTCAAAAATGTGTCGACATAACGCCTGAAATGGCAAA
ATGTATCATATGCTACTGTAAGCTAAACGGTATTCGGTACATAGTGGCTCCGTTTGAG
GCTGACTCTCAAATGGTATATTTAGAACAGAAAAACATTGTGCAAGGAATAATATCC
GAAGATTCTGACCTCCTCGTCTTCGGATGTCGACGTCTCATTACGAAGCTGAATGATT
ATGGAGAATGTTTAGAAATATGTCGCGATAATTTTATTAAACTGCCTAAAAAGTTTCC
GTTGGGATCCTTAACTAATGAAGAAATCATAACAATGGTTTGTTTATCCGGTTGTGAC
TATACAAATGGAATTCCCAAGGTTGGCCTGATTACCGCAATGAAATTAGTTAGAAGA
TTCAATACTATTGAAAGGATAATTCTGAGTATACAGCGGGAGGGAAAACTGATGATA
CCAGACACATACATTAATGAATATGAAGCTGCAGTTTTAGCATTTCAATTCCAAAGG
GTATTTTGCCCTATTCGGAAGAAAATAGTGAGCTTGAATGAAATTCCGCTGTACTTG
AAAGATACCGAAAGTAAAAGAAAAAGACTCTACGCGTGCATTGGTTTTGTCATACAC
AGAGAAACTCAGAAAAAGCAAATTGTTCATTTCGACGACGATATAGATCACCACTTG
CATTTAAAAATTGCTCAAGGTGACTTGAATCCATATGATTTTCACCAACCTCTAGCCA
ACAGAGAGCACAAATTACAGCTGGCATCCAAGTCAAATATAGAGTTCGGGAAAACTA
ATACTACTAATTCTGAAGCCAAAGTCAAACCAATAGAATCGTTCTTCCAAAAAATGA
CAAAATTGGATCATAACCCAAAAGTTGCAAATAACATCCATAGTCTAAGACAAGCGG
AAGACAAACTGACAATGGCAATTAAGCGTAGGAAATTAAGTAATGCCAATGTAGTCC
AAGAAACGTTGAAGGATACAAGAAGCAAATTTTTTAATAAACCCTCCATGACTGTTG
TGGAAAACTTCAAAGAAAAAGGCGACAGCATACAGGATTTTAAAGAGGACACAAAC
TCACAATCTTTGGAAGAGCCTGTTTCCGAGTCTCAACTATCTACACAAATACCTAGTT
CTTTTATTACAACCAATTTAGAGGATGACGACAACCTGAGTGAAGAGGTTTCTGAAG
TTGTCAGTGACATTGAAGAGGACCGAAAGAATTCTGAAGGAAAGACTATCGGTAAC
GAAATCTATAATACAGATGATGACGGTGATGGCGATACTAGCGAGGATTATAGCGA
AACTGCGGAATCAAGAGTTCCCACGAGCAGCACCACCTCATTTCCCGGGTCATCCCA
GAGGAGTATATCGGGGTGCACAAAAGTTTTACAAAAGTTTAGATATTCATCTTCATT
CAGCGGAGTTAATGCAAATAGACAACCACTATTTCCTCGGCACGTTAATCAAAAAAG
TAGAGGAATGGTATACGTCAATCAAAATAGAGATGATGATTGCGACGATAACGATG
GTAAAAATCAAATCACGCAAAGGCCATCACTGCGAAAAAGCCTTATTGGTGCCAGGT
CACAGAGAATTGTGATTGACATGAAAAGCGTAGATGAACGAAAATCATTCAATTCAT
CACCAATTTTGCATGAGGAAAGTAAGAAAAGGGACATCGAAACTACTAAGTCAAGCC
AAGCTCGGCCGGCAGTCAGATCTATCTCCTTGCTTTCCCAATTTGTTTATAAAGGTAA
ATAA</refsequence-sequence>
- <refsequence-feature>
<name_type>SGD</name_type>
<name_value>YOR033C</name_value>
<description>exonuclease</description>
<url>http://genome-www.stanford.edu/cgi-
bin/dbrun/SacchDB?find+Sequence+%22YOR033C%22</url>
</refsequence-feature>

- <refsequence-feature>
<name_type>**GENE_SYMBOL**</name_type>
<name_value>**DHS1**</name_value>
</refsequence-feature>
- <refsequence-feature>
<name_type>**GI**</name_type>
<name_value>**1420150**</name_value>
</refsequence-feature>
- <refsequence-feature>
<name_type>**GENBANK_ACCESSION**</name_type>
<name_value>**CAA99223**</name_value>
<url>**http://www3.ncbi.nlm.nih.gov/htbin-post/Entrez/query?db=p&form=6&uid=CAA99223&Dopt=g**</url>
</refsequence-feature>
- <refsequence-feature>
<name_type>**ORF_TYPE**</name_type>
<name_value>**Verified**</name_value>
</refsequence-feature>
- <refsequence-feature>
<name_type>**ORF_STATUS**</name_type>
<name_value>**No change**</name_value>
</refsequence-feature>
</refsequence>
- <refsequence>
<refsequence-id>**4893**</refsequence-id>
<refsequence-species>**2**</refsequence-species>
<refsequence-cds-start>**1**</refsequence-cds-start>
<refsequence-cds-stop>**2136**</refsequence-cds-stop>
<refsequence-chromosome>**15**</refsequence-chromosome>
<refsequence-sequence>**ATGAGCCACTTTTTCGCCGATCATGATGCTCCTCTGAGCATGCTTTCTGTT
AAAACAGAATACTTTCCTCAATTGACTGATAAGGAACAAAAATATGCGCATTTCATG
TCAAAGGCCTCCCATGCGGGTTCAAGGGTTGTAATGAGACAAGTTTCTCATGAGAGT
GAGCCAATTTTTGACCTAATCCTTGCCATTCATTCAAAGCTAAACGGCAAGTACCCAG
AGGACGATATTACGCAGAAGCAGCAAACGGGTTTGTATTTGGAATACGTTTCTCAAT
TCTTATCTAATTTGGGTAATTTCAAATCGTTTGGTGACACTAAGTTTATTCCTCGTTGT
GAGGTAAAATTCTTCAAACAGCTTTTGGAGCTGGCCAAGATTAATCCGTGTTCTTCTC
CGCTCACTTTATCTCCTGTTGACGTTAACCATGAATTCACATCTCATCATCTTTTTTCC
ACCATCAATGAGCTAATTGATATTGGTATTTACCATGTGGAAGAGAAGGCGGCTCTC
TTAGGGTTTCCCTCTCAAGGTTATACTTCAGCCTATTATCTGGGTTTACCTGTGACAC
CTGAAGATATGGCTCTTTTGAAAGAGCAGTTGTTTGCTGAACTTGCCATCTTGCCTGA
AAACACAAGAATCAACAAAGTTGGTGAAAACAGTTTCCAAATCTGGGTTGCCTCTGA
GAATGTGAAAAACCAGATAACAGAAACCTACCCCAGTGGACAGATCACATTATCCAA
TGCTGTAACCAAAGTAGAATTCATTTTTGGTGATCATTCACGTGAAATGCGTTTAGTA
GCATCGTATTTAAAGGAAGCTCAAAAATTCGCGGCTAATGATACTCAAAAAGCAATG
CTTCAGGAATACATCAACCACTTTGTCACTGGCTCTTCTCAAGCACATAAAGAAGCAC
AAAAACTTTGGGTCAAAGATATATCTCCCGTCATTGAAACAAATATCGGTTTTATCGA
AACATATAGAGAACCCTCGGGCATAATTGGAGAATTTGAATCGTTGGTTGCAATTCA
AAACAAAGAACGTACTGCTAAATTTTCCAGCTTGGTTAACAACGCAGAAGAATTCAT
TTCCTTACTACCATGGTCTAAAGATTACGAAAAACCGATTTTCAATCCACCAGATTTC
ACCTCTCTAGAAGTATTAACGTTTACTGGATCGGGTATACCAGCGGGCATCAATATTC**

CAAACTATGATGATGTTCGGCTTAAAATTGGGTTCAAGAATGTTTCTTTGGGGAATA
TCTTAAGCGCGGCTGCCAAAAGCTCATCCAAGCATCCGCCAAGTTTTATATCGCAAG
AAGATCGCCCAATTTTTGAAAAATATCAAAGTGATTCTTTTGAAGTCCAAGTAGGCAT
CCATGAATTATTAGGACATGGTTCAGGAAAGTTGTTGACAGAATTTACAGACGGCTT
TAATTTTGATAAGGAAAACCCTCCTTTAGGTTTGGATGGGAAACCGGTGAGCACATA
CTACAAAGTTGGTGAAACTTGGGGTTCCAAATTTGGACAGTTAGCTGGCCCATTTGA
AGAATGTCGTGCGGAAGTAATTGCCATGTTTTTGCTTACTAATAAGAAGATTCTTGAT
ATTTTTGGTTTCCATGATGTCGAATCTCAAGATAAAGTGATCTACGCTGGATATCTAC
AAATGGCCCGTGCGGGTCTCCTAGCTTTAGAATACTGGAATCCAAAAACTGGTAAGT
GGGGACAACCACACATGCAAGCAAGATTTTCTATCATGAAAACATTTATGAAGCACT
CTACAGATAAGAATTTCTTAAAGTTGGAGATGAACAGCACGAATGATGATTTTGCCA
TCAAGTTGGATAAATCTCTCATTAAAACAGCGGGACATGAATGTGTGAAAGACTATT
TAAAGCATTTGCATGTTTACAAATGTTCAGGCGATGTGGAACAGGGAAGTAAGTACT
TTATTGATAGATCAACGGTGACACCGGATTTGGCGTCTTTAAGAGACATCGTCTTATC
TAAGAGATTGCCAAGGAGACAATTCATACAATCGAATTCTTATATTGACGACAATAA
CAAGGTAACCCTGAAAGAATATGATGAAACCCCACAGGGTATGCTCCAATCTTTCCT
TGATAGAGAATTATGA</refsequence-sequence>
- <refsequence-feature>
<name_type>GENBANK_ACCESSION</name_type>
<name_value>CAA99066</name_value>
</refsequence-feature>
- <refsequence-feature>
<name_type>SGD</name_type>
<name_value>YOL057W</name_value>
</refsequence-feature>
- <refsequence-feature>
<name_type>GI</name_type>
<name_value>1419869</name_value>
</refsequence-feature>
- <refsequence-feature>
<name_type>ORF_TYPE</name_type>
<name_value>Uncharacterized</name_value>
</refsequence-feature>
- <refsequence-feature>
<name_type>ORF_STATUS</name_type>
<name_value>No change</name_value>
</refsequence-feature>
</refsequence>
- <refsequence>
<refsequence-id>4474</refsequence-id>
<refsequence-species>2</refsequence-species>
<refsequence-cds-start>1</refsequence-cds-start>
<refsequence-cds-stop>2142</refsequence-cds-stop>
<refsequence-chromosome>14</refsequence-chromosome>
<refsequence-
sequence>ATGGTCACTAACAATGGAGATGGTGAGCACCTTGGGATCCGAAGAAACG
GAAATTTAAGGCATCCTTCCAATAATATGAAAATTCCCAGAAGGGCTCAATCAACAG
TTCTCAATTCAAACCCATTTTATAGTAGGAAGTATTCTATGTCTACCTTAACGCCGAG
GGATATATGTCGAAGCGTTGATTCTAGAGTATTTGTGGATATGTCTTCTCCCAATTTT
CAGACTCTGGAGGATCCCCACAGAGATGAGATTATAAACAGTGTACGGCTTAACTAC
TTGAACAGTAGCAAAAGAAGTTCCGTCTCTCATGGAAATGAAGCAATCCCTAGGGTT

AATCCGACTAAGAATTCTTCAGCAAGTACCATAGCTGCAGCAAATGTTGATAGTGAT
GACGATGAGACTAACCTGAGTAGCGCTGGAGGCGACATAACGCATGATATTTACAAG
TTGGTTAAAGCAGAGGATCCTAAACGGCTCAGACGCCCTCGGTCCATGGAAAATGTA
ACACCTAAAATTGAGCACCATACTAAATTGTCATCAGCAAGTGGTTTAAATGTCCCCG
GTGGCTTCAGAAGGGAATTTATTGTCAACAAAAAAAGGCAAGAACATCAATTAAATG
ATTCCGCTAGCTCAGACTTCACTTCGCATGAAAGCGATTCGATTAACCAGTCATCTCC
ATCTTCTAACCAAGATATCGATAAGGTCCCCTTTTTAACAAGAAACTTTTTGGAATTT
CTATACGTGTTTGGACATTTTGCTGGTGAATCCTTCGAGGATGATTTTATCCCTGATA
GCTCAAACATGATGATACGCGGAGAAGATGAAAGGAGTGCATTGCTGTCTCGGCCTG
ACCATATGAAAGTATTACCTTCAGCCAAAGGTACGACTTCGACAAAGAAGGTTTTTTT
GATATTGCTTAAATCTTTCATTGGAACTGGCGTTTTATTTTTACCTAATGCCTTTCATA
ATGGTGGTTTGTTTTTCTCAGTAAGTATGCTGGCCTTTTTTGGAATTTATTCATACTG
GTGTTACTACATATTGGTGCAGGCCAAATCTTCATGCGGTGTATCCTCCTTTGGTGAT
ATTGGTTTAAAACTGTATGGACCTTGGATGAGAATAATAATTCTTTTCTCCCTAGTAA
TAACTCAAGTTGGATTCTCTGGAGCTTATATGATATTTACTGCAAAAAATTTACAAGC
TTTTCTTGATAACGTATTCCATGTGGGCGTCCTACCTTTATCTTACCTGATGGTCTTTC
AAACGATCATTTTTATTCCACTTTCGTTTATTAGAAACATTTCGAAATTGTCACTACCA
TCTTTACTAGCAAATTTTTTCATCATGGCTGGTTTAGTCATTGTCATTATTTTTACAGC
CAAAAGGTTGTTTTTTGACCTGATGGGGACCCCAGCAATGGGTGTCGTCTATGGCTT
AAATGCAGACCGTTGGACGCTGTTTATCGGGACGGCAATATTTGCTTTCGAAGGAAT
CGGGTTAATTATTCCTGTTCAAGACTCAATGAGAAATCCTGAAAAATTTCCTTTAGTA
CTTGCTTTGGTGATTCTAACGGCAACCATACTTTTCATTTCCATAGCTACATTGGGAT
ATTTGGCATACGGCTCAAATGTTCAAACGGTTATACTGTTGAACTTACCTCAAAGTAA
CATTTTTGTAAACTTAATCCAGCTGTTCTATTCCATCGCAATTATGTTGTCCACACCTT
TGCAGTTGTTTCCAGCTATTAAAATTATTGAAAATAAGTTTTTTCCAAAGTTCACTAA
AATATATGTCAAGCACGATGATCTAACAACAAGAGTCGAATTACGTCCTAATTCAGG
AAAGCTGAACTGGAAAATTAAATGGCTGAAGAACTTTATTCGTTCTATAATCGTAAT
CATTGTTGTATCAATTGCTTACTTTGGGTCTGATAATTTAGACAAGTTCGTTTCAGTT
ATAGGATCCCTCGCCTGTATTCCTTTGGTATATATATACCCATCGATGCTGCATTTAC
GGGGCAATAGCCTTCCAGAGACTAAAGGGGAATTTTGGAGATTCAAACCAATGTTAG
ATACCATTTTAATTTTCTTCGGTATTGCAAGCATGCTTTATACGTCTTACCAGAGTAT
ATTCGGTGTCTAG</refsequence-sequence>
- <refsequence-feature>
<name_type>GENBANK_ACCESSION</name_type>
<name_value>CAA95977</name_value>
</refsequence-feature>
- <refsequence-feature>
<name_type>SGD</name_type>
<name_value>YNL101W</name_value>
</refsequence-feature>
- <refsequence-feature>
<name_type>GI</name_type>
<name_value>1302014</name_value>
</refsequence-feature>
- <refsequence-feature>
<name_type>ORF_TYPE</name_type>
<name_value>Verified</name_value>
</refsequence-feature>
- <refsequence-feature>
<name_type>ORF_STATUS</name_type>
<name_value>No change</name_value>

</refsequence-feature>
</refsequence>
- <refsequence>
<refsequence-id>**4847**</refsequence-id>
<refsequence-species>**2**</refsequence-species>
<refsequence-cds-start>**1**</refsequence-cds-start>
<refsequence-cds-stop>**2145**</refsequence-cds-stop>
<refsequence-chromosome>**15**</refsequence-chromosome>
<refsequence-
sequence>**ATGACAGAAATTATTGATTTAGACTTGGTCGATGACTTTATCAAGAAACC
TATGGTCAAACAACAGAAAAACCAGTCTTCAAAGCCTAGGGTAAAAGAAGGGGTC
AGTTGACCTTTGATGATTTCAGAAACATCAAAATTGTAGAAGAACCTGTTGTACTTTC
TCACAATAGTTCAATTGACGAATCACTGGATGCTGCTACCCAAAATACTAAAAAGCG
GGAGAAATATGAAGGTACCTGTGATGAAGAAATGAAAACTAAAGAAATGGAGGCAA
ATATGGCAAGCAAATATTCTAATATCAAAACACATTCGAACGACACTAACAAGGTAG
AGTCGATCAGTGAACACACTACTTCTAATAATAGACCATTGAATACATTAAACTGGTC
ACCAAATATTCCTTTACGATATTCTGACTTTGCAAAGTTTATGAGTGATGAGACGGTA
ACGGAAAGCAATTGGGCGCCTCCCCTTTGCACCCCTCTCCCATATGCAGGCGATGTTA
TGAAGATCCTTTCTTTTATCGTCAAATTTAAATGGGTGTTCAGCGACGATCTACTGAA
CCTGTCATTTCAAGATGTGGAGATAGGACTAGAATTAAAGGTGGCTGGCCATTCTGC
GAAGAATATTCGAATTTGTCAGGATAAAATGAATCTACTATTTTGCAGTCTTTTGAGG
TTATTATTCTGCTCCGAGAAAAGAGCAGACAACCAAACTCATAGAAATTTCACGCTA
AAAAGATTTCTTAGTTTGAAAAATCCATATGGAAAACTGGTTGGCAAACTCAGAAGC
TTAATCCAGGAGTGGGGCTTACCAAAAGAATGGCGTGGTAATAGTGATATTTTATCT
ACTCTAAATTTTAATGGAGGTGGTTTGTTGACGATGGAACCATTAGATAGAATAATA
TTATTGAGATGCATGATCGATTGGAATTGTAGCTACTCCGCACTTTTCCATAATGAAA
TTCAAAGGCTCACTCACTTGAAAGGCGATACAGGGTTCAACCATCAAACTTTTCACG
CCAGCAGGTTTGCTATGTGCGGGGCCAATAACATATTAGATAGTTGTGAAGTTTTAT
GCTCTTTGATGAGTCAAAAGTTGGAAAATCGAAAGAAAAGGAAGCCCAGCGACAAA
GGAAAACTGAGTAAGATCAATAGTCAGATGAAATTTCTAAAGGGTGTTCGGAAATCA
TTGAGCGAAAAAGTAACTACAGACAGATTACGAGCTGCAGTTAAAATAAATGAAGA
ATGGGGTGAATATTTTGCAAACGAATTTACACACACACCAATTGATGATCCCACCGT
AGACGAAATTTACAAGTTAAGGACATCAGAGTTTATGATAGCGAGAATTCCACGAGT
TGGAGATTTCTACTTACCACCATTCTGGATTGGCAACGAATGCAGTTCAGTAAACACT
AGTTACAGTTTCAATGACATGTCAACTTATCTTAATTACTTCGTAAAATTCAAAGAGG
AGGGTACAAAAATACTGCCTGCAAAAACCGCACAAAATGAAAACAAGTGTCAACTTA
AACTTATATATAGGAACACTCCTGCATGTATCCGTAATTTACAGTTCAATGATGTTCA
CTTTGCCGAGGTGCCGCATTGGTTTGAAGTTGCAGGAGACTCGAATTCTTTGAGCAA
CTTCATTGAGTATTTGGAAAGTTTATCATCACTTACTGAAAATGACACCGATGATACA
AAAAAGGGTATAGACAATCTAATTGAGTTTTTGAAAATTTTCAGTATATTCATAAATG
AGACTATTCAGCGCATAACTGCTGCCCCTACAGGCAGTACTGAAGGACGTCATTTGC
GAACAAGCTCTCAGAGAAGAACCACGGTGCATTACTCGAGTGATGTAAATGGTGACG
TTTCCGAGGAAAGTGAAAATGAAGTTGACATTGACGTTAGCGATGACTATGATTCAG
AATATCTGAGTGAGGAGAATACATTGACAAGAAAAGGTGAAGATAGGACAGATAAA
TCTTTTGGGAAAAGAGAATTACACAATGGCGCTAAAGATTGTGATAGGAATTGTGAT
GATATAGAGATCTTCTCAGAACCCGTTAGGCAACTACAAGATAATTCTCGGGAAAAA
AGAAGCTTGAGGCGTAATGCCAGAAAAGGTCTATAG**</refsequence-sequence>
- <refsequence-feature>
<name_type>**GENBANK_ACCESSION**</name_type>
<name_value>**CAA99016**</name_value>
</refsequence-feature>
- <refsequence-feature>

<name_type>**SGD**</name_type>
<name_value>**YOL017W**</name_value>
<url>**http://genome-www.stanford.edu/cgi-bin/dbrun/SacchDB?find+Sequence+%22YOL017W%22**</url>
</refsequence-feature>
- <refsequence-feature>
<name_type>**GI**</name_type>
<name_value>**1419793**</name_value>
</refsequence-feature>
- <refsequence-feature>
<name_type>**ORF_TYPE**</name_type>
<name_value>**Verified**</name_value>
</refsequence-feature>
- <refsequence-feature>
<name_type>**ORF_STATUS**</name_type>
<name_value>**No change**</name_value>
</refsequence-feature>
</refsequence>
- <refsequence>
<refsequence-id>**758**</refsequence-id>
<refsequence-species>**2**</refsequence-species>
<refsequence-cds-start>**1**</refsequence-cds-start>
<refsequence-cds-stop>**2148**</refsequence-cds-stop>
<refsequence-chromosome>**15**</refsequence-chromosome>
<refsequence-sequence>**ATGCTACGGTTTACTCATCGAGGCCTTCCCTCCAGCACTCGTTTTAGAAAC
ATTTTCGTAAGGTTAAATCACATTTATGTACCATGGTTCTATGCCATAGACGTACCCA
ACTCCAAACCCTATTTACCCACATATCAAACTTTACACTCACCCAAAAAGTTTAAGCC
GTTCTCCGTTGATGATTCTAATCGTTTGGAAAAGGCCAGTAAGCGTCAAGAACGCAG
GCCTGTTTTAGTCAACGAAGATTACCTGTTTAAAGTCGACCTCTCTCACATGGAATTG
TCCCCTACCTATTGGGAAGGCCCTACTTATCAAGTACGAAGAGGTGTGTGGTTCGAC
TCTTCAAATCAACCTTTGTCCAGTGATCTTACCTCCGAAATTGAAGGGTTGTATAAGC
AGCTCAAATTCGACGACAGTAATGATGATCCGACCACGACACCTCCTGCAGAATCGC
AAGATATATTTAGGCTCAAAGGTAAATACCCAGTCGATAAAGAAAACGAAGGAGAG
CAAAAAAATGGATCCAGCAATAAAGATGAGAACGAATCTACTTTCAAGTTTATTTTG
TTTGCCAATAAACAAACTGCATTTTTACTATCGGATTTAGATGGAGGAAAACTGCAAT
TAGCCTTTCTAAGGTCTAATCTGGCTCAATCCTTACCGATTAATGCTACAATGATTAC
AAGATCATACAAATATTCATCCTCCGCAACTACTAAACAGACATCCACATCTTTTAAG
GCAGCAAAAACGCCTCAAACGGAAGTAGCAGATGGTAGTAACAGTTCCAAATCGAG
GAGCATTGAAACAAAGTTAGAAAAGAAAGTTTCAAACCTCTTCAATTTATCGGACTT
TTTACAGTTGTTCAATGGTAATGCTAGTAAAGATCAAGATGATGCACAGAGCTTGGA
AAAGCAAATGGAAACAGACTATAACAATGCAGATAACAGTCAAGGCGCTAATGCTA
GTAGCAAAATAGAAGATGGCAAAAATTCGGGCGCAAGCGATAGACAAATTAGAAGT
AACAGAAGAGATGTGGATAACTTGATACTATGCGTTCACGGTATAGGTCAGACTTTG
GGTAAGAAGTACGAATACGTAAATTTCGCACACACGGTAAATCTACTTAGGTCTAAT
ATGAAAAAAATCTACAATAATTCTGAGAAACTACAATCATTAAACACAGCACCTGAT
TATAAAAGCAACTGTAACGTTCAAGTGCTACCCATCACCTGGAGGCACTCGATAAGT
TTCCAAACGGATGCTAAGGAGGAAAATATAGAAAATCCTGACCTACCGACTTTGTCA
CAAGTCACAGTAAATGGAGTGTTACCCTTGAGGAAGCTACTGGCTGACGGTCTACTA
GATATTTTGTTGTATGTCGAACCATACTACCAAGATATGATTCTACAACAAGTAACCT
CTCAATTGAACAAAACATATCGGATTTTTAAAGAATTTAATCCAGAGTTTGATGGGA**

AAGTCCATTTAGTGGGCCATTCGTTGGGTAGTATGATATTATTTGATATTCTATCCAA
ACAAAAAAAAATATGAATTAGAATTTCAAGTAGACAACCTGTTCTTTATTGGCTCACCA
ATTGGATTGTTAAAGTTAATTCAAAGAACAAAAATTGGTGACCGTCCCGAATTTCCC
AATGACTTGGAGAGAAAATTAACCGTACAGAGACCGCAATGTAAGGACATTTATAAT
GTTTACCACGTCTGTGATCCCATTTCTTATAGGATGGAACCTCTTGTTAGTAAAGAAA
TGGCTCATTACGAACAAACTTATTTACCACATTGTAGTGAAGCTTATGGACTAACTTC
TAAAGTTTTGGAATTTGGCGAAAACATATGGAAAGATTTGCCAGGTACTGATGAAAA
TAATTTGCAGTCCAAAAAAACTTCTCCAGAAAAAAAAGAGGTCAAGTTATCAGAAAA
TCTTACGAGAATGCTTACAGGTTTGAATTACACCGGACGTCTAGATTATGCTATGTCT
CCAAGTCTGCTAGAAGTGGATTTTATATCAGCTATAAAATCACATGTTTCTTATTTCG
AAGAACCGGATATTGCAGCGTTTATCCTAAAAGAAATTTTAAGTAAACATGAAAATG
CATCAGAAATATATGTAAAAAGAAAGACTGGTTGA</refsequence-sequence>
– <refsequence-feature>
<name_type>SGD</name_type>
<name_value>YOR022C</name_value>
<url>http://genome-www.stanford.edu/cgi-bin/dbrun/SacchDB?find+Sequence+%22YOR022C%22</url>
</refsequence-feature>
– <refsequence-feature>
<name_type>GI</name_type>
<name_value>1420129</name_value>
</refsequence-feature>
– <refsequence-feature>
<name_type>GENBANK_ACCESSION</name_type>
<name_value>CAA99212</name_value>
<url>http://www3.ncbi.nlm.nih.gov/htbin-post/Entrez/query?db=p&form=6&uid=CAA99212&Dopt=g</url>
</refsequence-feature>
– <refsequence-feature>
<name_type>ORF_TYPE</name_type>
<name_value>Uncharacterized</name_value>
</refsequence-feature>
– <refsequence-feature>
<name_type>ORF_STATUS</name_type>
<name_value>No change</name_value>
</refsequence-feature>
</refsequence>
– <refsequence>
<refsequence-id>4808</refsequence-id>
<refsequence-species>2</refsequence-species>
<refsequence-cds-start>1</refsequence-cds-start>
<refsequence-cds-stop>2160</refsequence-cds-stop>
<refsequence-chromosome>14</refsequence-chromosome>
<refsequence-sequence>ATGTTGTTGGTACATATTATATCTTTCCTTCTGTTCTTCCAGCTTTCGGCC
GCAAAGGCCCCACCCAGTAAAACGTCTCTAATAAATACTCATGAGAGAAGGTCGATA
TATTCATGCTACGTTGGTTTACGTAAAGAAACATGGGGGTTCAATGGGTCCGCTATA
TGTCGGTATGAACCAGCAATCCAATCGATGCTTTACTGTCTTTACGAAGACACGCATG
AGAAAGGGTATTCAAATAAAACTTTGGAGAAAGGTTTTGAAGAAATGAGACAATTTT
GCTATACACCAAAGTTTTTGAACATGACTGATGCCGAGTTTTACACCTCATTGGATAA
TGGAACATACTATATACAAGATCAACCTAAAGCTGGCATCAATATCACTTATCCTATC

AGACTGAACACTACACTAAGAAAAGCATACTATGATGCATACTATGGTTACTACTAT
AACCATGACATTCCGTATTATTTCGGGGGCATTATCTGTGCATACTTTGTGGGTGTCA
TGTTGCTTGCAGGTTTAATTCGTTTTTTGAATTATACCCCAATAAAAAAGATTATGTT
TCAGCAAAAGCTAGTCAATTACGTGAGAGGTTATACTACTCTACCCACTCTTTATGAA
AAGCATGCAGAGCCCTTCTCGTACTTAAAAGTGATAACAGGCTATCTTCCTACTAGGT
TTGAAACGTTGGTTATTTTAGGCTACCTCATACTTCATACCATTTTCATGGCCTACAA
ATATCAATATGATCCATACCACATCATATTTGCCGCTCATAGAGCAGAAGTGGCACAT
TTTGTTGCGTACAGAAGCGGTATACTTTCTTTTGCACACCTGCCACTCATTGTTTTATT
TGCGGGAAGAAATAACTTTCTCCAACTTATTTCTGGCTTGAAGCATACCTCATTCATT
GTGTTCCATAAGTGGCTGGGAAGAATGATGTTTCTTGATGCAATAATTCATGCTGCC
GGCTTTACGAACTATTATTTGTATTATAAAAAATGGAATACGGTTAGATTAAGAGTCT
ACTGGAAATTCGGTATTGCTACCACCTGTTTAGCGGGAATGTTAATTTTCTTTTCCAT
CGCAGCATTTAGAAGACACTACTATGAAACGTTTATGGCCCTCCATATAGTATTCGCA
GCACTATTTCTCTATACTTGTTGGGAGCATGTTACTAACTTCAGCGGTATCGAATGGA
TTTACGCGGCAATAGCAATTTGGGGAGTTGATAGAATTGTACGTATCACCAGAATTG
CACTCTTAGGATTTCCTAAAGCAGATCTACAACTGGTTGGATCTGATTTGGTCCGTGT
AACGGTTAAAAAACCAAAGAAGTTTTGGAAGGCAAAACCAGGCCAATACGTTTTCGT
TTCCTTCTTGCGTCCATTGTGCTTCTGGCAGTCGCATCCATTTACAGTGATGGATTCTT
GCGTAAACGATAGAGAATTGGTCATCGTTCTGAAAGCAAAGAAAGGTGTGACAAAA
CTGGTAAGAAACTTTGTTGAACGTAAAGGCGGCAAGGCATCCATGAGATTAGCTATC
GAAGGCCCTTATGGCTCCAAGTCCACCGCCCATCGCTTTGATAATGTATTATTGTTGG
CAGGTGGCTCAGGGCTTCCCGGTCCAATTTCTCATGCCCTTGAATTAGGAAAGACAA
CAGCTGCAAGCGGTAAAAACTTTGTACAGTTAGTCATAGCAGTAAGAGGACTAGACA
TGCTCAACGCGTGTAAGAAAGAACTAATGGCATTAAAGGGCTTGAATGTTCAAGTTC
ACATTTATAATTCTAAGCAAGAGCTAGCTTCGGCTGAAAAAATTTCCTCAAATGAAG
TCAAAAACGGTGAAACGACAGCAGAGAAGGCCCCATCTAGTCTAAGCAATTCAGAA
AAAGCTCCTTCTGAAAGTGAAAATACAGAACTACCTCTTTCCCTGAATGACACGTCTA
TCTCCGATTTAGAATTTGCCACTTTCCATGTTGGAAGGCCAAATGTTGAAGAAATACT
AAATGAATCTGTTAACCATTCTGGTTCACTTGCCGTCGTATGTTGCGGACCACCTATT
TTCGTCGACACCGCTAGAAATCAAACTGCCAAAGCTGTTATCAGAAACCCATCAAGA
ATGATTGAATACTTGGAGGAATACCAAGCCTGGTGA</refsequence-sequence>
- <refsequence-feature>
<name_type>GENBANK_ACCESSION</name_type>
<name_value>CAA96342</name_value>
<url>http://www3.ncbi.nlm.nih.gov/htbin-post/Entrez/query?db=p&form=6&uid=CAA96342&Dopt=g</url>
</refsequence-feature>
- <refsequence-feature>
<name_type>SGD</name_type>
<name_value>YNR060W</name_value>
<description>Similar to ferric reductases Fre1p and Fre2p</description>
<url>http://genome-www.stanford.edu/cgi-bin/dbrun/SacchDB?find+Sequence+%22YNR060W%22</url>
</refsequence-feature>
- <refsequence-feature>
<name_type>GI</name_type>
<name_value>1302584</name_value>
</refsequence-feature>
- <refsequence-feature>
<name_type>GENE_SYMBOL</name_type>
<name_value>FRE4</name_value>

</refsequence-feature>
- <refsequence-feature>
<name_type>ORF_TYPE</name_type>
<name_value>Verified</name_value>
</refsequence-feature>
- <refsequence-feature>
<name_type>ORF_STATUS</name_type>
<name_value>No change</name_value>
</refsequence-feature>
</refsequence>
- <refsequence>
<refsequence-id>4202</refsequence-id>
<refsequence-species>2</refsequence-species>
<refsequence-cds-start>1</refsequence-cds-start>
<refsequence-cds-stop>2163</refsequence-cds-stop>
<refsequence-chromosome>13</refsequence-chromosome>
<refsequence-
sequence>ATGAATTCGAATGAAGATATACATGAAGAACGCATTGAAGTTCCACGAA
CTCCCCATCAAACCCAGCCAGAGAAAGACTCTGATCGCATCGCTCTCAGGGATGAAA
TATCAGTACCAGAAGGCGATGAAAAAGCATATTCGGATGAGAAAGTAGAAATGGCA
ACCACAAATGCATCCAGTAACTTTGGCTCAAATGAAAGTGCAAAAGACGGCGAATCA
ATCGGCGCCTTTTCAAATCCCCATGAGGCTCTGATGCAGTCAAAATTGAGAGAAGAA
TCCCAGAGTAAAACCATACTACCATCCGATGACCTCAGCCAGCAATTAGAAACCGAA
GAATCCAAAGTTGAAGAGGCTCTAAAAAGAATAACCTCACCCCCATTACCACCGAGG
GCTGACTGCATAGAAGAATCTGCATCTGCTCTTAAATCCTCATTGCCTCCAGTGCTAG
CAGGAAATAAAAATGATCAAGCCCCCCTTGACCGACCACAGTTACCTCCGAGGCAGG
TAGTTAATGCGGAAACTCTTCATTTGAAGGCGCCCCATGGTAATGCGACTCCCTCCAA
ATCACCAACGAGTGCTGTAGGTAATTCTTCTTCATCGACCCCTCCAACCTTACCACCA
CGCCGTATAGAAGATCCTTTAGACTTGGCTGCTCAAAAACATTTCCTAGCGAGTACAT
TTAAGAGAAATATGCTCTTTTATAAAAGTGAAGATAACTCTATCAAATGTGATTTGG
ATAAAAATATACTAAATTTAAAAGAAGATTCAAAAAAAATTAACAACAATGAAATTC
CTGAAGAGGTTAGTTCATTTTGGTTAAAGGTTATCGGCGACTATCAAAATATCTTAAT
AAACGATATCGAAACCTTACATTTCCAACTATCTCGAGGTATACCAGCCGCTTATCGA
TTGGTAGTCTGGCAGTTAGTAAGTTATGCAAAATCGAAATCCTTTGACCCTATATATG
AAACGTATCTAACGGAAATGGCACCTTTTGACGTCCAGGAATTTGAAAATCAGCTAA
AAATGATGGACGAAGTTCCCTCTGAATACGTAAAACGCATAAGTAATGTGTTAAAGG
CTTATTTACTTTTTGACCCAGAGTGTGAGTTTTCTACCGACATTGCTTATATTATCAAT
ATGATTCTTGACGTTTGCGAGGAGGAGGCAAATGCATTTGGTCTACTTGTACGCCTA
ATGAAAGTTTACGGTTTAAGACTCTTGTTCTTACCAAGTGCTTCTGAGATCGATATTC
TTTGCTATAAATTTGATAGACTTGTAGAAGAATTTTATCCTGAAATTCACAACCACAT
GGTTGAAAAAGGCGTCCGTTCTTCCATGTTTTTGCCCGGTTTCTTCACGACACTGTTT
CAAAAGAAACTTCCCACTGAAATTCAACCACGTATCGGTGATATGGTATTTTTAGAA
GGTATTGACTCTATTATGAGAATTTTAGCCACACTTTTATCTAATTCCCGAGACCATC
TGTTAAAAATGGGTTTTGATGATATGCTGGAGCTATTGAAATCAGGTCTGTTAGATG
CTTATATTAAGCAGAATGATGGTACACGCGGTGACACTCTTCTCTCAAATGAATGTAT
GGATAAATTACTACAAGACTCTATGATGAAAGTAGCAATAACCCCAAAGACTATGAA
GAAATATTCTTCTGAATATGAGGAAATTCACAGATTGGACAATGAAAAAGAAGTGCA
ATATAAGTCTATCACGGAAAAAAATTTACATTTACAAAAGCATGTTCGTAAATTAGA
AAACGACTACACATCTTTAAATAGAGAGCATGTGACAATTGCGAACGAGTTAGTGAA
AAATCGGCTTAATATCGAGTCTGTATTAAATGAAAATAATGGTTACAAACTTCAAATT
TTAGATTTGAAGAAAAAGTTGGATTCAGAAAAAAAGAAGCAAGTTTTAGGTGTATAT
GTGCCCAATGATTTAAAGAAAGATTTGGAAGAGACAATGAAAAAGAATACCCAAGT

AATGGACGAAAATTTGAAATTGCAGGATAGGATTTCAGAACTAGAAAGGCTTATTGA
AGAAATAAAAACTGCAAACAAAAATGGTACGTTATTCGAATATTCTAATTCTAAAAA
TAATCCTTTGGGAGCCGGCTGGTCAGGATTTAAAAAGGTTTTTAAATAG</refsequen
ce-sequence>
– <refsequence-feature>
<name_type>GENBANK_ACCESSION</name_type>
<name_value>CAA87813</name_value>
<url>http://www3.ncbi.nlm.nih.gov/htbin-
post/Entrez/query?db=p&form=6&uid=CAA87813&Dopt=g</url>
</refsequence-feature>
– <refsequence-feature>
<name_type>SGD</name_type>
<name_value>YMR192W</name_value>
<url>http://genome-www.stanford.edu/cgi-
bin/dbrun/SacchDB?find+Sequence+%22YMR192W%22</url>
</refsequence-feature>
– <refsequence-feature>
<name_type>GI</name_type>
<name_value>642284</name_value>
</refsequence-feature>
– <refsequence-feature>
<name_type>ORF_TYPE</name_type>
<name_value>Uncharacterized</name_value>
</refsequence-feature>
– <refsequence-feature>
<name_type>ORF_STATUS</name_type>
<name_value>No change</name_value>
</refsequence-feature>
</refsequence>
– <refsequence>
<refsequence-id>4254</refsequence-id>
<refsequence-species>2</refsequence-species>
<refsequence-cds-start>1</refsequence-cds-start>
<refsequence-cds-stop>2175</refsequence-cds-stop>
<refsequence-chromosome>13</refsequence-chromosome>
<refsequence-
sequence>ATGTTGTCTCAAACCTCCATACCGGAAGTGAAAGAAGACGTGATAGGCT
ATGCGCTACACCAGAGGAGAGCCAGAGTGGGACAATTCCAAGACTTGGGTCCGCCT
GATTTAATTACTTTGATCAAATCGTTACCTTCATCCTCGAGCACAACAACTGCTACTG
CCTCCGCCAACGATAACGGAGCAACTTCAAACATCAATGGACAAGATCCTACGACCA
TAGTTACGGAGTTACATTCCCATGACAAATTAAAGGGGCAGATCGGCACTTTCTTTTA
CTGTATGGGTATCGATACTTCGGATCCAACTTCTATTACAATCTTTGCCAAAAAGATA
ACCGATCTTTTCTTAGACACGCCCCAAATTTGGTTTGGTAAGAAGAAGCACTTTCACG
TATCGAAGATTTCTATCAGTTCTTGGAATGCGTTTAGGAAATATGATGTTAACATTAT
AGTTCACATTCCAGGAACTGTGCAAACCTACATTATAAATAGCGACGGTGAACAATC
GCAACTTCCCTCCGTGGCAGAAGCGTCATCTGGCCGCAACTCACAGGATTTAAACGT
CAACATGATTTGGGCGGAAACGTTTATGAGTGGTATTGTACGTGACATTATGATTAT
GAAAGATAATCGTGCGGATGGAGAATCCCAGAATTTGGTAGAAACACTAATTTTCAA
TCCATTTACCTCGGGTGAGTTGGAAGATGTTGCCAATAACTTTATTAAACTGTTCCCC
TTGGTCTATGAAAAAGGTGTTTATTTAGATGCACCTACTCACGTTTTAAATCCCTCGT
TAACCAATAATTATTTAGTGGAAACTTTAGTGGAAATAGTTAGGTTAACTAAGAGTT

TGGAAGCATGTCGTAAAATGCTCAAGAAGTTAATAGAAATTCATCCAGAAGCAGTAA
TAATATTAATTCGTGTTTATTTTGCGTGCGATTTAGAGATAGATGCAGTTGACCTGAT
CAACGAGCAACTAAATTCCCCCTCTTCGTTCTTAGCCGACGATTCAAAGACTAGCCAT
ATCCAGCTAATCTTCAAATCCGAATTATTAAGTATTCAAAGTGAATTCTTACTGGATG
TCAAGAGAGATTACAAGCTTGCTAAGGAAGTAGCCATGGAGGCTGTTAACTGTGCAC
CAAACGAATTTAAAACTTGGTATTTATTGACTAGAATATACATTAAACTAAACGATAT
GTCAAATGCCTTGCTATCGTTAAACGCCTGCCCCATGTCACAGGTTAAGGAAAAATA
CGTTCTTAGAAGAATTGCGCCTATTACTTCAGATGAAAATCTTCATCTGCCATTACCA
TTGGATGCCTCAATTGAGGAGATTTCGTCATTGAACCCCATGGATGTCCAGTTGGAG
CAAAAGTCCGCAGATCCAAACCTAGTCAATCTCTCTGCGTCAAGTTTAAAGTCTACTT
TCCAACTAGCCTATAAATTATTGACAGAAATTGTCCAAATAACAGGATGGGAGCAGC
TGTTGAAATATAGATCAAAAATTTTCGTTATGGAAGACGAGTACCAAGGCTCCACTT
CTTCCATTGATGAAGCAGAAGTTCGCGGTAATGATATATCCAAAATGAGATCAAAGA
GGCTATGCGAAAGATGGTTGGATAATCTCTTCATGTTACTATACGAAGATTTGAAAA
CTTACACCGATTGGCAATCAGAGCAATTGTATTTTGATGCTCAAAACAGTAAATATCA
CAAATTAACTGTTGAATGGGAATTATTCGGCCTTTGTGCGAAAAGACTGGGACATCT
TCCAGAAGCTGCGAAGGCTTTCCAAATTGGGCTTTCCCAAAGATTTTCTCCAGTATGC
GCAAAGAATCTATTACAGTTTTACATTGACGAGCATAAGCGTATTAGGAGGGATTCA
GTCTCAGCAAACTCCGAGTTAACCTCTTCTCAAATATTGTCAAGTATCAATGACATCG
ACAGCTCAATCATCGATCTAGTAGTCAAGATTTGTTGCTGGAATCATCGTTGGTACAT
CGAGTTCTCAATAATATTAATAGATGCTCTGAGTGTTGCAGTACAAGATATGGGCAT
TACTAAAGTACATAATGAAATTGCCTCTAGATTTTCTGACCCAGTAGCCCAATTGATT
GACGATAACATTCTAAATTTTCTGAAGAATTTCACGAATGACACTTTCGATAATTAG<
/refsequence-sequence>
– <refsequence-feature>
<name_type>GENBANK_ACCESSION</name_type>
<name_value>CAA90208</name_value>
<url>http://www3.ncbi.nlm.nih.gov/htbin-
post/Entrez/query?db=p&form=6&uid=CAA90208&Dopt=g</url>
</refsequence-feature>
– <refsequence-feature>
<name_type>SGD</name_type>
<name_value>YMR237W</name_value>
<url>http://genome-www.stanford.edu/cgi-
bin/dbrun/SacchDB?find+Sequence+%22YMR237W%22</url>
</refsequence-feature>
– <refsequence-feature>
<name_type>GI</name_type>
<name_value>887618</name_value>
</refsequence-feature>
– <refsequence-feature>
<name_type>ORF_TYPE</name_type>
<name_value>Uncharacterized</name_value>
</refsequence-feature>
– <refsequence-feature>
<name_type>ORF_STATUS</name_type>
<name_value>No change</name_value>
</refsequence-feature>
</refsequence>
– <refsequence>
<refsequence-id>4790</refsequence-id>

<refsequence-species>**2**</refsequence-species>
<refsequence-cds-start>**1**</refsequence-cds-start>
<refsequence-cds-stop>**2178**</refsequence-cds-stop>
<refsequence-chromosome>**14**</refsequence-chromosome>
<refsequence-sequence>**ATGACATTATCTTTCGCTCATTTTACCTACCTGTTCACAATATTGTTGGGA
TTAACTAATATTGCCTTGGCATCTGATCCAGAAACGATTCTAGTGACGATAACCAAG
ACAAACGATGCAAATGGGGTTGTTACAACTACAGTTTCACCCGCGCTAGTCTCCACA
TCCACTATCGTTCAAGCTGGCACTACGACATTGTATACGACTTGGTGTCCATTGACGG
TATCCACTTCATCTGCTGCCGAAATAAGTCCTTCAATATCGTACGCTACTACCCTATC
CAGATTTAGTACTTTGACATTATCTACAGAAGTCTGCTCCCATGAGGCATGTCCTTCG
TCATCGACGTTGCCAACCACCACCTTATCTGTGACTTCCAAGTTCACTTCATATATTT
GCCCTACTTGTCACACAACCGCTATCAGCTCATTATCCGAAGTAGGAACTACAACCGT
GGTATCATCCAGCGCCATTGAACCATCAAGTGCCTCTATAATCTCACCTGTCACCTCT
ACACTTTCGAGTACAACATCGTCCAATCCAACTACTACCTCCCTAAGTTCGACATCTA
CATCTCCAAGCTCTACATCTACATCTCCAAGCTCTACATCTACCTCATCAAGTTCGAC
ATCTACCTCATCAAGTTCGACATCTACCTCATCAAGTTCGACATCTACATCTCCAAGT
TCGACATCCACATCTTCAAGTTTGACATCCACATCTTCAAGTTCTACATCTACATCCCA
AAGTTCTACATCTACCTCATCAAGTTCGACATCTACATCTCCAAGCTCTACATCTACCT
CATCAAGTTCAACATCTACATCTCCAAGTTCTAAATCTACTTCTGCAAGCTCCACTTCC
ACTTCTTCATATTCAACATCTACATCCCCAAGTTTGACTTCTTCATCTCCAACTTTGGC
TTCCACTTCTCCAAGTTCAACATCTATTAGCTCTACTTTTACTGATTCAACTTCATCCC
TTGGCTCCTCTATAGCATCTTCATCAACGTCTGTGTCATTATACAGCCCATCCACACCT
GTTTACTCCGTCCCTTCGACTTCGTCAAATGTTGCAACTCCTTCTATGACTTCTTCAAC
TGTTGAAACAACTGTTAGTTCACAAAGTTCGTCTGAATATATCACCAAATCCTCAATT
TCTACTACTATCCCATCATTTTCCATGTCTACATATTTCACCACTGTTAGTGGAGTCAC
TACAATGTATACGACATGGTGTCCTTATAGCTCTGAATCTGAGACTAGCACATTAACC
AGTATGCATGAAACGGTTACAACAGACGCTACAGTCTGCACTCACGAGTCTTGCATG
CCCTCGCAGACAACAAGTTTGATTACATCTTCTATAAAAATGTCCACTAAAAACGTCG
CAACTTCTGTAAGCACCTCAACGGTTGAATCCTCATATGCATGCTCCACATGTGCTGA
AACGTCACACTCGTATTCTTCCGTGCAAACAGCTTCATCAAGTTCTGTAACACAGCAG
ACCACATCCACAAAGAGTTGGGTAAGTTCAATGACAACTTCGGATGAAGATTTCAAT
AAGCACGCTACCGGTAAGTATCATGTAACATCTTCAGGTACCTCAACCATTTCGACTA
GTGTAAGTGAAGCCACGAGTACATCAAGCATTGACTCAGAATCTCAAGAACAATCAT
CACACTTATTATCGACATCGGTCCTTTCATCCTCCTCCTTGTCTGCTACATTATCCTCT
GACAGTACTATTTTGCTATTCAGTTCTGTATCATCACTAAGTGTCGAACAGTCACCAG
TTACCACACTTCAAATTTCTTCAACATCAGAGATTTTACAACCCACTTCTTCCACAGCT
ATTGCTACAATATCTGCCTCTACATCATCACTTTCCGCAACATCTATCTCTACACCATC
TACCTCTGTGGAATCGACTATTGAATCTTCATCATTGACTCCGACGGTATCTTCTATT
TTCCTCTCATCATCATCTGCTCCCTCTTCTCTACAAACATCTGTTACCACTACAGAAGT
TTCCACTACTTCAATCTCCATACAATACCAAACTTCATCAATGGTAACAATTAGCCAA
TATATGGGCAGTGGATCGCAAACGCGTTTGCCATTAGGAAAGTTGGTCTTCGCCATC
ATGGCAGTTGCTTGCAATGTAATTTTCAGTTAA**</refsequence-sequence>
- <refsequence-feature>
<name_type>**SGD**</name_type>
<name_value>**YNR044W**</name_value>
<description>**anchorage subunit of a-agglutinin**</description>
<url>**http://genome-www.stanford.edu/cgi-bin/dbrun/SacchDB?find+Sequence+%22YNR044W%22**</url>
</refsequence-feature>
- <refsequence-feature>
<name_type>**GI**</name_type>

<name_value>1302552</name_value>
</refsequence-feature>
- <refsequence-feature>
<name_type>GENE_SYMBOL</name_type>
<name_value>aga1</name_value>
</refsequence-feature>
- <refsequence-feature>
<name_type>GENBANK_ACCESSION</name_type>
<name_value>CAA96325</name_value>
<url>http://www3.ncbi.nlm.nih.gov/htbin-post/Entrez/query?db=p&form=6&uid=CAA96325&Dopt=g</url>
</refsequence-feature>
- <refsequence-feature>
<name_type>ORF_TYPE</name_type>
<name_value>Verified</name_value>
</refsequence-feature>
- <refsequence-feature>
<name_type>ORF_STATUS</name_type>
<name_value>No change</name_value>
</refsequence-feature>
</refsequence>
- <refsequence>
<refsequence-id>5039</refsequence-id>
<refsequence-species>2</refsequence-species>
<refsequence-cds-start>1</refsequence-cds-start>
<refsequence-cds-stop>2181</refsequence-cds-stop>
<refsequence-chromosome>15</refsequence-chromosome>
<refsequence-sequence>ATGGCAAGTGTCATGTCAAATAATAATAATAATAATAATAATAATAATGC
CAGTTATATGTTTACGAACCCTTTGTCCAACACAGGTGGTGGACTCATAAATGAAAT
AAAAGATGCAATAAATGAAATGGAACAATTAAAGGTTCTAGAGTTAAAGCAAATATG
CAAATCTCTGGATTTATCCATAACGGGCAAAAAGGCTGTCTTACAAGACCGAATAAA
ACAATTCTTAAGAAAATCCTGTGATATTGGACATATAGATCCGTGGAGGCCTAAGGC
TATTAAGATTCTTATCGCGAAAGTAAGGATAAACTCATCATTACCAAAATACTCTACC
CTTTGGGAAACTTTAAAGACGGGTGCGTTCAAGCATCCTGTAGCATCGGGGCAACTA
CCCGTGACAGCTCTCCAGAGTACTGCATTGCCACCTTATTCCCAACAACAGGCCTTAG
CATATTCTTTTACCTCACCGTTTTACAAACCAATAGTACAAATACCGGATGCCAATAA
AAAATTGAAGCAATCTGCAGGAAGAGGTTGTACAAAAATGAAGTTCAAGGTAAGTA
AATCTAATCATGATCTTTTGAAATCTAACAAGAGTTATAAACTGTATTTGTTTTCTGG
CTTTTCAATACCGTTTATATATGAAACTGTGGGCCATGAGGCAATAGATTTCCCGTAT
CCCTGTGAACTAGTGTTTAATGGCACCAAACTAGAGGACAACGTGAAAGGTTTAAAG
AAACAAAATGGGACAGGTAATCCTGCAAATTTAACGCCTTATCTTAAAGTACCGACC
GAGATGAATCACCTAGACTTACATTATCTCAATATTGATAAAGAGTACTCTATAAGTT
GCTTTATAGTGGAAGTTTTTTCACCTGAAGCTCTTCTGGGGAAAATTCTGAAAAGGCC
CAAAATTATCAAGCAAGCCACCACAGCATACATCAAAAGAACCTTGAATGAGCAAGA
TGATGACGATATTATTACAACCTCCACGGTGCTGAGTTTGCAATGTCCTATATCTTGT
ACAAGGATGAAGTATCCGGCGAAGACTGACCAATGTAAGCATATCCAATGTTTTGAT
GCTCTCTGGTTTCTTCACTCTCAGTCACAAGTCCCTACATGGCAGTGTCCGATTTGTC
AACACCCTATAAAATTCGACCAGTTGAAGATTTCCGAATTTGTAGATAATATTATTCA
AAATTGCAATGAAGATGTTGAGCAAGTTGAGATATCTGTTGATGGCTCATGGAAACC
GATTCATAATAGTTCGGCCGTTATAACGGATACTGTTAACCAAAATCACAGTGTGAA

AAACGAGAACCAAGGCACTGTCAAACAAGAGCAAGATTATGACAGCCGAAATGCCT
TCGATACTAATTTGCGTAATGGTTCAAACCACAACGAACCCGAAATAATATCTCTGG
ATAGCTCAGATGATGAAGCCTTTATTCCGGCTAGTAAAAGCTTCCCAACGCACGTGA
ACCCCCGTAATGACCAATTAAGAGCAGATATATTTCCCTCAGAATCGGAAGGATCCA
GTGACTACAACCCCAATCATACATCAACGCCGAAAGGTTCTCCAACTATGGACCAAG
ATAATTATCAAGATGCTTTTCAAATGAGGTCATTTTTGAACCAAGGCGCTACTACAAA
TATAAATGACACACCGACGAACAATAGCAGTATTAATTCTTTCGTTACGGCGACGAA
TGGTGACAGCAGAATATTCTACAATAGAGGGCCATCAACGCCACTATTACCGGCTGT
TCTACAAAATTTGACTAATCAAACGGAAGCGCAGAGAAACCCCTACGGACCTAATTA
CAATACCACAGCTCAAGATCGTAATCTTTTGGGAATCGAAGGTGATTTGCCTCCAATC
CCCCCTGTGGACCCAAATTCAGAAGCAGAAACTGAACTACCAACAAGGACCACATCG
GCGGCACATCTCCCTCCATATATACACGTGAGCACCAGCGGCCATGGGGATGATGGA
AAAATTAGGAAGAGAAGGCATTCTAATGTTTCTATCTACATTCCGAAGAATCCATAC
GCCACCCTTATGAAACGGAGACCACAGGCAAATCACGCCATAATGAATAAAACTCTG
GCCCAAACTAATGACTTTAACACATCGGCTCAAGATAACTCGGAAGTGGTCGATTTA
ACATCAGATTGA</refsequence-sequence>
- <refsequence-feature>
<name_type>GENBANK_ACCESSION</name_type>
<name_value>CAA99362</name_value>
<url>http://www3.ncbi.nlm.nih.gov/htbin-post/Entrez/query?db=p&form=6&uid=CAA99362&Dopt=g</url>
</refsequence-feature>
- <refsequence-feature>
<name_type>SGD</name_type>
<name_value>YOR156C</name_value>
<description>interacts with C-terminus of CDC12</description>
<url>http://genome-www.stanford.edu/cgi-bin/dbrun/SacchDB?find+Sequence+%22YOR156C%22</url>
</refsequence-feature>
- <refsequence-feature>
<name_type>GI</name_type>
<name_value>1420389</name_value>
</refsequence-feature>
- <refsequence-feature>
<name_type>GENE_SYMBOL</name_type>
<name_value>NFI1</name_value>
</refsequence-feature>
- <refsequence-feature>
<name_type>ORF_TYPE</name_type>
<name_value>Verified</name_value>
</refsequence-feature>
- <refsequence-feature>
<name_type>ORF_STATUS</name_type>
<name_value>No change</name_value>
</refsequence-feature>
</refsequence>
- <refsequence>
<refsequence-id>754</refsequence-id>
<refsequence-species>2</refsequence-species>
<refsequence-cds-start>1</refsequence-cds-start>

<refsequence-cds-stop>2193</refsequence-cds-stop>
<refsequence-chromosome>15</refsequence-chromosome>
<refsequence-
sequence>ATGATTTCTGTTTGCCCACAAAATGACTTGCAAAAATGCTACAGAAGCCT
CACATTCGATGTTCCAGGACAACAATTCGAAGAGAGAAATGAACAAAACCTTAAAAA
ACGGGCCAAAAAGAAAGGCAGTTTCCAACCATCTGTTGCCTTTGACACAGTGCCTTC
CACCGCTGGTTATTCTTCTATAGACGACAGCAGGGAAGGATTCAAAGGTGTACCTGT
TCCCAACTATTACACGATGGAAGAGTGCTATGACGATGAAACAGACTCTTTTTCGCC
AAATTTGCAATATTATTTGAGAGATACATTCCAATCATCACCTTTTCTGAATACTAGA
AAAGAGAACAAATCTGAATCCAGTAGTTTTCCAATGAGATCCTCAAAGTTGTTGGAA
AAGAATTCTGACATCAAAAAATATTTCTTGGTATCCAAGAATGGAAAAATAGTGAGG
AGAGACTATCCAAGCACGCCAGTAATTGTCAACGAAACGTTGATGATAAACAGGTTT
GAAAAGAACTGGATAAAGTTATGGCGCCAAAGAAAACTACAAATAAATGAAAGGCT
GAATGACAAAAAAAAATGGTTTACTTACCCAGAACTTATCTTCTCTGAAGAGCGTAT
TAAACCGTTATATAGAGGAGATGATAGTGCACCATGTACAAAAGAACAAAAAAGAA
AGCATAAAATACTTCAACAAAAGGTCGGATATCCCAATAACCCTAAGACAATAGTTT
GTCACATTAACGGAAAAAAACATACGTGGGTTGCCCTAGACTGGACAGTCTACAAGT
TTGCACGAAATCTTGATCACATTGTTGTCATAACTACACTGCCAAAAATGATTTCTAA
CAGGAAAAAAACTGCAAAAGATGATACAGAATGGGCACCGGGATATCAAAAAGAAG
TAATAGATCAAAAATTAAACGACATTTTTGATTATATTTTACAGCTAGTAAAAGTGGT
CAAAATATCCGTCAAAATTACTTTAGAAATAATTGTAGGCAAAATTAAAAAAAGTCT
GGTAGATGTCATTAATGTCCATACTCCAGATTTCTTAGTTCTTGCTACTTTAAAGCAC
GAGCGAAATGAGAATCTTATTACATATAAATCCAAAAAGCTGACAGATGTCTTTCCT
GTTAGTTATCCGATTCCCACATTTGTTGTTCCCTCGAAACGAATGTATTCGTTCGAAC
TGAATCTACAAAGAGAAGTAAATGAACATTATGTCTCAAAAAATCATATGAAGCACG
AACACACTGACGTTGAGAGCATGAGCAGTTCAATGTTCAAAAAAAATACAATATCAG
ATATTTCTTCACATATTTCCGTAGATTCGTACGCCGAAGATTTCAAAAGGCAAGGCTA
CATCAAAAAGCAGTTCAACACCTCTAATGATTCCATTCCAAGAAAATTGACCGGTCTC
GCCCAGCATTCAAGAAGGAAGATCACGGGTGATATAGAAAAATTACAAGACGATGA
GAAAGATAGAGAATGTACTAAGGAAAAACTTTTGTTGAAGAAAATTGATATCATAAT
TAGAGAGTCATTGAAGTCTTCTTTAGCGATAGAGACGTTGCCTGGTAAAAATGTATC
GCAGTCCAGTCACGGTGACCAAATTTCCAGCTTTAAGAATGCTTTGATAGGCAATGG
GTCGAAAAACACAAAGTTTAGAAAATCTTTAATACCATATTCTTCCTCAGAGGAACA
AAATACCACAACAACTATTAAACTCAGTAGCTCGCCTACGTCCCAAATCAAGTTTGCA
ACCTCTGTAAAACACAAAGATGGAAGAGCCGCCCTTGGCAAAGCCAGAAATCTGCCT
GATATAAGGCACAGTATTTCCTTCGACAAAGAAAATTCCTTTGATCCATCTGATAAAA
GCAGTAGTGTTGATAATAGCATTCCTTTGAGGAAAGTTAAAAGTGCCGGTGCGTTAA
GAAAAGTCAAAACTAATGACTCCTCAAGTAGTGCAGGGTCAAAGAAAAGCTCGTCTA
GTTTTAGTACTGTGAACACCTTCACTGGGGGTGGAGTTGGGATTTTTAAGGTGTTTA
AAAGTGGAAGTTCCTCTGGAAATAAATCATCCAGTAGAAGGAATAGTAGCAGTGGC
GATGTTTTTGAAAGTGATGATCGTAACGACAAGAAAAAGAAGAAGAAAAAAAAGAA
GAAATCATTGTTCTTATTCGGCAAAATATGA</refsequence-sequence>
- <refsequence-feature>
<name_type>SGD</name_type>
<name_value>YOR019W</name_value>
<url>http://genome-www.stanford.edu/cgi-
bin/dbrun/SacchDB?find+Sequence+%22YOR019W%22</url>
</refsequence-feature>
- <refsequence-feature>
<name_type>GI</name_type>
<name_value>1420123</name_value>
</refsequence-feature>

- <refsequence-feature>
<name_type>GENBANK_ACCESSION</name_type>
<name_value>CAA99209</name_value>
<url>http://www3.ncbi.nlm.nih.gov/htbin-post/Entrez/query?db=p&form=6&uid=CAA99209&Dopt=g</url>
</refsequence-feature>
- <refsequence-feature>
<name_type>ORF_TYPE</name_type>
<name_value>Uncharacterized</name_value>
</refsequence-feature>
- <refsequence-feature>
<name_type>ORF_STATUS</name_type>
<name_value>No change</name_value>
</refsequence-feature>
</refsequence>
- <refsequence>
<refsequence-id>4298</refsequence-id>
<refsequence-species>2</refsequence-species>
<refsequence-cds-start>1</refsequence-cds-start>
<refsequence-cds-stop>2199</refsequence-cds-stop>
<refsequence-chromosome>13</refsequence-chromosome>
<refsequence-sequence>ATGACCACACAAATAAGGTCTCCCCAAGGCTTGCCGTATCCGATTCAGAT
CGATAAACTCATTCCAAGCGTAGGTTCGTACCTTCATGAGGGTGATAGGTTGCTTGT
CTATAAGTTCTGGTATTTGGTGGAAAGGGCCTCAGACACTGGTGACGATGATAACGA
GCACGATGTTTCCCCCGGCGGCAGCGCCGGAAGTAATGGTGTCTCTCCGCCAACCAA
ACAACTGCGTGAATCCATAGAATTCTTCGAAAGTCCCTACGAGGGGGACTTGATTAG
TTGGAATGTAGACGTAGGAGATGAAGTAGCCACTGCTAACCAAGTGATTTGCGAAAT
AAAAAGGCCATGTAACCATGACATCGTGTATGGCGGGCTATGTACTCAATGCGGGAA
AGAAGTTTCTGCAGATGCCTTCGATGGTGTACCACTAGACGTTGTCGGGGACGTGGA
TTTACAGATTAGCGAGACAGAAGCCATTAGAACAGGCAAGGCATTGAAGGAGCATTT
GCGACGGGATAAGAAACTTATCTTAGTGGTGGATTTAGATCAAACCATCATCCATTG
TGGCGTGGACCCTACGATTGCAGAGTGGAAGAACGACCCCAATAATCCGAATTTTGA
AACATTAAGAGACGTCAAGAGCTTTACGTTGGATGAGGAGTTAGTACTCCCACTTAT
GTACATGAATGACGATGGTTCTATGCTGAGACCGCCTCCCGTAAGAAAATGTTGGTA
CTACGTGAAGGTAAGGCCCGGTTTAAAGGAATTTTTCGCTAAAGTGGCGCCTCTTTTT
GAAATGCATATCTACACAATGGCTACAAGGGCTTATGCCTTGCAAATTGCCAAAATC
GTGGACCCTACGGGCGAATTGTTTGGCGATAGAATTCTGTCGCGTGATGAAAATGGG
TCTCTAACAACTAAATCATTAGCTAAACTTTTCCCCACTGACCAGTCAATGGTTGTAG
TTATTGACGACAGAGGTGATGTTTGGAATTGGTGTCCAAACTTGATCAAAGTTGTTC
CTTATAACTTCTTCGTTGGTGTAGGTGACATTAATTCTAATTTCTTGCCCAAACAATCT
ACAGGAATGCTACAACTAGGGAGGAAGACAAGACAGAAGAGCCAAGAATCTCAGGA
ACTGCTGACGGATATTATGGATAACGAAAAAAAACTACAAGAAAAGATAGATAAAG
AAGTTAAACGCCAAGAAGAAAAATTGAACCATCAACTGGCGACTGCTGAGGAGCCTC
CTGCGAACGAATCCAAAGAAGAGTTGACCAAGAAACTAGAATATTCAGCATCTTTAG
AAGTCCAACAACAAAATCGACCCTTGGCCAAACTACAAAAACACCTGCATGATCAAA
AACTACTAGTTGATGACGATGATGAACTATACTACTTAATGGGTACGCTATCAAACA
TTCACAAAACTTATTATGATATGCTCTCACAACAAAATGAGCCAGAACCAAATCTGAT
GGAAATCATACCAAGTTTGAAGCAAAAAGTCTTCCAAAATTGCTATTTTGTTTTTTCA
GGATTAATACCCCTCGGGACCGATATTCAAAGGTCGGACATAGTGATATGGACGAGT
ACATTTGGTGCCACTTCCACTCCAGATATAGATTACCTGACGACACATTTAATCACCA

AGAACCCTAGCACTTATAAAGCGCGTCTAGCGAAAAAGTTCAACCCACAGATTAAAA
TTGTTCACCCAGATTGGATATTTGAGTGTTTGGTAAATTGGAAAAAGGTGGATGAAA
AACCCTACACGTTAATCGTGGACAGCCCCATCTCCGACGAGGAATTACAAAACTTTC
AAACACAATTACAAAAAAGACAAGAATATCTGGAGGAGACCCAGGAACAACAGCAT
ATGTTGACATCACAAGAAAATCTAAATTTATTCGCTGCTGGTACTTCATGGTTAAACA
ATGACGACGATGAAGATATTCCGGACACAGCTAGCGACGACGACGAAGATGACGAC
CACGACGACGAAAGTGATGACGAAAACAACTCGGAAGGCATTGACAGAAAGAGGAG
CATCGAGGACAATCATGATGATACATCACAAAAGAAGACCAAGGCGGAACCTTCTCA
AGATGGTCCTGTACAACATAAAGGAGAAGGGGATGACAACGAAGACAGCGATTCGC
AGTTGGAGGAAGAGTTGATGGATATGCTGGATGATTAG</refsequence-sequence>
- <refsequence-feature>
<name_type>GENBANK_ACCESSION</name_type>
<name_value>CAA89775</name_value>
<url>http://www3.ncbi.nlm.nih.gov/htbin-post/Entrez/query?db=p&form=6&uid=CAA89775&Dopt=g</url>
</refsequence-feature>
- <refsequence-feature>
<name_type>SGD</name_type>
<name_value>YMR277W</name_value>
<description>TFIIF interacting component of CTD phosphatase</description>
<url>http://genome-www.stanford.edu/cgi-bin/dbrun/SacchDB?find+Sequence+%22YMR277W%22</url>
</refsequence-feature>
- <refsequence-feature>
<name_type>GENE_SYMBOL</name_type>
<name_value>FCP1</name_value>
</refsequence-feature>
- <refsequence-feature>
<name_type>GI</name_type>
<name_value>825543</name_value>
</refsequence-feature>
- <refsequence-feature>
<name_type>ORF_STATUS</name_type>
<name_value>No change</name_value>
</refsequence-feature>
- <refsequence-feature>
<name_type>ORF_TYPE</name_type>
<name_value>Verified</name_value>
</refsequence-feature>
</refsequence>
- <refsequence>
<refsequence-id>822</refsequence-id>
<refsequence-species>2</refsequence-species>
<refsequence-cds-start>1</refsequence-cds-start>
<refsequence-cds-stop>2241</refsequence-cds-stop>
<refsequence-chromosome>15</refsequence-chromosome>
<refsequence-sequence>ATGTCGTATAAATTTATTACCAAGAACAAAAAATACACCCCGATGTCTTC
CCCAGGGAATTCAGGCGTGGCTATAGATTCTACCGTTCTGAAAGCAATTGAATTAGG
AACGAGGCTTTTTAAATCTGGAGAATATTTGCAAGCGAAAAGGATATTCACAAATGC

TTTGAGAGTATGTGATTCTTATTCTCAGGAGCAGATTATGCGCATTAGGAACGCGTA
TCAATTAGATACTGCGAGACCGGATAACAAACGATTATATCATCCTAGATACATAAA
GATATTGGATAACATCTGTGCGTGCTACGAAAAATTGAATGATTTGAAATCTTGTTTG
GACGTATCACAAAGGTTACTTAAGTTAGAACCAGGTAATATCAAATGCTATATACGA
TGTACAAGGACGTTGATAAAATTAAAAGACTGGAAAAGGGCATATAAAACATGTTCT
CGTGGACTGCAATTATGCAATAACGATAGCAATCATCTAAGACAACAAAAACAATTC
ATTAAAAATAACATGGTTCAAAAACAGGACGGCAAGAGAAGTTATATAGATCCATTA
GAGGAGACCAAAATAGCAAAAAAAAAAAAGAATAATAATGTTCTAGAATCGTTACC
AAAGAAGAAGATTAAAGGTAGTACCAAGAAAACTGATTTAGTTGGCAATTTGCCGAT
AGAAATACTACCTATCATATTCCAAAGGTTTACCACTAAGGAGCTTGTTACGTTGTCG
CTAGTTTGTAACAAATGGAGGGACAAAATTTTGTATCATTTAGACTGCTTCCAAGAAT
TCAACTTGGCACCAATAAATTTTAAAAATTTTGTAAAGTTTATGGATTTTTTACAACA
GAATTTCACCAGGACGTATAGAAAATATATTCTTTCACAAGTAAAAGTCAGTTCTAG
AATCACTTCGGAAGAGTTACGAATAACTCAATTATTATTCAGTAAAATGCCGAAATG
TATAAACATCGAACGGCTTATACTGTCAATGCCAACACTAACTACAACACAAATCTTT
AAGTTAATGGTTAGAGGAGGCACAGATTTTTTTACAAGGCTGCTGGAATTATCTCTC
ATGATAACTTATAGGCCTGATAAACAACATGAATTGGAGATACTGCAGACTTGTCCT
CTGTTGAAAAAAATAGAATTAATTTTTGTCAATTCACTGGTACCAATTTTCGACGGAA
ATAATAGCGTTGGGAGGGATGGAAGTTTCAATGTGATGGCCCGTCATACTAATATGC
AGATATCCACTGCGGATAATGATGAACAGGGAATAGTAGAAGAAAAGGTGATTTAT
AGTGAGCTAGAGAAGATAACATTGATATGTGATAAGAAGAAAATTAAGAATTTTCCA
CTTTGTCGTGCTCTATTAAGAGGCCAGTTTCCTCTATTGCAGAAACTGACGATAACTG
GTGTAACATTTCCTATGAATAATCAAGACATAATGAATTTTCAATGGCTATTGAACTT
CCCAGACCTAAAAGAACTATGGATAGAAGATAACGACAACTGTGAACTCAGTAAGTT
TTTGCAATTGTTAAAATTTTCGAATGTCTGGAAAAACTTGGAAAAGTTAACCTTTAGA
GAGAACAAGCTATATCCAATTGTTAATTTGGATGAAGATCAGCCTGTTACGAATGAC
GACGAGGTGCCATCCATGCTATTTTACAAGGAAAATCTCCAAAACCTGGAAAAGTTG
GATCTAATGGGGACATCGATAAGTGGTTCTGCGTTGACTAGATTATGCGAGCAGGAA
TACTTGGATGGTCGGAAATTAAGAAGTTTAAACATTGGGAATTGCCCCAACATCCAG
TTCCCAAATAATCACGCTCATACAGCAAGGATGATACTGGATGTGAATGCAGTTTTG
AAGAGACTCTCCAAGTTGGAAGAAATAAATTTATCGCATCTCAGCTCCTTGAATGAT
AGTACGATGAAGTCATTCATTATTAATGTACCATTTTTGGAAAATTTAAAGAGGCTTG
ACATTTCGCACAATTTTGAAATTACAGGAATATCAATATATGAATTTCTAAAAAAATT
TCAGATGGATCATGACAACGAAGCGGGAGGTCAACCACTGGCATATTTAAATATTGA
CGGATGTTCCCAAGTGTCTCATATCACAGTTAATATGATCCGAGCGCAGAACTTAGTT
ACTCAAGTGGATTGTGTTTATGAAAGAGATGTATGGAGAAAATTTGGAATTAATTCA
TATTCATACTCATAG</refsequence-sequence>
- <refsequence-feature>
<name_type>SGD</name_type>
<name_value>YOR080W</name_value>
<url>http://genome-www.stanford.edu/cgi-bin/dbrun/SacchDB?find+Sequence+%22YOR080W%22</url>
</refsequence-feature>
- <refsequence-feature>
<name_type>GI</name_type>
<name_value>1420241</name_value>
</refsequence-feature>
- <refsequence-feature>
<name_type>GENBANK_ACCESSION</name_type>
<name_value>CAA99273</name_value>
<url>http://www3.ncbi.nlm.nih.gov/htbin-post/Entrez/query?db=p&form=6&uid=CAA99273&Dopt=g</url>

</refsequence-feature>
- <refsequence-feature>
<name_type>ORF_STATUS</name_type>
<name_value>No change</name_value>
</refsequence-feature>
- <refsequence-feature>
<name_type>ORF_TYPE</name_type>
<name_value>Verified</name_value>
</refsequence-feature>
</refsequence>
- <refsequence>
<refsequence-id>823</refsequence-id>
<refsequence-species>2</refsequence-species>
<refsequence-cds-start>1</refsequence-cds-start>
<refsequence-cds-stop>2250</refsequence-cds-stop>
<refsequence-chromosome>15</refsequence-chromosome>
<refsequence-
sequence>ATGTCTAATACCTTGCCAGTAACAGAATTTCTTTTATCCAAATACTACGAA
CTTTCCAACACTCCTGCCACAGACTCATCATCGCTCTTCAAATGGTTATACCACAAGA
CTCTCTCCCGTAAACAGCTGCTCATATCCGATTTGTCTTCCCAGAAAAAACACGCCAT
CTCCTACGATCAATGGAATGACATAGCGTCGCGATTAGACGACTTAACGGGACTTTC
CGAATGGAAAACAATCGACGAGTCCTCGTTGTATAACTATAAGCTGCTGCAAGACTT
GACCATCCGTATGCGCCATTTGAGAACCACTCACGACTACCACCGCCTGCTTTATCTG
ATTAGAACTAAGTGGGTCCGTAATCTTGGCAACATGAATAACGTTAATCTTTACAGG
CACTCGCATACCGGCACAAAACAAATTATACATGACTACCTAGAAGAGTCTCAAGCA
GTGCTCACCGCGCTGATCCATCAGTCAAATATGAACGATCACTATCTCCTCGGTATTT
TGCAACAGACGAGAAGAAACATAGGGCGCACGGCTCTCGTGCTTAGCGGTGGGAGC
ACTTTCGGCCTTTTCCACATTGGTGTTCTTGCCGCCCTTTTTGAATCGGACCTGATGCC
TAAGGTGATCAGCGGTAGCAGTGCTGGCGCCATTGTTGCCAGCATATTTTGCGTCCA
CACGACCCAGGAAATTCCCTCCTTGTTGACCAATGTACTTAATATGGAGTTTAACATC
TTCAATGACGACAATTCCAAATCTCCCAACGAAAACTTACTAATCAAGATATCGAGG
TTCTGCCAAAACGGTACCTGGTTCAATAATCAACCTTTGATTAACACAATGCTTTCGT
TTTTAGGAAACTTGACCTTTAGGGAAGCCTACAACAAGACTGGAAAAATCCTGAATA
TCACAGTCTCGCCTGCTTCCATATACGAACAGCCGAAACTACTAAACAATTTAACCGC
TCCAAACGTTCTCATCTGGTCTGCCGTATGCGCATCTTGTTCTCTACCTGGAGTTTTCC
CCTCCACGCCGCTATTCGAGAAAGATCCTCACACTGGAAAGATTAAAGAGTGGGGGG
CAACAAATTTACATTTATCAAACATGAAATTCATGGACGGATCTGTAGATAATGACA
TGCCCATTTCTCGTCTTTCTGAAATGTTCAATGTCGACCACATTATCGCCTGCCAGGT
AAATATACATGTCTTTCCCCTGTTAAAGTTTTCAAACACTTGCGTTGGGGGTGAAATT
GAAAAGGAAATTACCGCCCGTTTCAGAAACCAAGTAACAAAGATCTTCAAATTTTTT
TCCGACGAAACTATTCATTTTTTAGACATCCTAAAGGAGCTTGAGTTCCATCCCTATT
TGATGACCAAATTGAAACACCTTTTTTTACAACAATACTCTGGCAATGTCACAATTTT
ACCCGATCTATCAATGGTTGGTCAATTCCACGAAGTATTGAAGAACCCATCTCAACTT
TTCCTATTGCACCAAACTACTTTAGGTGCAAGAGCTACTTGGCCGAAAATTTCCATGA
TTCAAAATAACTGTGGCCAAGAATTCGCCTTGGATAAGGCCATCACATTCCTAAAGG
AGAAAATAATAATCTCTTCGTCAATAAAAAACCCTTTACAATTCTACCAACCTCGATT
CAGTGAGCAAATCAAATCTCTTTCCATAATGGATGCTGACTTGCCGGGAGTTGACTT
GGAAGAATCCTCCTCCAATTCACTATCAATTATCAAGTCTCCCAACAAAACAGCGGCA
CCGGGAAGATTTCCTCTTCAGCCATTGCCTTCTCCATCTTCTACCTTCAACAAGAGGA
AAATGGATATGTTATCGCCTTCTCCATCGCCTTCTACATCTCCACAACGTTCAAAATC
TTCATTCACGCAGCAGGGTACAAGGCAGAAGGCAAATTCTTTATCGTTTGCCATCGG
TGCATCTAGTTTACGGCTAAAGAAATCACCATTGAAGGTTCCATCACGACCTCAATTC

AAAAAAAGATCTTCTTATTATAATCAAAATATGTCGGCAGAGATGAGGAAAAATAGA
AAAAAATCTGGAACAATTTCCTCTTATGATGTTCAAACAAACTCAGAAGATTTTCCCA
TACCGGCTATTGAAAACGGTTCATTTGATAACACTTTATTCAATCCAAGCAGGTTCCC
CATGGACGCTATGTCGGCTGCCACAAACGACAATTTCATGAACAATTCAGACATTTTT
CAAAATTGA</refsequence-sequence>
- <refsequence-feature>
<name_type>SGD</name_type>
<name_value>YOR081C</name_value>
<url>http://genome-www.stanford.edu/cgi-bin/dbrun/SacchDB?find+Sequence+%22YOR081C%22</url>
</refsequence-feature>
- <refsequence-feature>
<name_type>GI</name_type>
<name_value>1420243</name_value>
</refsequence-feature>
- <refsequence-feature>
<name_type>GENBANK_ACCESSION</name_type>
<name_value>CAA99274</name_value>
<url>http://www3.ncbi.nlm.nih.gov/htbin-post/Entrez/query?db=p&form=6&uid=CAA99274&Dopt=g</url>
</refsequence-feature>
- <refsequence-feature>
<name_type>ORF_TYPE</name_type>
<name_value>Uncharacterized</name_value>
</refsequence-feature>
- <refsequence-feature>
<name_type>ORF_STATUS</name_type>
<name_value>No change</name_value>
</refsequence-feature>
</refsequence>
- <refsequence>
<refsequence-id>749</refsequence-id>
<refsequence-species>2</refsequence-species>
<refsequence-cds-start>1</refsequence-cds-start>
<refsequence-cds-stop>2274</refsequence-cds-stop>
<refsequence-chromosome>15</refsequence-chromosome>
<refsequence-sequence>ATGATGCGTGGTTTCAAGCAAAGATTAATAAAGAAGACCACCGGGTCTTC
TTCTTCTTCAAGCAGTAAAAAGAAGGACAAAGAGAAGGAAAAAGAAAAAAGTTCCA
CTACCTCATCCACATCAAAGAAGCCCGCTTCGGCTAGTAGCTCTTCCCACGGGACTAC
TCACAGTTCTGCCAGCAGTACCGGATCAAAGTCTACAACTGAGAAGGGCAAGCAATC
TGGTAGTGTTCCCTCGCAAGGGAAGCATCATAGTAGCTCTACATCAAAAACAAAAAC
AGCGACGACCCCTTCTTCCAGCAGCAGTAGCAGTAGAAGTTCAAGTGTCAGTCGCTC
CGGTTCAAGCTCCACAAAGAAAACAAGTTCAAGAAAAGGACAAGAACAGTCCAAAC
AATCGCAACAGCCATCACAATCTCAAAAGCAAGGATCTTCTTCATCATCTGCCGCTAT
AATGAACCCCACTCCAGTACTCACTGTTACTAAGGACGACAAAAGCACTTCTGGTGA
AGATCATGCACATCCTACTTTGCTGGGGGCAGTATCCGCTGTTCCATCATCTCCCATT
TCAAATGCTTCAGGTACAGCAGTTTCTTCTGATGTAGAAAATGGTAATAGTAATAAT
AACAATATGAATATTAATACTAGTAATACTCAGGATGCAAACCACGCCTCCTCACAA
AGTATCGACATTCCGAGATCATCACACTCATTTGAGAGACTACCAACACCCACAAAA
CTTAACCCTGACACAGATTTAGAGTTAATTAAGACTCCCCAACGTCATTCTTCATCTA

GATTCGAGCCATCTAGATATACGCCATTAACGAAATTGCCAAATTTTAATGAAGTTTC
TCCTGAAGAAAGAATCCCTTTGTTCATTGCCAAAGTTGACCAGTGTAACACTATGTTT
GACTTTAATGATCCAAGTTTTGACATTCAAGGTAAAGAGATTAAAAGAAGCACCTTA
GATGAGCTAATAGAATTCCTTGTAACAAATAGGTTCACTTACACGAATGAGATGTAC
GCTCATGTGGTGAACATGTTCAAAATCAATCTGTTTAGACCTATTCCACCACCAGTAA
ATCCAGTTGGTGACATTTATGACCCAGATGAAGATGAACCTGTTAACGAACTAGCCT
GGCCTCATATGCAAGCTGTTTACGAATTCTTTTTAAGGTTTGTGGAAAGTCCTGATTT
CAATCATCAGATTGCTAAACAATATATTGATCAGGACTTTATTTTAAAGTTACTGGAA
TTATTTGATAGCGAAGATATCAGAGAAAGAGACTGTTTGAAAACGACACTGCATAGA
ATATATGGGAAGTTCTTATCATTAAGAAGCTTTATTCGTCGGTCGATGAATAATATTT
TTTTGCAATTTATTTATGAGACTGAGAAGTTTAACGGTGTGGCAGAATTGTTAGAAA
TTTTGGGTTCCATAATTAATGGATTTGCACTTCCATTAAAGGAAGAGCACAAGGTTTT
CTTGGTGAGGATATTGATACCATTACACAAGGTCCGTTGTTTATCATTATACCACCCT
CAGTTGGCTTACTGTATCGTTCAATTTCTCGAAAAAGATCCTTTATTAACCGAAGAGG
TAGTTATGGGCTTACTGCGTTATTGGCCAAAAATAAATTCCACAAAAGAGATAATGT
TTCTAAATGAAATCGAGGATATTTTTGAAGTGATCGAACCGCTGGAATTTATTAAAG
TAGAAGTTCCGTTATTTGTTCAATTAGCTAAGTGTATTTCTTCTCCACATTTCCAAGTG
GCGGAAAAGGTTTTAAGTTATTGGAATAATGAATATTTCTTAAACTTATGTATCGAA
AATGCCGAAGTCATCCTACCCATTATATTTCCTGCATTATATGAATTAACTTCTCAGTT
AGAGCTAGATACAGCAAATGGCGAAGATAGCATTTCAGACCCTTACATGCTTGTTGA
GCAAGCAATCAATTCTGGTTCGTGGAATAGGGCAATTCATGCTATGGCATTCAAGGC
ATTGAAAATTTTTCTGGAAACAAACCCAGTATTGTACGAAAACTGTAATGCATTGTAC
TTATCAAGTGTAAAAGAAACTCAACAGCGTAAGGTGCAACGTGAAGAAAATTGGAG
CAAACTTGAAGAATATGTAAAAAATCTAAGGATTAACAATGATAAGGACCAATACAC
AATCAAAAACCCAGAATTAAGAAACAGTTTCAACACAGCAAGTGAGAATAACACATT
AAATGAAGAGAACGAAAATGATTGTGACAGCGAGATACAGTGA</refsequence-sequence>
- <refsequence-feature>
<name_type>SGD</name_type>
<name_value>YOR014W</name_value>
<description>B-type regulatory subunit of protein phosphatase 2A (PP2A)</description>
<url>http://genome-www.stanford.edu/cgi-bin/dbrun/SacchDB?find+Sequence+%22YOR014W%22</url>
</refsequence-feature>
- <refsequence-feature>
<name_type>GENE_SYMBOL</name_type>
<name_value>RTS1</name_value>
</refsequence-feature>
- <refsequence-feature>
<name_type>GI</name_type>
<name_value>1420113</name_value>
</refsequence-feature>
- <refsequence-feature>
<name_type>GENBANK_ACCESSION</name_type>
<name_value>CAA99203</name_value>
<url>http://www3.ncbi.nlm.nih.gov/htbin-post/Entrez/query?db=p&form=6&uid=CAA99203&Dopt=g</url>
</refsequence-feature>
- <refsequence-feature>
<name_type>ORF_TYPE</name_type>

<name_value>**Verified**</name_value>
</refsequence-feature>
- <refsequence-feature>
<name_type>**ORF_STATUS**</name_type>
<name_value>**No change**</name_value>
</refsequence-feature>
</refsequence>
- <refsequence>
<refsequence-id>**4867**</refsequence-id>
<refsequence-species>**2**</refsequence-species>
<refsequence-cds-start>**1**</refsequence-cds-start>
<refsequence-cds-stop>**2286**</refsequence-cds-stop>
<refsequence-chromosome>**15**</refsequence-chromosome>
<refsequence-
sequence>**ATGGAACACCAAGATAGTTCGCCACCTAGATTCAGGAACTCTGGCTCTAA
TAGAGTCACTGTGTATAACGGTACTACCCTGCCTACGATGCCCAAGAGCGCCACACC
AACATCGAGCTCAACAACAGTTACTACGCATTTGCAAAATATTAAGGAGGAGGAAAC
AAACGATGACGAACTCACCCAAGTGGATCGTTCATCTCCTCGTGTTTTGGGGAGGAT
CTCCTCTACGTCCTCATCTTCATCCAATATTGATTTGCGCGATAACTTGGACATGTTAC
ACGAAATAGAAAAATCAAATACTAATATTTCTTTATCAGCTCCTAATTTGCATGAGGA
ACTGGGCGTTCTCAGCGATAAAGGTAACAGTAAAGAAGAATTGGCTTTGTTACCACC
TTTACCTCACACAGGAGAAATGGAAATCACTCCACAATTTGATATCAACGAGGCTAT
TTTTGAACGAGATGACATCAGCCACTCTTCAAGGCTAGAACCAGATGACGTGTTAAC
AAAGTTAGCGAACTCTACTCGAGATGCTACGGGTGAAGATCAGGGGTTTGTTGTCAT
GACTCATGGCCACGATGCGTCGACAAACGACGATTCGCAATTAAGCGCTACTATTCT
TGACAATCAGACGTCATTTGATCTTTCTAAAGCTCTAGAGATGACTAGCCATTCAAAT
ATTTCTAATATTATTAATAGTTCCGGTTCTGAGGGAAGACGTTCAAGGACACCGGTA
AGCAATTCCACTCTGAAACCAAATTTATCATCTCCTGAAAGCGCAGAACGTGAAGCA
AACACGACTTCGTCCTCCTCTACGTCGGATCACGGGGCAACAATGCAATATGATCCC
AAGAAAATAATAACTCCAATTCCTGTTTTACCTTCCTCTGTCCGTGAACAGCAACAGA
ACAATGCACCTTTGAGAGAAAGAAGCAGATCTAATTCTAGTGCACTGGCATCTACAC
TGAGAGATACGATCATTTCAGGACTGCCTCAAAATATTAATTCTGTAGAAAGAAAGT
TATCAAGGAAGAGTAACAGGAGTAGGAAGAATACGGTGACTTTTGAAGATCGTCTTC
AGAAATTGCCACCCTTAAGCACCCAAATTTCGAACCAGTACGCCAAGGTAGCACCAG
CTGAGAACAATATCGCCTTACACTTTCATAACTTACCTACACCAGTATCAAACACTCA
AACGCCTGTTACGTTTCAGTCTGAATCCGGACTGACAGGGGGAGAAAAGAAAATGCC
TTTTTTGAGAAGAGCATCTAGTGCCCTATTAAGAAAGACATCTGCCAAAAATTGCTCC
AATTTAACCAGAACAAATACACCTACTTTATCGACATCCTCAACATTTGAGTCAGATC
TAAATGCTCGGCAGCCCATGCTAATTCGACGATCTTCCACTATTGATAATAAACTACC
TAGGAGGCAGCTTTCGTGCTCGAAGCTTTATTCGCGCCTCAATTCGGACAGCAAGTT
TGCGAATAGCAGTCGAGCTTCGGAGGAGGTCTTAGTGTCCACTCCAAATGACACAGA
ACACGTCTACAGAAAGACATCTCTAGGTTCTAAGATAAAGAGAGGTTTTACTAGAAT
ATTGAGCGACAGTAATAATAGTAAGGAAATTCTCACTTTATCACCCAAATCTATGGT
GACTACGGGGCCTACAGAATTGTCGTTTTCCTCTTTATCAACCGTGGGAGGACATCC
GACAACGCCAGTCTCAAAAGAAAATGATCGAGTTTCAATAGATGGCGTGAGTACATT
TAATCGAGCATCAACATCTCTTCCAGAATCATCAACAGACGACATCTCTCCACTACGC
GAAGAAGGTAAGATTAATGTTCCTAAAAGAACGTCAAGTAGAAAGATACTGTCTAAA
AATTCAAGCAAAAAAAATGTACTGCCTGAACAGCAAACAAAGCCAAGTGAAATATAT
CTGGATAAAGAAGCCTTACAAAGTTTTGTTCCCGTACTCTCTGTCACAGAGGGTACTC
ATCGCATCAATCGCTCGTCGTTACAAACGCAATCTACCATCGGATTATGCATTACCAA
TTTAAGAAACAAAGAAGGCATGAAGCTCAATGCCAAGGAATACGTGGAAATCCTGG
CTCAGCAGCAACGCAAGGAAGATGAAAGGTATGCTGTTTTGGAAAGAAAATTTGCAT**

CTTGTAGATGGTGCAGTGATAAGGACCTGCAGTATTTGAAAAAGAAACGAATTTCCA
TGAATAAGATATGGTCTGATTATGTCCGATTTTACCGTGGAAAGTTGAACAACCCAT
GA</refsequence-sequence>
- <refsequence-feature>
<name_type>GENBANK_ACCESSION</name_type>
<name_value>CAA99038</name_value>
<url>http://www3.ncbi.nlm.nih.gov/htbin-
post/Entrez/query?db=p&form=6&uid=CAA99038&Dopt=g</url>
</refsequence-feature>
- <refsequence-feature>
<name_type>SGD</name_type>
<name_value>YOL036W</name_value>
<url>http://genome-www.stanford.edu/cgi-
bin/dbrun/SacchDB?find+Sequence+%22YOL036W%22</url>
</refsequence-feature>
- <refsequence-feature>
<name_type>GI</name_type>
<name_value>1419830</name_value>
</refsequence-feature>
- <refsequence-feature>
<name_type>ORF_STATUS</name_type>
<name_value>No change</name_value>
</refsequence-feature>
- <refsequence-feature>
<name_type>ORF_TYPE</name_type>
<name_value>Uncharacterized</name_value>
</refsequence-feature>
</refsequence>
- <refsequence>
<refsequence-id>858</refsequence-id>
<refsequence-species>2</refsequence-species>
<refsequence-cds-start>1</refsequence-cds-start>
<refsequence-cds-stop>2286</refsequence-cds-stop>
<refsequence-chromosome>15</refsequence-chromosome>
<refsequence-
sequence>ATGAATTTTTCCAGCATTTTCAAATCTATTTCGAATTTCCAGTTCCCATAC
ACCATAGAGGAAACTGCAATCACTGAAACCGCCCTTTGGCAATGTTTCGATGGTACA
AGGAAGGCAGATTCCTTGCCAGTAACAGTTTTTAAGGCAAAAAGATCTCCAGAAAAT
GAATCTTTAATTTTAAATGCTGTACACAAGAGCAAAATTTTGAAGATTCCAGGACTTT
GTACGGTGCTGGAGACATTTGATTCCGACCCCCAATCCACATTTATTGTTACCGAACG
AGTAGTTCCATTTCCTTGGGATAATCTAGGCTCTTTATCTCAAAACAAATTTGGTGTT
GAATTAGGAATATCACAATTATTGGCAACTTTAGGATTTTTAAAAAACTTTGTCCTTG
GTACGCTTTCTAAAGATTCTGTTTTTATCAATATTAAGGGAGAGTGGGTACTATTTGG
GCTAGAACTTTGTTCAAGCAAAGAAGGATTAAGTGCGTTTGAATTTGCCAGTAGAGC
GAGGTCATATTACAACATAATTGGCTCACAGTTGCCCTGTGAAGATCCGAATACAAT
TGATTCAATGGGGTTAGGATTGTTGATAAAGAGCCTTATGGCTCCTTCTTGTCTGCCC
AAAGATTGGATTGTCAATGTAAATATGATTTCAGATGGAAAGATAACAATCGAAAAC
TTCAGAAAAAGGCTAGAAAATACAGAAACTTGGCGTTCTAATCCTTTGATAAACTTTT
ATCAAGAATTGAGGGAGTTGCATATAAAGGACCCACAGGGAAAATTGGTTGTGATGT
CAAACTTGGAAAATTTGTATTTAGAGTCAAGAGAGATTTTCCGTAATTTAACGCCTG
GAATGATAGAAAATTTTATTATTCCAGAGCTTTGTGAAATTATAAAATTATTAATGAC

ACAAAGCATCAGTAGTGCTGCCAGCCCTATAGGCATGAACTTCAACGCATCACATAA
ACTAGTACCATTCTTAGCGATTGTTTTAGACCTAACTTCTGAAACGAACACCTTTCCA
GTTGGTTTCAACGACCTTATCACCCAAAGTTTTAAACTGCCAGATAGGCAAGTAAGA
TTTCTTTTACTAATATACCTACCCAAATTAATAGGTCCATTGAGCAAATCAGAGATCT
CCAGTAGGATATATCCACATTTTATCCAAGGTCTGACCGACTCTGATGCCACTCTTAG
ATTACAAACACTGAAGACAATTCCATGCATTGTGTCATGTTTAACAGAGAGACAACT
GAATAATGAGCTACTGAGGTTTCTCGCAAAAACACAGGTTGATTCTGACGTTGAAAT
CCGAACATGGACAGTCATCATAATAAGTAAAATCTCAACCATATTATCAACGTCAGTT
GGTAATCGCTCGAATATTTTAGCTACGGCGTTCACAAAATCTTTGAAAGATCCTCAAG
TAAAACCAAGATTGGCTGCTCTTTATGGGCTTGAGAAGTCGATTGAGCTGTTTGATG
TGAACACAATCGCCAACAAAATTTTAACAGTCATTGCCCCTGGTTTATTAGATAAAAG
TCCCATAGTAAGAGGCAGAGCCAAAATCCTGTTCGAAGAATATCTGGAAAAATTGGA
AAAAGAAGCCCAACTTATTCAAACAAACGATAGTACTGCAGATTCGGAAGATGTGAA
AGATATTGATTTTGAGAATTACGGTTGTGATGAAGAGGATATGAATAAAGAAGACAA
TTTGTTAGCCGCACAATTTTTAAATAATTTACGTTTAAATTCTCCCTCAGCAACAACA
CCAAGTAACATTACCGAGAGCGAAATTGATTCCGCCCAGGATGGAAGTGGATGGGA
CGACCTCAGCGATACCGATGGCTTTATTACAAATGGTACCACAGAATCCTTCGATGA
AACAACAAACCCTGTAACAACCGCAAGCACCCCAAAATTATTCGGAAAACCTATTAA
AATTAATAAAAGTTGGAATGATGAGTTGAATGATGATGGCTGGATTCAAGATGAAAG
CGGCCCATCAAAGGTGCCTCAAAAACACACAAGGCCACAAAATTCAACGTTGGCAAA
ATCCATCGCTCCTAGCTCAAGGCTTTCTATCAAGAAGAAGAAAACAACGATCCTAGC
ACCAAGAAACATTGCTAGTAACTCTACTGTTACCACCAAATCGTCACTGTCCAATAAA
ACTGCAAGAAGTAAGCCTATAAGTAGTATCCGCGGCTCGGTAACCAAGAAAGGAAA
TGTTGACGGCTGGGATGATGATGGGGATTCAGACTCCTGGGATACGAATTGGTGA</
refsequence-sequence>
- <refsequence-feature>
<name_type>SGD</name_type>
<name_value>YOR112W</name_value>
<url>http://genome-www.stanford.edu/cgi-
bin/dbrun/SacchDB?find+Sequence+%22YOR112W%22</url>
</refsequence-feature>
- <refsequence-feature>
<name_type>GI</name_type>
<name_value>1420301</name_value>
</refsequence-feature>
- <refsequence-feature>
<name_type>GENBANK_ACCESSION</name_type>
<name_value>CAA99310</name_value>
<url>http://www3.ncbi.nlm.nih.gov/htbin-
post/Entrez/query?db=p&form=6&uid=CAA99310&Dopt=g</url>
</refsequence-feature>
- <refsequence-feature>
<name_type>ORF_TYPE</name_type>
<name_value>Uncharacterized</name_value>
</refsequence-feature>
- <refsequence-feature>
<name_type>ORF_STATUS</name_type>
<name_value>No change</name_value>
</refsequence-feature>
</refsequence>
- <refsequence>

<refsequence-id>4610</refsequence-id>
<refsequence-species>2</refsequence-species>
<refsequence-cds-start>1</refsequence-cds-start>
<refsequence-cds-stop>2304</refsequence-cds-stop>
<refsequence-chromosome>14</refsequence-chromosome>
<refsequence-sequence>ATGGCAAAAAGGCATAGTCATTATCAAGGAAGCAGACGTAGGCATGCAC
GTGGTAGCAATTCCAAGAAAGCTGGCAGGGGGAATGCTAAAGGCATACAAGGCAGA
AAGATAAAGAAAAAACCTACTCCCACTAACAGTTGGCATAACTCTTCTATACCACTA
GGAGAAGGTGATCTTGATGATGTAGGCGCCGACTTCAACCCAGGAAGAGCATTCATA
TCACCAAAAACTATAGAAGACTATTATTTTGGTCGCGATGCCAAGAGCCGTTCTATG
AAAATGGGTGGTTTAAGACCAGGTAATCGATATGATTCTTCAACTGACCTACAAGCA
GGAAGGGCAGCATTCCGCAAAAGACCTATGCAATTTGTAAAAGCTAAGGAAGTTTAC
GATCCATCCCACAATATGATTCAAAAACTGAGAGCAAAGAACGAAACAAAGAACAGT
GAAGAAATTGTCGAAAGGGAAGCAGACGTTTTTGAAGAACCAGGAAAGATGACTTC
TGATGTTGAATATATTAACAATGAAGACAGTGAAAACGAGGATGATGATAGTCAGA
ATTCACCCTCGACGGACCACTCGCTGTCTTCGAATGAAAGCAAAGTTGAAGATGGTG
ACCTGTTTTTTGTAGATGAAGAGGCTCAGCAATCGCCGGATCTAACCAAAATAAAAA
GAGTATGTATCGAGGAAATCGCAAGGCCAAGAGAAGTTGCGATAGAGTTCGATCCT
ATCTTAACAATTGGAAAGGTTGAACTTAGCGTTTCCGAAGGTAACGAAAGCAAGGAA
ATTAGTGTTGACGTTCCAAATAAGGGTAATAAGACATATCATCCATTTGCTGGTTACA
TTTCCAACGTATTACATGGTATGCATACTAGCGACTCGGACAATGATGAACTAGACT
ACGAAATAGAAACAGAGAATAATAGTGAACCTTTATACGAATCCTCAGCAAGTTCTG
AGGTTGATCAAGGGTTTAATTATGTTGGCCAACGTCACAATTCCCGAGCTGACAACA
ATCTTTTACCAAGCCCTAGTCCGCAGTTGACAGAAGATATAAAATGTCTTTCAATAAA
TGGTACCAAAACATTCGAAGGCAATAATGATAATCTGCCATCTCCTGCTAGCGAAGA
GTTAGAATTTGGATTCAAAGAAGAAGATTTTGTCATAAATACTAATGACATAGTGGT
CTCCAATATAAGAATGGGCGGAGTAGACAATTCATATTATCTACGGTGTTACAGATT
ACTGGGAGATTACGACTTTCATTGGATTGACCAGGACTTACTCACAGACTTCGTTGTA
GACGAGTTAGGTCTTCCTGAAGATAGATTACCTGCATATTTGAATTTTATTAAAAATT
CTCTTATACCAAAAATTGAACCAGCTGAGCCAACCTACTCAGATATTCCGATTTCTGA
TTCGAGTGATGAAGGTGACAGCTATGAAGGCGACAGCTATGAAGATGATGAAGATA
TGGCCTCCTCGGTTGTGCATAGCGACATTGAAGAGGGTCTGGACGACCTAATTGCAT
ACACATTGAAACATGATACAGAAAGATTCAAAACATTTGAAACCAAGTCCTTGGAAA
CTAAGGGTAAAGGTAAGAAGAAGAAGCTACTCATCGATGACGCCTTAGCGCTGGAT
ACAGAAACGTTGGAGACGTTACAAAGTAAGTTTAGTAAACGCATAGAAACCAAAGC
AAAGAAAAGAAAAGCCAAAGAAGATTTCATTGATCAGGAGAATAGAAATTCGAACG
ATATGCTCAAGAAATACCCATATGGACTCCATATTCAAAATATTAAAGATGAATTTGA
GTCCTTTTTGTCTAGGAACAATGATAGACTGACTTTCCCTCCATTAGATCCTCATGGA
AACAAGACCGTAATGAAAATTGCGAAGCATTATAATATGAAGAGTTCAAAAATTGGG
AAAGCAAATCATACGTCGGTTGTTGTAGAAAAAATAAAGAAAACGAAATGGTCTTCT
CCGAATTATAGTCTCATTGACCAATTGATGAGGCAGAGGCCAGTATTCATGAGAATA
GATATAAGAAGACCGAGGGAGGAACAAGCTGCTTTCGAAAGAACTAAAACAATCAG
AGGGAAATTTCATGTTAAAGAAGGTGAGATTGTTGGTCAAAACGCTCCAGAGATTGG
AAACGAAAATATCGGTAGAAGAATGTTGGAAAAGCTTGGGTGGAAAAGTGGAGAGG
GCCTTGGTATTCAAGGCAATAAAGGTATAAGTGAACCAATTTTTGCCAAAATTAAAA
AGAATAGATCGGGTTTAAGACACAGTGAAAGTTAA</refsequence-sequence>
- <refsequence-feature>
<name_type>GENBANK_ACCESSION</name_type>
<name_value>CAA96127</name_value>
<url>http://www3.ncbi.nlm.nih.gov/htbin-post/Entrez/query?db=p&form=6&uid=CAA96127&Dopt=g</url>

</refsequence-feature>
- <refsequence-feature>
<name_type>**SGD**</name_type>
<name_value>**YNL224C**</name_value>
<url>**http://genome-www.stanford.edu/cgi-bin/dbrun/SacchDB?find+Sequence+%22YNL224C%22**</url>
</refsequence-feature>
- <refsequence-feature>
<name_type>**GI**</name_type>
<name_value>**1302245**</name_value>
</refsequence-feature>
- <refsequence-feature>
<name_type>**ORF_STATUS**</name_type>
<name_value>**No change**</name_value>
</refsequence-feature>
- <refsequence-feature>
<name_type>**ORF_TYPE**</name_type>
<name_value>**Uncharacterized**</name_value>
</refsequence-feature>
</refsequence>
</sequence-info>
