# Supplementary material for: A novel approach to sequence validating protein expression clones with automated decision making
Source: BMC Bioinformatics. 2007 Jun 13;8:198. doi: 10.1186/1471-2105-8-198 (PMC1914086; doi:10.1186/1471-2105-8-198)
Supplement: Additional file 5 — XML file with clone mapping information. Example of XML file contains clone mapping data for the plate 'YGS000374-1'. [file 1471-2105-8-198-S5.pdf]

```
<?xml version="1.0" encoding="ISO-8859-1" ?>
<!DOCTYPE web-app (View Source for full doctype...)>
- <!--
XML file with clone mapping information.
-->
- <!--
XML file contains clone mapping data for the plate 'YGS000374-1'.
-->
- <clone-collections>
- <clone-collection userid="1" name="YGS000374-1" project_id="3">
- <construct constructid="32316" format="1" cloningstrategyid="2"
    refsequenceid="4063">
  <sample sampleid="549322" cloneid="5272" well="A03" sampletype="ISOLATE" />
  <sample sampleid="549323" cloneid="5273" well="B03" sampletype="ISOLATE" />
  <sample sampleid="549324" cloneid="5274" well="C03" sampletype="ISOLATE" />
  <sample sampleid="549325" cloneid="5275" well="D03" sampletype="ISOLATE" />
  </construct>
- <construct constructid="32441" format="1" cloningstrategyid="2"
    refsequenceid="4202">
  <sample sampleid="549354" cloneid="5408" well="A07" sampletype="ISOLATE" />
  <sample sampleid="549355" cloneid="5409" well="B07" sampletype="ISOLATE" />
  <sample sampleid="549356" cloneid="5410" well="C07" sampletype="ISOLATE" />
  <sample sampleid="549357" cloneid="5411" well="D07" sampletype="ISOLATE" />
  </construct>
- <construct constructid="32488" format="1" cloningstrategyid="2"
    refsequenceid="4254">
  <sample sampleid="549358" cloneid="5452" well="E07" sampletype="ISOLATE" />
  <sample sampleid="549359" cloneid="5453" well="F07" sampletype="ISOLATE" />
  <sample sampleid="549360" cloneid="5454" well="G07" sampletype="ISOLATE" />
  <sample sampleid="549361" cloneid="5455" well="H07" sampletype="ISOLATE" />
  </construct>
- <construct constructid="32528" format="1" cloningstrategyid="2"
    refsequenceid="4298">
  <sample sampleid="549374" cloneid="5504" well="E09" sampletype="ISOLATE" />
  <sample sampleid="549375" cloneid="5505" well="F09" sampletype="ISOLATE" />
  <sample sampleid="549376" cloneid="5506" well="G09" sampletype="ISOLATE" />
  <sample sampleid="549377" cloneid="5507" well="H09" sampletype="ISOLATE" />
  </construct>
- <construct constructid="32685" format="1" cloningstrategyid="2"
    refsequenceid="4474">
  <sample sampleid="549338" cloneid="5627" well="A05" sampletype="ISOLATE" />
  <sample sampleid="549339" cloneid="5628" well="B05" sampletype="ISOLATE" />
  <sample sampleid="549340" cloneid="5629" well="C05" sampletype="ISOLATE" />
  <sample sampleid="549341" cloneid="5630" well="D05" sampletype="ISOLATE" />
  </construct>
- <construct constructid="32687" format="1" cloningstrategyid="2"
    refsequenceid="4476">
  <sample sampleid="549310" cloneid="5635" well="E01" sampletype="ISOLATE" />
  <sample sampleid="549311" cloneid="5636" well="F01" sampletype="ISOLATE" />
  <sample sampleid="549312" cloneid="5637" well="G01" sampletype="ISOLATE" />
```

<sample sampleid="549313" cloneid="5638" well="H01" sampletype="ISOLATE" />
</construct>
- <construct constructid="32709" format="1" cloningstrategyid="2" refsequenceid="4500">
<sample sampleid="549314" cloneid="5681" well="A02" sampletype="ISOLATE" />
<sample sampleid="549315" cloneid="5682" well="B02" sampletype="ISOLATE" />
<sample sampleid="549316" cloneid="5683" well="C02" sampletype="ISOLATE" />
<sample sampleid="549317" cloneid="5684" well="D02" sampletype="ISOLATE" />
</construct>
- <construct constructid="32808" format="1" cloningstrategyid="2" refsequenceid="4610">
<sample sampleid="549398" cloneid="5779" well="E12" sampletype="ISOLATE" />
<sample sampleid="549399" cloneid="5780" well="F12" sampletype="ISOLATE" />
<sample sampleid="549400" cloneid="5781" well="G12" sampletype="ISOLATE" />
<sample sampleid="549401" cloneid="5782" well="H12" sampletype="ISOLATE" />
</construct>
- <construct constructid="32970" format="1" cloningstrategyid="2" refsequenceid="4790">
<sample sampleid="549362" cloneid="5918" well="A08" sampletype="ISOLATE" />
<sample sampleid="549363" cloneid="5919" well="B08" sampletype="ISOLATE" />
<sample sampleid="549364" cloneid="5920" well="C08" sampletype="ISOLATE" />
<sample sampleid="549365" cloneid="5921" well="D08" sampletype="ISOLATE" />
</construct>
- <construct constructid="32986" format="1" cloningstrategyid="2" refsequenceid="4808">
<sample sampleid="549350" cloneid="5938" well="E06" sampletype="ISOLATE" />
<sample sampleid="549351" cloneid="5939" well="F06" sampletype="ISOLATE" />
<sample sampleid="549352" cloneid="5940" well="G06" sampletype="ISOLATE" />
<sample sampleid="549353" cloneid="5941" well="H06" sampletype="ISOLATE" />
</construct>
- <construct constructid="33014" format="1" cloningstrategyid="2" refsequenceid="4839">
<sample sampleid="549326" cloneid="6042" well="E03" sampletype="ISOLATE" />
<sample sampleid="549327" cloneid="6043" well="F03" sampletype="ISOLATE" />
<sample sampleid="549328" cloneid="6044" well="G03" sampletype="ISOLATE" />
<sample sampleid="549329" cloneid="6045" well="H03" sampletype="ISOLATE" />
</construct>
- <construct constructid="33021" format="1" cloningstrategyid="2" refsequenceid="4847">
<sample sampleid="549342" cloneid="6062" well="E05" sampletype="ISOLATE" />
<sample sampleid="549343" cloneid="6063" well="F05" sampletype="ISOLATE" />
<sample sampleid="549344" cloneid="0" well="G05" sampletype="EMPTY" />
<sample sampleid="549345" cloneid="6064" well="H05" sampletype="ISOLATE" />
</construct>
- <construct constructid="33027" format="1" cloningstrategyid="2" refsequenceid="4853">
<sample sampleid="549318" cloneid="6081" well="E02" sampletype="ISOLATE" />
<sample sampleid="549319" cloneid="6082" well="F02" sampletype="ISOLATE" />
<sample sampleid="549320" cloneid="6083" well="G02" sampletype="ISOLATE" />
<sample sampleid="549321" cloneid="6084" well="H02" sampletype="ISOLATE" />

</construct>
– <construct constructid="33040" format="1" cloningstrategyid="2" refsequenceid="4867">
<sample sampleid="549390" cloneid="6113" well="E11" sampletype="ISOLATE" />
<sample sampleid="549391" cloneid="6114" well="F11" sampletype="ISOLATE" />
<sample sampleid="549392" cloneid="6115" well="G11" sampletype="ISOLATE" />
<sample sampleid="549393" cloneid="6116" well="H11" sampletype="ISOLATE" />
</construct>
– <construct constructid="33062" format="1" cloningstrategyid="2" refsequenceid="4893">
<sample sampleid="549334" cloneid="6137" well="E04" sampletype="ISOLATE" />
<sample sampleid="549335" cloneid="6138" well="F04" sampletype="ISOLATE" />
<sample sampleid="549336" cloneid="6139" well="G04" sampletype="ISOLATE" />
<sample sampleid="549337" cloneid="6140" well="H04" sampletype="ISOLATE" />
</construct>
– <construct constructid="33188" format="1" cloningstrategyid="2" refsequenceid="749">
<sample sampleid="549386" cloneid="6221" well="A11" sampletype="ISOLATE" />
<sample sampleid="549387" cloneid="6222" well="B11" sampletype="ISOLATE" />
<sample sampleid="549388" cloneid="6223" well="C11" sampletype="ISOLATE" />
<sample sampleid="549389" cloneid="6224" well="D11" sampletype="ISOLATE" />
</construct>
– <construct constructid="33193" format="1" cloningstrategyid="2" refsequenceid="754">
<sample sampleid="549370" cloneid="6233" well="A09" sampletype="ISOLATE" />
<sample sampleid="549371" cloneid="6234" well="B09" sampletype="ISOLATE" />
<sample sampleid="549372" cloneid="6235" well="C09" sampletype="ISOLATE" />
<sample sampleid="549373" cloneid="6236" well="D09" sampletype="ISOLATE" />
</construct>
– <construct constructid="33196" format="1" cloningstrategyid="2" refsequenceid="758">
<sample sampleid="549346" cloneid="6237" well="A06" sampletype="ISOLATE" />
<sample sampleid="549347" cloneid="6238" well="B06" sampletype="ISOLATE" />
<sample sampleid="549348" cloneid="6239" well="C06" sampletype="ISOLATE" />
<sample sampleid="549349" cloneid="6240" well="D06" sampletype="ISOLATE" />
</construct>
– <construct constructid="33207" format="1" cloningstrategyid="2" refsequenceid="770">
<sample sampleid="549330" cloneid="6247" well="A04" sampletype="ISOLATE" />
<sample sampleid="549331" cloneid="6248" well="B04" sampletype="ISOLATE" />
<sample sampleid="549332" cloneid="6249" well="C04" sampletype="ISOLATE" />
<sample sampleid="549333" cloneid="6250" well="D04" sampletype="ISOLATE" />
</construct>
– <construct constructid="33254" format="1" cloningstrategyid="2" refsequenceid="822">
<sample sampleid="549378" cloneid="6319" well="A10" sampletype="ISOLATE" />
<sample sampleid="549379" cloneid="6320" well="B10" sampletype="ISOLATE" />
<sample sampleid="549380" cloneid="6321" well="C10" sampletype="ISOLATE" />
<sample sampleid="549381" cloneid="6322" well="D10" sampletype="ISOLATE" />
</construct>

– <construct constructid="33255" format="1" cloningstrategyid="2" refsequenceid="823">
<sample sampleid="549382" cloneid="6323" well="E10" sampletype="ISOLATE" />
<sample sampleid="549383" cloneid="6324" well="F10" sampletype="ISOLATE" />
<sample sampleid="549384" cloneid="6325" well="G10" sampletype="ISOLATE" />
<sample sampleid="549385" cloneid="6326" well="H10" sampletype="ISOLATE" />
</construct>
– <construct constructid="33286" format="1" cloningstrategyid="2" refsequenceid="858">
<sample sampleid="549394" cloneid="6378" well="A12" sampletype="ISOLATE" />
<sample sampleid="549395" cloneid="6379" well="B12" sampletype="ISOLATE" />
<sample sampleid="549396" cloneid="6380" well="C12" sampletype="ISOLATE" />
<sample sampleid="549397" cloneid="6381" well="D12" sampletype="ISOLATE" />
</construct>
– <construct constructid="33332" format="1" cloningstrategyid="2" refsequenceid="5039">
<sample sampleid="549366" cloneid="6440" well="E08" sampletype="ISOLATE" />
<sample sampleid="549367" cloneid="6441" well="F08" sampletype="ISOLATE" />
<sample sampleid="549368" cloneid="6442" well="G08" sampletype="ISOLATE" />
<sample sampleid="549369" cloneid="6443" well="H08" sampletype="ISOLATE" />
</construct>
<sample sampleid="549306" cloneid="0" well="A01" sampletype="CONTROL_POSITIVE" />
<sample sampleid="549307" cloneid="0" well="B01" sampletype="CONTROL_POSITIVE" />
<sample sampleid="549308" cloneid="0" well="C01" sampletype="CONTROL_POSITIVE" />
<sample sampleid="549309" cloneid="0" well="D01" sampletype="CONTROL_POSITIVE" />
</clone-collection>
</clone-collections>
