# Supplementary material for: A novel approach to sequence validating protein expression clones with automated decision making
Source: BMC Bioinformatics. 2007 Jun 13;8:198. doi: 10.1186/1471-2105-8-198 (PMC1914086; doi:10.1186/1471-2105-8-198)
Supplement: Additional file 6 — Help file. This is the pdf version of the online help files in the software. It includes both a tutorial and an overview of the software, as well as installation instructions. [file 1471-2105-8-198-S6.pdf]

ACE Tutorial

Automated Clone Evaluation (ACE) is a software suite designed to address the main goals of sequence verification for projects of any scale: (1) determine the sequence of each clone accurately; (2) identify if and where that sequence varies from the intended target sequence; (3) evaluate and annotate the polypeptide consequences of any variations; and (4) determine if these observed differences are acceptable based on user defined criteria. ACE was designed to eliminate bad clones as early as possible in the validation process, and minimize the need to produce additional sequencing primers.

An effective way to use this tutorial is to have both the tutorial and the ACE application open simultaneously and split on the computer screen. This split view will allow the user to follow the tutorial on the actual GUI or main window making the instructions far easier to comprehend. If the ACE application has not been referred for an extended period of time it will time out and need to be logged into again; you will get an 'expired message' when this happens.

Note: ACE GUI consists of a main window and a left hand drop-down menu. The main window displays forms according to user selection from the left-hand menu. Throughout this tutorial a notation like 'Process > Read Manipulation' means that user should select the 'Process' menu and select 'Read manipulation' item to get to the desired application.

ACE Tutorial

- Getting started
- Project and Analysis Settings and definitions
  - Cloning project settings
  - Analysis settings
- Defining new targets
  - Reference plate upload
- Operational Steps
  - Trace files upload
  - Sequencing data processing and clone evaluation
  - Reports

- Primer design and management
- Real time views
- Data clean-up

Getting started

Before any analysis can be performed the project and analysis settings and definitions must be pre-defined in ACE. Once these settings have been entered they do not have to be entered again unless projects or definitions change.

Cloning project settings

All definitions created in this section are shared among site users and site projects. The main window for each menu item provides the form to be filled in and submitted to create or update new definitions and/or summary tables that are to be used in all analysis.

- Related plates are organized into an ACE project. One project can be created and used for all plates in ACE. To **create a new project** select '*Cloning Project Settings > Project Definition*' and fill in project name and optional project description fields.
- To introduce **a new biological species** select *'Cloning Project Settings > Species Definition*': clones within a project can belong to different species.
- A clone has a target (reference) sequence against which it will be verified by ACE. The reference sequence can be annotated according to various annotation conventions. ACE can accept any names for these descriptors using *'Cloning Project Settings > Annotation Type*'. These **annotation types** will appear as parameter names for reference sequence submission. Examples of annotation types are GI, gene symbol, SGD etc.
- To introduce **new vector** definitions into ACE select '*Cloning Project Settings > Vector Information*' and submit an XML file with vector description(s) (several vectors can be submitted from one XML file). Each vector is described by its name, source, type (1 for destination vector, 0 for master vector), filename, file path. In addition a vector may have a set of features. Feature description contains: feature name ('recombination site'), feature property (1 for added, 0 for remain, -1 for lost when insert is integrated), feature description. These features will be displayed under '*View > Vectors*'.
- To introduce **a new sequencing primer (non gene-specific)** to ACE

select '*Cloning Project Settings > Sequencing Primer*' and provide primer name, sequence (if sequence is unknown enter 'NN'), Tm and select primer type from drop-down menu.

- For each vector only certain universal primers are used for end reads. To specify a relationship between a vector and universal primers use *'Cloning Project Settings > Link Vector with Sequencing Primer'*. Several primer-vector combinations can be created for the same vector (see screenshot for details).
- A linker is defined in ACE as part of the vector sequence located up-stream or down-stream of the insert and is considered important for clone function. Sequence verification of the linker is required for acceptance. To define a linker use '*Cloning Project Settings > Add Linker*'.
- Cloning strategy in ACE is a collection of the following parameters: sequencing vector, 5' and 3' linkers, start and stop codons. The application administrator defines possible start and stop codon values in an ACE Configuration file. To create a new cloning strategy use '*Cloning Project Settings > Add Cloning Strategy*'.

Analysis settings

Certain processing modules imbedded in ACE require analysis specifications (spec). All analysis specifications in this section are shared among site users of ACE and site projects.The table below list Specification Name (i.e. Clone acceptance criteria from the menu), Page (path to the main window to create or view spec), Feature (name of feature where spec is required). When creating a new spec user can view all specs created by user or all specs created at site to avoid duplication. New analysis settings can be added at any time and used during the analysis. Often a set of clones will be re-analyzed using multiple analysis settings. Click specification name for details.

Specification Name	Page	Feature
Clone Ranking	Analysis Settings > Clone Ranking	Isolate Ranker
Clone Acceptance Criteria	Analysis Settings > Clone Acceptance Criteria	Decision Tool<br>Quick Decision Tool<br>Isolate Ranker

Primer Design Specification	Analysis Settings > Primer Designer	Primer Designer
Polymorphism Detector Specification	Analysis Settings > Polymorphism Detection	Polymorphism Finder
Sequence Trimming Specification	Analysis Settings > Sequence Trimming	Gap MapperLow Confidence Regions Finder

Upload reference plate information

User Goal: Upload plate mapping information (location of target in a 96-well microtiter plate format, A01, A02 etc.) and target sequences into ACE.

For non-HIP users:

- Select cloning strategy to be used (see 'Cloning Project settings' if new cloning strategy needs to be created).
- Prepare XML files for submission.
- Upload XML files:
    - upload reference sequence information using *'Process > Upload plates information > Upload reference sequence information from file';*
    - upload plate mapping information using*'Process > Upload plates information> Upload clone collection from file'* .

For HIP users only: The HIP version of ACE interacts with HIP's cloning database, FLEX. The following section is for HIP users only and is not available to other sites.

- Confirm with informatics team that plates have been submitted for sequencing in FLEX.
- Select *'Process > Upload plates information'*.
    - Submit plate labels (case sensitive) (*Note:* only plates cloned using the same cloning strategy can be uploaded together).
    - Choose from next step in plate processing (available selection: 'Run end reads' and 'Run clone evaluation').
    - Choose 'Start' Codon (available selection: ATG; Natural - as

submitted from GenBank for reference sequence. If your clones have been normalized with start codon other than 'ATG' please contact informatics team).
   - Choose Fusion 'Stop' Codon (available selection: GGA; TTG. If your clones have been normalized with different fusion codon please contact informatics team).
   - Choose Closed 'Stop' Codon (available selection: TGA; TAA; TAG; Natural).
   - Choose cloning vector. If your vector is not available go to *'Cloning project settings > Add vector'* before proceesing.
   - Choose 5' upstream and 3' downstream linkers.
   - Choose project.

Upon finishing the upload ACE will send an e-mail report to the user.

Back

Operational Steps

Once all of the above parameters have been entered in ACE, the application is ready to recieve sequence data and begin to analyze the clones.

Trace file upload

Uploading the trace files to ACE is a critical operation that links the unique plate name created by the sequencing facility to the physical plate of clones and reference information stored in ACE.

- Copy trace files into directory specified in ACE configuration file (TRACE_FILES_TRANSFER_INPUT_DIR).
- Create mapping file, a tab delimited text file with one record per plate. Each record should contain two fields:

<sequencing facility plate label>Tab< HIP plate label>
- In ACE go to *'Trace Files > Create Renaming File'*. Select mapping file, trace files' naming format and type of reads. The 'Internal reads' option currently implemented only supports primers designed by ACE. Click 'Submit' button. ACE writes 'renaming_xxx.txt' file into the TRACE_FILES_TRANSFER_INPUT_DIR directory, the same file is sent to user by e-mail. Renaming file contains one entry per trace file in the format: *<Trace File name> Tab* <ACE recognizable file name>. Make sure

that the number of entries in the renaming file is equal to the number of trace files to be submitted. If the renaming file is empty or the number of entries is less than expected one of the following problems occurred:

- Error in mapping file
- The plate indicated in mapping file was not submitted to ACE .
- There is no trace file name in the selected format.
- Some of the files have not been named properly, for example, some trace files for plate have wrong position annotation.

- Upload trace files on ACE server: *use 'Trace Files > Upload Trace Files'*, select renaming_xxx.txt file created by ACE on the previous step. ACE will send user an e-mail when transfer of the trace files is completed.

Sequencing data processing and clone evaluation

- End Read Processing
- Sequence Assembly
- Discrepancy Finder
- Polymorphism Finder
- Isolate Ranker
- Gap Mapper
- Low Confidence Region Finder
- Finished Clones

End Read Processing

End reads are treated slightly differently from internal reads because some users employ them to select the best candidate from multiple isolates for a single target. End reads processing consists of two steps: (1) placing end read order; (2) uploading of the end reads in ACE.

- To **order end reads** select '*Process > Read Manipulation > Request End Reads Sequencing*', choose vector from drop-down list and click 'Submit' button, select universal primers for forward and reverse reads and select plates to sequence. Be sure to select proper primers, because sequencing primer orientation for vector (see 'Cloning project settings' section for details) determines the way ACE chooses the strand of the sequence while building alignment to

target sequence in *needle*. E.g. if you mistakenly specified M13R as a forward primer while in fact you used M13F for sequencing, ACE will use complement of the end read sequence to build the alignment which will show no similarity to the target sequence. If you run only forward or only reverse reads select 'NONE' option from drop-down list when choosing corresponding primer.

- After traces have been transfered to ACE server (see 'Trace files upload') user needs to **submit end reads** using '*Process > Read Manipulation > Check Quality and Distribute End Reads*' and specify plate name(s). ACE distributes all 'inactive' and internal reads trace files into appropriate directories ('inactive' traces correspond to empty wells and controls). End read trace files for the requested plate(s) that pass the quality check get distributed into corresponding clone directories and their data get submitted into ACE database. ACE sends the user an e-mail with attached report listing all traces that did not pass quality check. This report can be used to upload low quality end reads as 'internal' reads for analysis purposes by using '*Process > Read Manipulation > Submit low quality end reads*' (internal reads are not checked for quality in ACE ; rather, they are stored as trace files in the clone directories).

Sequence Assembly

There are two ways to **run assembler** in ACE:

- '*Process > Read Manipulation > Run Assembler for End Reads*'
- '*Process > Read Manipulation > Assemble Clone Sequences*'

The first option is used after end reads have been submitted only. It applies additional pocessing steps when only one end read of acceptable quality and target sequence is short (< 800 bp). In such cases, ACE extracts the end read sequence from the database, aligns it with the target sequence, verifies that complete coverage is achieved (at least 20 bases upstream / downstream of target sequence are covered by end read sequence) and submits the end read sequence as 'assembled' sequence into the database if coverage is given.

In both cases the user is asked to:

- select library for vector trimming (see 'ACE overview, Assembly Wrapper' for description of vector trimming and ACE configuration file for vector libraries setup);

- determine how low quality reads should be treated;
- set parameters for the quality trimming of independent sequencing reads during the assembly.

Discrepancy Finder

To run Discrepancy Finder (see ACE overview for details) use '*Process > Evaluate Clones > Run Discrepancy Finder*' and specify items that should be analyzed: (a) plate label(s) (case insensitive) - all clones from submitted plate(s) will be analyzed; (b) clone IDs; (c) clone sequence IDs (user can get clone sequence ID by running 'General Report'), and set quality cut-off based on Phred score (default set to 25).

Polymorphism Finder

To run Polymorphism Finder (see ACE overview for details) use '*Process > Evaluate Clones > Run Polymorphism Finder*', specify items that should be analyzed and choose Polymorphism Finder Specification. ACE creates three intermediate files and puts them into a directory specified by POLYMORPHISM_FINDER_DATA_DIRECTORY variable in ACE configuration file. These files will be automatically transferred to the dedicated server that hosts GenBank database(s), and the results of the Polymorphism Finder run performed on the server will be transferred back and uploaded into ACE. Please contact ACE administrator for the details when and how the data file transfer is arranged. You can view Polymorphism Finder results by using comprehensive plate viewer ('*View > Plate Results*'), click on well link for your clone, click 'Discrepancy Report' button on sample view for clone sequence - the value in 'Polymorphism' column for each discrepancy will be changed to 'Y' (discrepancy is known polymorphism) or 'N' (no hit for discrepancy was found). 'Details' button gives you access to the list of GenBank GI record(s) that were found as hits for the particular discrepancy.

Isolate Ranker

To run Isolate Ranker (see ACE overview for details) select '*Process > Evaluate Clones > Run Isolate Ranker*' and specify plate label(s) (case insensitive) and process specifications (see 'Analysis settings' section for details). Isolate Ranker can operate on isolates for the same target located on different plates; however, these plates must be run by module at the

same time. Isolate Ranker will sort isolates based on the most recent contig for the particular clone, e.g., if clone has end reads, the collection of contigs and gaps defined by Gap Mapper and several clone sequences assembled under different conditions (see Assembler description for details) isolate will be ranked based on LAST assembled sequence. The result is shown in 'Plate viewer and sample reports'.

Gap Mapper

User may run 'Gap Mapper' to determine gaps in clone coverage (see ACE overview for details).

To operate 'Gap Mapper' use '*Process* > *Evaluate Clones* > *Run Gap Mapper*', enter clone IDs and specify sequence assembly parameters. These parameters should be set because Gap Mapper invokes sequence assembler as first step of processing (consult ACE overview). User can instruct ACE to calculate low confidence regions (LCR) of assembled contigs by selecting '*Run LQR Finder on contig sequences*' option. Gap Mapper can be run in "try mode"; in this case the information about gaps and LCRs is only emailed to the user as a tab delimited text file, but not stored in database and cannot be used later for region specific primer(s) design.

Low Confidence Region Finder

If an assembled clone sequence has low confidence discrepancies (Discrepancy Finder) that should be resolved before clone acceptance, the user should run 'Low Confidence Region Finder' to determine the presence of a low confidence region(s) (LCR), which allows for region specific design of internal primer(s) in 'Primer Designer' (see ACE overview for details).

To access this functionality use '*Process* > *Evaluate Clones* > *Run Low Confidence Regions Finder in Clone Sequences*', specify clone IDs and select specification (see 'Analysis settings' section for details). If you are going to repeat end reads using universal primers, set the number of bases that are expected to be covered by these end reads. 'Low Confidence Regions Finder' will not define LCR for these regions. The Finder can be run in "try mode"; in this case the information about LCRs is only emailed to the user as a tab delimited text file, but not stored in database and cannot be used later for region specific primer(s) design.

Finished clones

Once user decides that a clone has met acceptance or rejection criteria (based on Decision Tool report or manual inspection), it is possible to assign a final status for the clone in ACE. This prohibits further processing of clone data, thus reducing the job size and focusing efforts on clones still pending. A clone with status other than 'In Process' (default clone status assigned on clone submission into ACE) is excluded from the following processes: (a) assembly; (b) primer design and order; (c) polymorphism search; (d) data deletion (see Data clean-up section for details). To set **clone final status** select '*Process > Set Final Clones Status*' and specify clone IDs and final status. Optionally, an XML file with results of clone analysis can be created. User can change clone status back to 'In Process' at any time, which allows full processing again.

Reports

In the report section, 6 types of reports can be requested from ACE, all will be send to user by e-mail as attached, tab delimited file(s), and where noted below also created on-line in ACE.

- '**Quick Decision Tool**' report is a simplified version of the Decision Tool which determines if clone(s) meets user defined acceptance criteria. To run the report select *Reports > Quick Decision Tool'* and specify user acceptance criteria and set of clones. Execution time is proportional to the number of specified clones. The 'Quick Decision Tool' provides feedback online in addition to sending a report by e-mail.
- To run **Detailed Decision Tool** choose *'Reports > Detailed Decision Tool'* and fill the query form. There is no limit on the number of clones that can be processed.
- '**Mismatched Clones**' report helps to detect cases of misidentified input data such as (a) mislabeled or rotated plates; (b) cross-contamination on the clone plate; (c) systematic errors. End read sequences are compared against user selected database using NCBI BLAST. Database should contain reference sequences for the project and vector sequences. To run the report select *' Reports > Mismatched Clones'*, specify clones and database. There is no limit on the number of clones that can be processed at any given time.

- **'General report'** provides a snapshot of requested information for the specified set of query objects, which can be either plate(s), clone IDs, user reference sequence IDs, assembled clone sequence IDs (clone sequence IDs can be extracted from General Report or Detailed Decision Tool report files). To run general report choose *'Reports > General Report'* and fill in the query form. There is no limit on the number of query objects that can be processed.
- The **'Trace File Quality'** report allows users to examine quality of existing trace files, and can be accessed at *'Reports > Trace Files Quality Report'*. Either plate labels or clone IDs can be provided, and for each clone trace file ACE extracts Phred confidence base scores and determines whether a read meets minimum quality requirements: (a) the read must be longer than the user-defined minimum length; (b) the average confidence score for all non-ambiguous bases between the first and last base must be above the minimum confidence score. The value of these thresholds are defined in ACE configuration file. There is no limit on the number of clones that can be processed.
- To obtain a list of end reads that failed and should repeated the user can invoke the **End Read Report** '*Reports > End Reads Repor*t' for any plate(s).

Back

Primer design and management

This set of features allow users to: (a) design gene-specific sequencing primers; (b) select specific primer(s) for synthesis; (c) order primers; (d) track primer plates;

- To **order gene-specific primers** use '*Process > Internal Primer Design and Order > Run Primer Designer*', specify how to extract clone information. User can submit (a) plate labels - primers will be designed for all clones from these plates; (b) clone IDs; (c) ACE reference sequence IDs; (d) user reference sequence IDs*. Next choose specification for Primer3 and define what region of the target sequence the primer should cover. Select '*Design primers for Reference Sequence*' option if you would like to design primers for a primer walk independent of (end read) coverage. Select '*Design primers for Stretch Collection*' to design primers needed to complete coverage (fill in gaps or cover low confidence discrepancy region). Stretch collection is stored in ACE after Gap Mapper or Low

Confidence Regions Finder was run for a particular clone. ACE extracts only the most recent stretch collection defined for a clone to be used in primer design based on stretch collections.

Primer Designer can be run in try mode, in this case information about designed primers is only sent to user in tab delimited text file, however, no information is stored in database.
****Note:*** Options (c) and (d) should not be used in conjunction with '*Design primers for Stretch Collection*' mode.

- Designed primers must be approved by user to be included in vendor order. To **approve primers** select '*Process > Internal Primer Design and Order > Approve Internal Primers*', specify clone IDs and select the kind of primers (designed for reference sequence or for stretch collection) that
should be displayed for approval. User can approve primers designed under both options for any clone for primer synthesis.
- To **create a primer order** use *'Process > Internal Primer Design and Order > Order Internal Primers'*, specify clone IDs and choose type of approved primers that should be included in the order. Available options are (a) only primers designed for stretch collections; (b) only primers designed for reference sequence; (c) primers designed for stretch collection(s) together with primers for reference sequence. ACE selects all primers approved for the specified clones according to the user selection and maps them in A01-H12 96-well plate format. User has the option to specify the first and last primer position on a plate, preserving empty wells for controls if desired. ACE supports multipal output formats to create an order file compatible with vendor order sheets (sample_order_file1, sample_order_file_2).
- To store **information** regarding **primer plate** orders or usage select '*Process > Internal Primer Design and Order > Track Oligo Plate*', choose plate status and enter comments (optional). This step is requered if user intends to use primers more than once (i.e., second clone for target sequence)

Real time views

Two different view options are available. Under *'View'* more general, plate, clone, or project oriented data are provided and the outcome of specific processes visually accessible under '*Process > View Process Results*'.

- '**Plate viewer and sample reports**' view gives access to complete

information about a plate. Select '*View > Plate Results*', enter plate label (case insensitive), check 'Show Isolate Ranker Output' option.

- To view **plate history** select '*View > Plate history*' and enter plate label (case insensitive);
- To view **plate description** select '*View > Plate Description*' and enter plate label (case insensitive). The view displays (1) plate label; (2) plate unique ID; (3) plate type (96 well plate); (4) link to cloning strategy description; (5) for each well: (a) well number; (b) sample type - Isolate, Control, Empty; (c) clone ID; (d) clone final status; (e) description of the last process run; (f) link to reference sequence description; (g) link to clone sequence description if available.
- To view **clone processing history** select '*View > Clone History*' and enter clone IDs. The interface gives the user information about all steps a clone has passed in ACE, links to step specific specification, date and time of execution for each step, and user performed task.
- To view **all plates uploaded into ACE** select '*View > Plates*'. Plates are grouped by project name (alpha numerical order). Click on the check box located before project name to hide all plates related to the project.
- To view **any analysis specifications** stored in ACE select 'View > Process Configurations'. ***Note:*** user can view only one specification at a time.
- To view complete information about **vectors** uploaded into ACE select '*View > Vectors*'.
- To view complete information about **linkers** uploaded into ACE select '*View > Linker*'.
- To examine **quality of the uploaded end reads** select '*Process > View Process Results > View available end reads*'. The resulting view displays (1) plate label; (2) plate unique ID; (3) plate type (96 well plate); (4) link to cloning strategy information; (5) for each well: (a) well number; (b) sample type - Isolate, Control, Empty; (c) quality status of forward end read (Pass / Fail); (d) quality status of reverse end read. The view helps to detect systematic problems with submitted end reads, for example, cases when all forward or reverse reads failed.
- To view **primer designed for clone(s)** select '*Process > View Process Results > View Internal Primers* ', submit clone IDs and indicate type of primers: primers designed for reference sequence or primers designed for stretch collection (see description of GapMapper module for details). Sample report.
- To view **all primer ordered for a clone** select '*Process > View Process Results > View Oligo Order(s) for Clone(s)*'. Sample report.
- To view **ordered oligo plate(s)** select '*Process > View Process*

Results > View Oligo Plate'. Sample report.
- To view **all contig collections** created by Gap Mapper for a clone select '*Process > View Process Results > View all contig collections* '. Multiple contig collections are created when Gap Mapper is launched several times with a different specification or with additional trace files.
- To view **only the last contig collection** designed for the clone by Gap Mapper select *Process > View Process Results > View latest contig collection*. Sample report.
- To view **low confidence region(s)** for a clone created by Low Confidence Finder select *Process > View Process Results > View low confidence regions for clone sequences'* and specify clone IDs. Sample report.

Back

Data clean up

ACE allows removal of trace files and clone information from the database. This is useful in cases when: (a) wrong plates have been submitted to ACE; (b) errors occurred in clone mapping on the plate; (c) plates have been mislabeled; (d) incorrect data from sequencing facilities, etc.

- To **delete plate** select '*Process > Delete Data > Delete plate*' and submit plate label (case insensitive).
- To **delete clone forward and reverse end reads** select '*Process > Delete data > Clone forward and reverse end reads from database*' and submit plate label (case insensitive) or clone IDs. This action deletes records from the database; however, trace files for these end reads stored on the hard drive are not affected.
- To **delete forward end reads** only select '*Process > Delete data > Delete clone forward end reads from database*' and submit plate label (case insensitive) or clone IDs. This action deletes records for the forward reads from the database; however, trace files for these forward end reads stored on the hard drive are not affected.
- To **delete clone reverse end reads** select *'Process > Delete data > Delete clone reverse end reads from database*' and submit plate label (case insensitive) or clone IDs. This action deletes records for the reverse reads from the database; however, trace files for these reverse end reads stored on the hard drive are not affected.
- To **delete clone sequence** select '*Process > Delete data > Delete clone sequence from database*' and submit clone ID or clone

sequence ID (run General Report to get clone sequence ID).

- To **delete trace files** from the hard drive, first, select '*Process > Delete data > Get trace file names*'. ACE will send e-mail with attached file that contains trace file names for the submitted clone IDs. Save this file. Next select '*Process > Delete data > Delete trace files from hard drive (no recovery possible)*' and submit saved file. ACE will permanently delete traces from the hard drive.
- To **remove trace files** to storage directory first get file with trace file name from ACE (see above). Next select *'Process > Delete data > Move trace file from clone directory into temporary directory (allows trace files recovery)*' - traces will be moved to the storage directory.
- To **delete intermediate files** created during clone sequence assembly by Phred/Phrap from hard drive select '*Process > Delete data > Clean-up hard drive*'.

Back

© 2005 by Helen Taycher last changed March 17, 2007

```
<?xml version="1.0" encoding="ISO-8859-1" ?>
<!DOCTYPE web-app (View Source for full doctype...)>
- <vector-info>
    <!--   Vector data     -->
    <!--   vector type 0  master , 1  expression     -->
  - <vector>
      <vector-name>pDNR-Dual</vector-name>
      <vector-source>BD Biosciences (Clontech)</vector-source>
      <vector-type>0</vector-type>
    - <vector-feature>
        <feature-name>recombination site</feature-name>
        <feature-description>LoxP</feature-description>
        <!--   feature type -> 1 - added; 0 - remain; -1 - lost            --
        >
        <feature-type>0</feature-type>
      </vector-feature>
    - <vector-feature>
        <feature-name>antibiotic selection marker</feature-name>
        <feature-description>CmR</feature-description>
        <feature-type>0</feature-type>
      </vector-feature>
    - <vector-feature>
        <feature-name>antibiotic selection marker</feature-name>
        <feature-description>SacB (reverse orientation)</feature-description>
        <feature-type>0</feature-type>
      </vector-feature>
    - <vector-feature>
        <feature-name>origin of replication</feature-name>
        <feature-description>pUC ori</feature-description>
        <feature-type>0</feature-type>
      </vector-feature>
    - <vector-feature>
        <feature-name>multiple cloning site</feature-name>
        <feature-description>MCS</feature-description>
        <feature-type>0</feature-type>
      </vector-feature>
    - <vector-feature>
        <feature-name>splice donor site</feature-name>
        <feature-description>CD</feature-description>
        <feature-type>0</feature-type>
      </vector-feature>
    - <vector-feature>
        <feature-name>negative selection marker</feature-name>
        <feature-description>SacB</feature-description>
        <feature-type>0</feature-type>
      </vector-feature>
    </vector>
  </vector-info>
```

Screenshot of form to link vector with sequencing primer

Home > Cloning Project Settings > Link Vector with Sequencing Primer

Link Vector with Sequencing Primer

Please select vector:	pDONR201
Please select sequencing primer:	pDiddy
Please select sequencing primer position:	5 prime
Please select sequencing primer orientation	Sense

Submit

Currently Available Vector - Sequencing Primer Pairs

Vector Name	Primer Name	Primer Position	Primer Orientation
pBY011	PBY011 N-term primer	5 prime	sense
pBY011	PBY011 C-term primer	3 prime	antisense
pDNR-Dual	M13F	5 prime	sense
pDNR-Dual	T7	5 prime	sense
pDNR-Dual	pDiddy	3 prime	antisense
pDONR201	Reverse	3 prime	antisense
pDONR201	Waugh	5 prime	sense
pDONR201	SEQL_B	3 prime	antisense
pDONR201	Gateway_FarF1	5 prime	sense
pDONR201	pDONRATG	5 prime	sense
pDONR201	pDONRRSTOP	3 prime	antisense
pDONR221	M13R	3 prime	antisense
pDONR221	M13F	5 prime	sense

ACE Configuration

ACE is configured by setting values in ApplicationHostSettings.properties. The presence of a configuration file is verified by ACE on Tomcat Web Server start-up. The settings are verified by ACE on application start-up.

ACE_CC_EMAIL_ADDRESS	ACE administrator e-mail address (optional)
ACE_FROM_EMAIL_ADDRESS	ACE application e-mail address
JSP_REDIRECTION	/ACE/ - location of ACE application
mail.smtp.host	Location of SMTP host that is used for sending e-mails
CODON_START_1 ... *N*	Values of 'start' codon
CODON_START_1...*N*_NAME	User friendly of 'start' codon
CODON_STOP_CLOSED_1...*N*	Values of 'stop' closed format codon
CODON_STOP_CLOSED_1...*N*_NAME	User friendly of 'stop' closed format codon
CODON_STOP_OPEN_1...*N*	Values of 'stop' fusion format codon
CODON_STOP_OPEN_1...*N*_NAME	User friendly of 'stop' fusion format codon
BLAST_DBASES_PATH	Storage for blastable databases created by user. These databases are used by 'Mismatched Clones' report (see user guide)
BLAST_OUTPUT	Path to directory where BLAST output file will be stored, can be set to /tmp/
BLASTABLE_DB_1...*N*	Location of user blastable database
BLASTABLE_DB_1...*N*_NAME	Name of user blastable database
IS_DEBUGING	0 (false) for all none-developer users
IS_HIP_VERSION	0 (false) for all non-HIP installations
PLATE_TYPE_WELL_NAMING	1 . Naming format for the 96-plates A01-H12. No other format is supported by current version

PHRED_QUALITYDEF_FIRST_BASE<br>PHRED_QUALITYDEF_LAST_BASE<br>PHRED_QUALITYDEF_MIN_LENGTH<br>PHRED_QUALITYDEF_SCORE_PASS	Parameters used by 'Trace Files Quality Report' to define quality of reads
PRIMER3_EXE_PATH	Path to Primer3 executable
PHRED_EXE_PATH	Path to Phred executable. For Windows users: Phred can be compiled under WinOS
NEEDLE_EXE_PATH	Path to needle executable. For Windows users: needle must be compiled under cygwin
PERL_PATH	Path to UNIX version of perl
PHREDPHAP_SCRIPT_PATH	Path to phredPhrap.perl script
BLAST_EXE_COMMON_PATH	Path to NCBI BLAST executable
TEMPORARY_FILES_FILE_PATH	Stores all temporary files created by ACE; the directory should be emptied periodically (see Clean up cron jobs)
TRACE_FILES_INPUT_PATH_DIR	The initial trace file location. This directory can reside on any computer, however, server running ACE should be able to access the directory
TRACE_FILES_OUTPUT_PATH_ROOT	Root directory for trace files arranged by clone; resides on ACE server
TRACE_FILES_TRANCFER_INPUT_DIR	The initial trace file location for traces renamed according to ACE format. Traces moved into this directory from TRACE_FILES_INPUT_PATH_DIR. This directory should reside on ACE server
TRACE_FILES_TRANSFER_OUTPUT_DIR	Should be equal toTRACE_FILES_TRANCFER_INPUT_DIR for any typical installation
MOVE_TRACE_FILES_BASE_DIR	Path to directory where trace files will be moved, when user runs 'Move trace file from clone directory'
NEEDLE_OUTPUT_PATH	Directory for *needle* alignments. All '*.in' files from this directory should be deleted by clean-up cron job

NEEDLE_OUTPUT_TMP_PATH	Output directory for temporary needle alignments; the directory should be cleaned up periodically. Optionally, these files can be directed into temp directory
VECTOR_FN_LIBRARY_DEFAULT	File name for vector library (FASTA format) that contains sequences for all vectors used by group
VECTOR_FN_LIBRARY_DEFAULT_NAME	User friendly name for 'all vectors' library
VECTOR_FN_LIBRARY_EMPTY	Empty text file, used as vector library file when 'No trimming' option is specified
VECTOR_FN_LIBRARY_EMPTY_NAME	'No trimming' - user option that indicates that no vector trimming will be performed by cross_match
VECTOR_FN_1...*N*	File name for vector library (FASTA format)
VECTOR_FN_1...*N*_NAME	User friendly vector name
POLYMORPHISM_FINDER_DATA_DIRECTORY	Input/output files for Polymorphism Finder will be stored here. This directory is needed if Polymorphism Finder will be used, set value to /tmp otherwise
PF_DB_1...*N*	Name of blastable database name for Polymorphism Finder
PF_DB_1...*N*_NAME	User friendly blastable database for Polymorphism Finder
PF_PATH_DB	Location of blastable database used by Polymorphism Finder on server
FILE_NAME_INPUT_DATA_FILE	File name for discrepancy data file created by Polymorphism Finder
FILE_NAME_ORFSEQUENCES_DATA_FILE	File name for ORF sequence text file created by Polymorphism Finder
FILE_NAME_ORFSEQUENCES_INDEX_FILE	File name for ORF sequences index file created by Polymorphism Finder

© 2005 by Helen Taycher last changed January 15, 2007

Clone Ranking Specification

For cloning workflows that produce several isolates for the same gene with the expectation that at least one of the isolates will be of acceptable quality, it is essential to select the best isolate to carry forward as soon as some sequencing data is available. Isolate Ranker ranks isolates based on overall isolate score which is computed by normalizing the sum of the penalties over the number of bases covered.

Main form to create new Clone Ranking Specification accessible through '**Analysis Settings > Clone Ranking**'

Create New Set of Parameter for Clone Ranking

View Mine   View All

Set Name [   ] - unique specification name

Penalties for discrepancy in the insert region

(•) Process conservative & non-conservative substitutions together (System will use the penalty values for missense substitutions; system will ignore values for conservative and non-conservative substitutions)

( ) Process conservative & non-conservative substitutions separately (System will use the penalty values for conservative & non-conservative substitutions; system will ignore values for missense substitutions)

Base Confidence	High	Low
Silent substitution	3	1
Missense substitution	20	10
Conservative substitution	20	10
Non-conservative substitution	50	30
Frameshift	1000	1000

**In-frame deletion**	1000	1000
**In-frame insertion**	1000	1000
**Truncation**	1000	1000
**No translation (e.g., no ATG)**	1000	1000
**Post-elongation (e.g., no stop codon)**	1000	1000

Penalties for discrepancies in the linker region

Base Confidence	High	Low
5' substitution	3	1
5' deletion/insertion	1000	500
3' substitution	50	30
3' deletion/insertion	1000	500

Penalties for discrepancies introduced by ambiguous bases

☑ Show

Base Confidence	High	Low
**Start codon substitution**	0	0
**Stop codon substitution**	0	0
**Substituttion cds region**	0	0
**Frameshift insertion**	0	0
**Inframe insertion**	0	0
**Substitution in 5' linker region**	0	0
**Insertion in 5' linker region**	0	0
**Substitution in 3' linker region**	0	0
**Insertion in 3' linker region**	0	0

Clone Acceptance Criteria Specification

Clone Acceptance Criteria Specification establishes criteria for clone acceptance by setting the maximum allowed number of discrepancies of each type. Different values can be set for discrepancies of low and high confidence (see for discrepancy confidence definition). The user sets values for two thresholds: 'PASS' - clone that has less or equal number of discrepancies will be automatically accepted, 'FAIL' threshold automatically rejects the clone. Clones that fall between these two thresholds should be manually evaluated by user. Set both thresholds to the same value to fully automate process of clone evaluation. Users can also opt to handle conservative and non-conservative amino acids substitutions differently or to treat them as one type. Selecting the '**Ignore if polymorphism**' check box for the discrepancy type excludes discrepancies of this type from total count if they represent known polymorphism as defined by Polymorphism Finder.

Main form to create new Clone Acceptance Criteria Specification accessible through '**Analysis Settings > Clone Acceptance Criteria**'

Create New Set of Parameter for Clone Acceptance Criteria

View Mine **View All**

Set Name [ ] - unique specification name

Maximum acceptable number of discrepancies (insert region)

◉ Process conservative & non-conservative substitutions together (System will use the penalty values for missense substitutions; system will ignore values for conservative and non-conservative substitutions)

○ Process conservative & non-conservative substitutions separately (System will use the penalty values for conservative and non-conservative substitutions; system will ignore values for missense substitutions)

Threshold	PASS	FAIL	Ignore if polymorphism

Base Confidence	High	Low	High	Low	
Silent mutation	9	9	15	15	☐
Missense substitution	1	0	3	10	☐
Conservative substitution	1	0	3	10	☐
Nonconservative substitution	1	0	3	10	☐
Frameshift	0	0	1	9	☐
Inframe deletion	0	0	3	9	☐
Inframe insertion	0	0	3	9	☐
Truncation	0	0	1	9	☐
No translation (e.g., no ATG)	0	0	2	2	☐
Post-elongation (e.g., no stop codon)	0	0	2	2	☐

Maximum acceptable number of discrepancies (linker region):

Threshold	PASS		FAIL	
Base Confidence	High	Low	High	Low
5' substitution	0	0	4	9
5' deletion/insertion	0	0	1	6
3' substitution	0	0	4	9
3' deletion/insertion	0	0	1	6

Maximum acceptable number of discrepancies introduced by ambiguous bases:

☑ Show

Threshold	PASS		FAIL	
Base Confidence	High	Low	High	Low
Start codon substitution	0	0	19	99
Stop codon substitution	0	0	19	99
Substitution cds region	0	0	19	99
Frameshift insertion	0	0	19	99
Inframe insertion	0	0	19	99
Substitution 5' linker region	0	0	19	99
Insertion 5' linker region	0	0	19	99
Substitution 3' linker region	0	0	19	99
Insertion 3' linker region	0	0	19	99

ACE Overview

ACE Overview

Automated Clone Evaluation (ACE) is a comprehensive, multi-platform and multi-user, web-based sequence verification software suite especially suited for protein expression clones. It automates all steps of the sequence verification process: sequence contig assembly, identification and annotation of differences between the clone and a reference sequence, gap mapping, primer design, polymorphism detection, and assignment of final clone quality based on user defined criteria. ACE utilizes well-established algorithms and third-party programs (Phred/Phrap, BLAST, *needle*, Primer3) to perform specific tasks. It provides an integrated environment that relieves the user from routine process management tasks, such as bookkeeping of all project- and clone-related information, re-entering of parameters and criteria, and history tracking.

ACE is structured for maximum flexibility to support different approaches to clone validation and sequencing management. Users do not need to follow a single path in clone sequence verification, but rather can invoke each module individually. A typical workflow used in our laboratory is shown in Sequence verification workflow. A project usually begins with end read sequencing of one or more clonal isolates per target sequence. End reads are acquired, assigned to their corresponding clone (***End Read Processor***), and then processed by the assembler to determine if end reads alone are sufficient to yield a complete assembly (***Assembly Wrapper***). Whether or not the assembly yielded a single contig covering the full-length CDS the clone contig(s) is analyzed to detect differences or “discrepancies” compared with the reference/target sequence (***Discrepancy Finder***). ACE can compare any discrepancy with one or more sequence database such as GenBank, to determine if they correspond to a naturally occurring polymorphism (***Polymorphism Finder***). During the final decision process, users can optionally configure the software to avoid penalizing clones for discrepancies that represent polymorphisms (*see* ***Decision Tool***).
Clones that fail to assemble into a single contig covering the CDS can be processed to find the remaining gaps in sequence coverage (***Gap Mapper***). In addition, clones with discrepancies based on low confidence sequence can be processed to delineate these low confidence regions (***Low Confidence Regions Finder***). Subsequently, clones with low confidence regions or gaps in sequence coverage can be processed to define appropriate internal sequencing primers to generate high confidence clone sequence (***Primer Designer***).

If more than one isolate exists for a given clone (target sequence), an optional module (***Isolate Ranker***) can rank isolates based on user-defined preferences specified in the form of penalties associated with different types of discrepancies.

At any stage during the clone verification process, a set of clones can be processed by the Decision Tool in order to determine how far each clone has progressed in the analysis pipeline and its acceptance/rejection status. This feature allows all accepted and rejected clones to be harversted or eliminated as soon as possible removing completed clones from the pipeline.

Core Module Description

- Trace File Distributor
- End Read Processor
- Assembly Wrapper
- Internal Primer Management
- Discrepancy Finder
- Polymorphism Finder
- Isolate Ranker
- Decision Tool
- Gap Mapper
- Low Confidence Region Finder

1. Clone Sequencing Support

Trace File Distributor Clone validation projects often include sequence reads from similar genes (e.g., paralogs, multiple isolates of the same gene, etc.). It is essential that reads from closely related clones do not end up in one another's analysis. ACE creates a hierarchical directory structure and stores all files related to a single clone in a directory specific to that clone. Within this directory ACE creates a number of subdirectories that hold trace files, consensus sequences, contigs, etc. as required by the Phred/Phrap package. Distribution of trace files is accomplished by parsing information encoded in the filename for each trace file that indicates which gene and clone it belongs to. Sequencing facilities often encode their own read identification information (such as plate and well identifiers, read direction, number of attempts and/or the primer name) into trace file names and this information can be used to link a read to its original sample. However, because different facilities utilize vastly different naming conventions, a mechanism must exist to rename files into a format compatible with the ACE parser. Users employ the Trace File Distributor to describe the specific naming format for trace files in a way that allows ACE to process these files automatically. Multiple sets of rules to extract plate, well name, and read direction can be created by the user to accomodate multiple naming conventions (Translation of trace file name from sequencing facility name to ACE format). Detailled descriptions of how to create a new Trace File Format can be found here.

End Read Processor End reads are treated differently from internal reads because some users employ them to select the best candidate from multiple isolates of the same target. We have found that poor quality end reads will lead to the rejection of about 25-35% of good clones based on discrepancies that turn out to be sequencing errors. To avoid this problem, the End Read Processor invokes the Phred base caller to determine whether a read meets minimum quality requirements. A read must be longer than the system administrator-defined minimum length (default = 250 nt) and the average confidence score for all non-ambiguous bases between the first and last base (default: first base = 50, last base =700) must be above the user-defined minimum confidence score (default = 25). End reads that fail to meet these criteria are disregarded in the computation for ranking isolates, but the files remain in the system and the user can add them later as internal reads for inclusion in the clone sequence assembly. Alternatively, a user might choose to submit all available reads, including end-reads, as internal and build contig collections (see ***Gap Mapper***).

Assembly Wrapper This tool automates contig assembly by calling the Phred/Phrap assembler for each clone on a user-submitted list. Some contigs that fail assembly with default parameters can be assembled by adjusting a variety of trimming parameters.
Vector trimming during contig assembly is performed using 'Cross_match'. In some cases, trimming is essential for contig assembly, whereas in others, it prevents assembly. For example, a high degree of similarity between target sequence and sequences in the vector library causes Phred/Phrap to mask valid (i.e. gene) sequence when vector trimming is applied blindly. This problem can be partially alleviated by removing irrelevant vector sequences from the vector library up front. Moreover, editing vector sequences down to about 300 bp of insert-flanking regions might further improve success. The modified trimming strategy works in most cases but occasionally it might be necessary to turn off vector trimming altogether to get assembly for particular clones.
In addition to vector trimming, ACE enables quality trimming of independent sequencing reads during the assembly. When prompted, ACE trims all reads prior to assembly by removing bases prior to base 50 and after base 900 (user-adjustable values). Furthermore, reads with a low average confidence score or below a minimum length can be completely excluded from assembly.

Internal Primer Management This suite of modules allows users to: (a) design gene-specific sequencing primers; (b) select specific primer(s) for placement on a vendor order; (c) view all primers (both designed and ordered) and their design specifications, and (d) track and manage primer plates and individual primers.
The Primer Designer can be used *a priori* to design gene-specific primers for a complete primer walk or to design only those primers needed to complete coverage (i.e., fill in gaps) or to resequence regions of low base confidence (see Gap Mapper and Low Confidence Regions Finder). The user must create prior to Primer Designer invocation specification to indicate: (1) type of desired coverage (single forward, single reverse, double coverage, etc.); (2) primer sequence related parameters (Tm, window size, GC content); (3) sequence

processing parameters (length of reliable part of sequencing reads). The module breaks the sequence into fragments which are provided to *Primer3* for primer prediction. Fragment size takes into account the expected distance between the primer and reliable sequence, expected high quality read length, and the *Primer3* window size, which are user-specified values. The Primer Design module then collates the output of *Primer3*.

2. Contig Analysis

Discrepancy Finder. This module detects mismatches between the clone contig(s) and its reference sequence and creates a list of discrepancy descriptions for each clone.
The Discrepancy Finder builds a global alignment between the clone contig(s) and its reference nucleotide sequence using the *needle* program from the EMBOSS package. Mismatches between the sequences are identified by base-by-base comparison and a discrepancy object is created for each difference, unless mismatches are contiguous (e.g., a multiple base-pair deletion in one region) in which case they are grouped together to form a single discrepancy object.
A discrepancy in ACE refers to any mismatch between the clone and its target sequence described on both the nucleotide and polypeptide levels. ACE assigns confidence status (low or high) to any discrepancy. Low confidence status is assigned to a discrepancy if a Phred confidence score of at least one base is used to define the discrepancy or if one out of four bases on either side of the discrepancy is below the a user-defined threshold (default set to 25).

Polymorphism Finder This module determines if discrepancies are attributable to natural sequence variations of a gene, which is particularly relevant for human genes. The process requires two steps: (1) a relatively short sequence segment comprising the discrepancy and its flanking nucleotides is compared using NCBI BLAST with data from all user-selected GenBank databases to find an identical match; (2) each 100% hit is verified by using Pairwise BLAST comparing the entire clone target sequence with the hit sequence, to gain confidence that it belongs to the same gene. A match provides good evidence that the observed sequence variation occurs naturally, but the absence of a match is inconclusive. Length of flanking sequences attached to each discrepancy and list of GenBank databases to search for the match are stored as user defined specifications for Polymorphism Finder and can be reused. The Polymorphism Finder stores the NCBI GI number for each verified hit as part of the discrepancy description. ***Note:*** As this operation requires sending numerous BLAST queries against large databases, its implementation requires a local copy of the appropriate GenBank database(s) installed on a dedicated high-performance computer or cluster.

3. Clone evaluation

Isolate Ranker Some cloning workflows produce several isolates for the same gene with the

expectation that at least one of these isolates will be of acceptable quality. Isolate Ranker selects the best isolate to carry forward by comparing isolates based on partial or full sequencing data applying user defined penalties for different discrepancy types. For each combination <discrepancy type, confidence>, the user specifies two values: maximum permitted number of such discrepancies and the penalty per discrepancy. Isolates that exceed the maximum permitted number of mutations of at least one type are rejected. For each remaining isolate, the overall score is computed by normalizing the sum of the penalties over the number of bases covered. These scores determine the rank order among the surviving isolates of the same gene. Special care should be taken when comparing isolates with full coverage of the reference sequence and with isolates having partial coverage - shorter contigs might have smaller absolute number of discrepancies but poorer quality. Using an average number of discrepancies per base is also not adequate, since averages become unreliable as the length of coverage decreases. Instead we have chosen to augment discrepancy counts for each partial coverage contig by pseudo-counts based on the length of the uncovered sequence and the average frequency of discrepancies across all reads. The rank order among the surviving isolates of the same gene are displayed as a color coded virtual plate map allowing user quickly to pick follow-up clones as well as determine if there are any systematic problems with cloning or sequencing (e.g., clones in the same position repeatedly fail, all clones fail to match reference).

Decision Tool This tool sorts clones into a finished-and-accepted group, a rejected group, and a set of groups slated for further processing. The Decision Tool flowchart illustrates the logic path for assigning clones to different action groups. The goal here is to bin clones that require the same next process step (e.g., map gaps, assemble sequence, finished and accepted) and report them in a single file. Fundamentally, the Decision Tool accomplishes this by comparing each clone's discrepancy list to a user-defined list of acceptance criteria, which defines how many discrepancies of each type are permitted. Users can set thresholds to automatically accept clones that meet some high level criteria and automatically reject clones that fail to meet some minimal criteria. Moreover, users can set independent thresholds for high and low confidence discrepancies. Any clones neither accepted nor rejected remain in process. Users may wish to apply alternate acceptance criteria to the same set of clones for different experimental purposes. User specifications for acceptance criteria are stored as named sets in the software and can be invoked and applied to any collection of clones.

4. Finishing tools

Gap Mapper This module finds gaps in sequence coverage that arise when reads are too short or fail outright. The module uses the Phred/Phrap assembler to align clone traces with

the reference sequence, which is included as a “pseudo-read” with a preset confidence score used for every base (arbitrarily set at 19). This ensures complete assembly without forcing the contig to be identical to the target sequence. The assembler output is parsed to determine the alignment of each sequence read relative to the reference sequence. Using this positional information, a two-dimensional matrix is created wherein each position is described by a base and its confidence score (Gap Mapper contig calculation). At each position along the matrix (which is numbered according to the reference sequence), a consensus base is determined for the clone by assessing all of the bases at that position and their confidence scores using a naïve Bayesian calculation. The reference sequence and the assembled contig are disregarded at this step so that the computed confidence scores reflect the actual clone sequence. The resulting contigs are trimmed at both ends to remove bases with low confidence scores (using the sliding window algorithm described in Low Confidence Region Finder). This ensures that the predicted new sequence primers will generate sequence that covers these junction regions. Once the contigs are assembled, trimmed and mapped to the reference sequence, gaps are defined as 'stretches' of reference sequence not covered by contigs. Gap and contig information is stored in the database as contig collection(s) and used to assess the clone status and quality, and can be passed to Primer Designer to design primers for clone finishing using the 'stretch collection' option.

Low Confidence Region Finder We have found that clones demonstrate a high number of discrepancies in regions where sequence confidence is low. Even in cases where the discrepancy itself has a high confidence score it may be surrounded by low confidence segments calling into question the sequence quality of the entire region. The Low Confidence Region Finder was designed to identify such low confidence regions (LCR) in contigs by applying a ‘sliding window’ algorithm. The user specification for the module defines: (a) the width of the window ***'W'***; (b) the cut-off Phred score for low-confidence bases ***'m'*** and the maximum allowable number of low-confidence bases ***'N'***. An LCR is defined as a region in which a window of ***W*** consecutive bases contains at least ***N*** low-confidence bases. LCRs located within **X** bases (user specified parameter, default value X = 50 bp) to each other are considered one region.

Sequence verification workflow

The diagram illustrates a typical workflow for the full length sequence validation of a protein coding clone. The process, which is described in detail in the text, is an iterative process that collects sequence reads, assembles contigs, finds gaps in coverage, finds regions of low confidence, compares the contig sequence with the expected sequence, and determines the overall acceptability of the clone. Processing steps in parentheses and italics are optional.

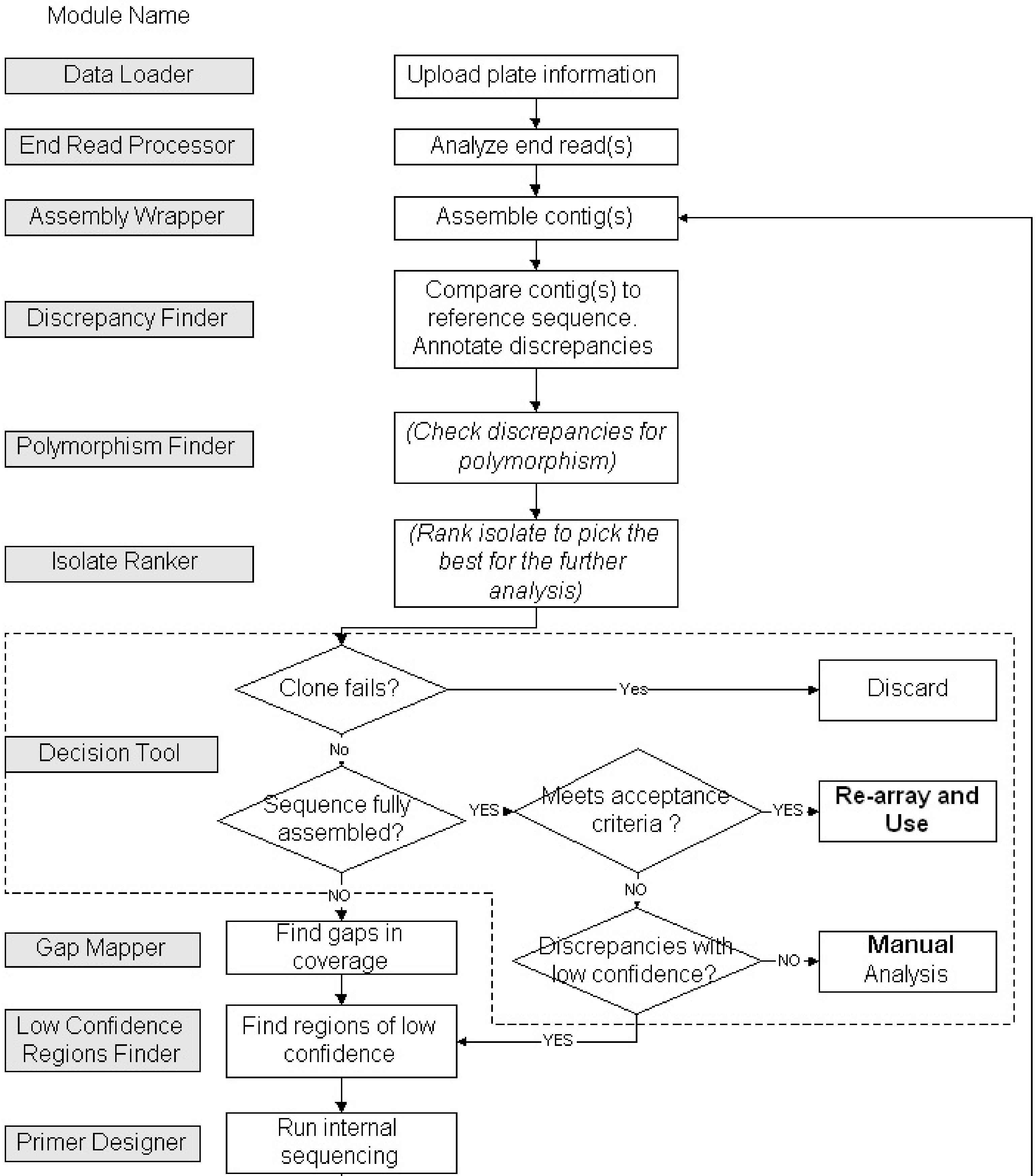

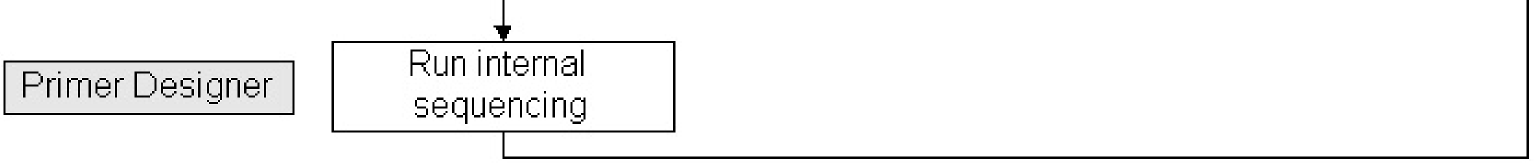
Primer Designer
Run internal sequencing

Translation of trace file name from sequencing facility name to ACE format

Sequence trace file names usually contain plate name, well and read direction in various configurations. The user describes the location of each of these items separately by specifying the separator, section number, offset inside each section, and the length. The separator is a string (often a single character such as the underscore in this example) that may be repeated in the name, breaking it into several 'sections'. The section number identifies the part that contains desired item; offset from the beginning of the section and length allow ACE to extract the item. If the sequencing facility applies its own name to the plate, it MUST provide a lookup table indicating the correspondence between the users' and the sequencing facility's plate names.

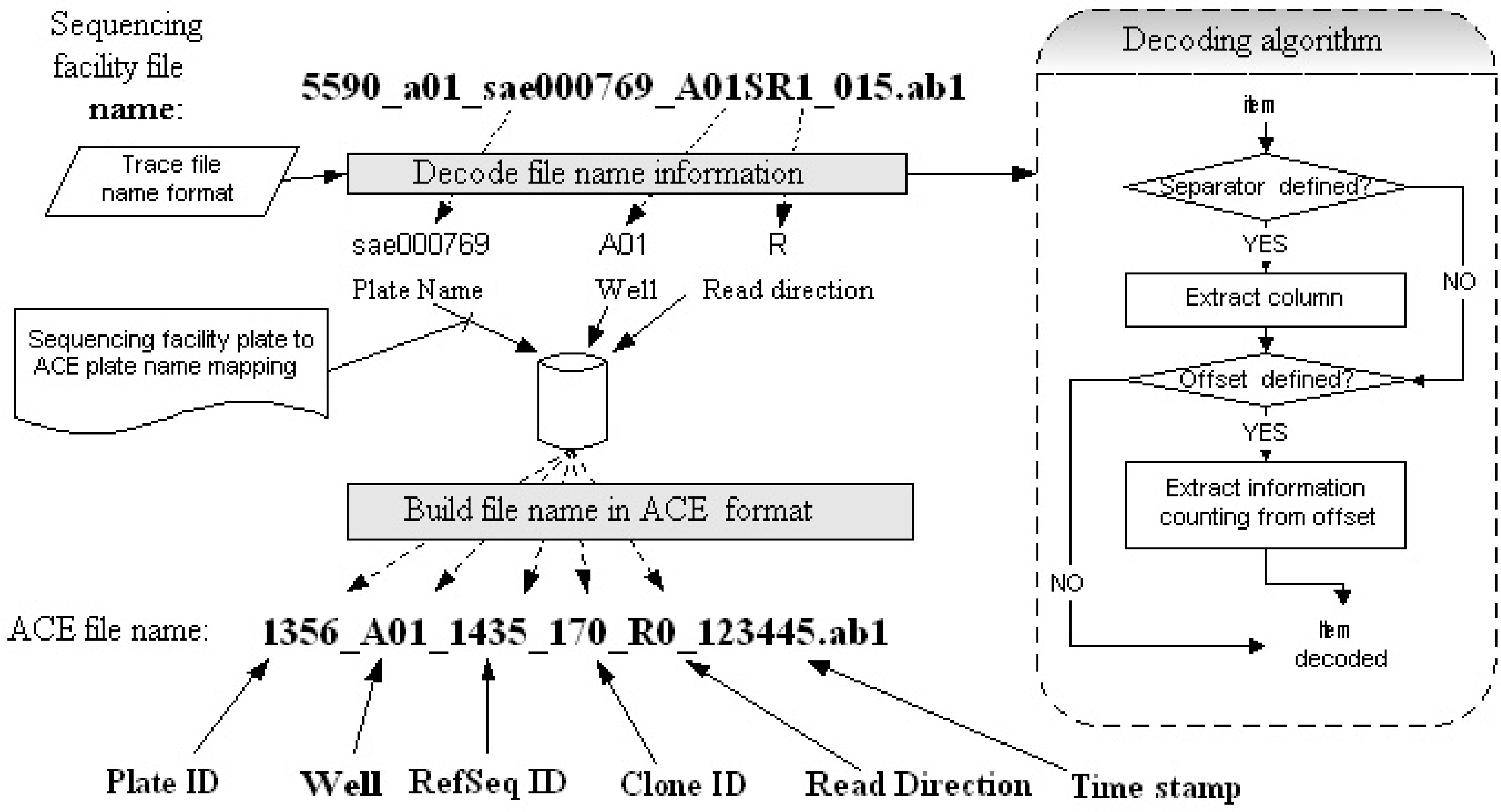

Trace File Distribution in ACE

Introduction

Clone validation projects often include sequence reads from different clones representing similar target sequences (e.g., paralogs, multiple isolates of the same gene, etc.). To prevent faulty assemblies, it is essential that reads from closely related clones do not end up in one another's analysis. The ACE package creates a hierarchical directory structure and stores all files related to a single clone in a directory specific to that clone, as required by the Phred/Phrap package.

The Trace File Distributor parses identifier information encoded in the filename for each trace file, that indicates which clone it belongs to. However, because different facilities utilize different naming conventions, the Trace File Distributor stores each facility's format and uses that stored information to convert the filename into a format that ACE can use to process these files automatically.

Trace file name format definition

To be recognizable by ACE a trace file name MUST include information about the plate label and position of each clone on the plate (Translation of trace file name from sequencing facility name to ACE format). For end reads only, information about the direction of the read (forward or reverse) MUST be included in the trace file name as well.
There are two ways to inform ACE how to extract this information.

Example 1: 64h11s2.scf, where '64' - equals plate name, H11 - position, s - direction of the read (here: forward)

Example 2: VcxXG002291-D02-F_010.ab1, where 'VcxXG002291' - equals plate name, D02 - well (second D02), F - read direction

First: User defines start position and length of each item. In example 1, the well description in the file name starts with position 3 and ends at position 5 (start 3, length 3), the direction is described by the single letter at positon 6 (start 6, length 1).

Second: User defines a separator that devides the file name into several columns and gives the number of each relevant column. Moreover, the user can define that in column *'N'* letters / digits from position *'a'* to position *'b'* describe the item. In example 2, the separator is '-' and the plate name would be column 1, the well column 2, and the direction column 3.

How to find what trace file name formats exist

From the drop down menu select : '*Trace Files > Create name format*'. All existing ACE trace file name formats will be listed in a table allowing the user to verify whether an appropriate trace name format already exists, and if not to create one.

How to create new format

The user will

- Select from drop down menu on the left: '*Trace Files > Create name format*'
- Enter 'example trace file name' with extension (all trace files processed by ACE must have an extension visible by OS)
- Give new format a name (format name must be unique. Consult table for existing format names)
- Define in what direction trace file name should be read by ACE (from 'left to right' or from 'right to left')
- Define how plate label information should be extracted
- Define how position information should be extracted

  For end reads only
- Define how direction of the read is annotated
- Define how direction information should be extracted

Upon pressing the 'Verify new format' button ACE will parse the example file name based on the new format description and will show on a new screen the plate label, well and read direction. If these items have been extracted properly select 'Submit', otherwise go back and change format definition(s).

Note: Do not change value of fields that are not involved in your format definition.

Examples

Format Name	File name reading direction	Plate Label Separator	Plate Label Column	Plate Label Start	Plate Label Length	Well Separator	Well Column	Well Start	Well Length	Direction Forward	Direction Reverse	Direction Separator	Direction Column	Direction Length	Direction Start	Example file name	Delete Format
HMBCT	Left to Right	[_]	2	1	2	[_]	2	3	3	s	r	[_]	2	1	6	Example	Delete
VXG-short	Left to Right	[_]	3	1	11	[-]	2	Not Set	Not Set	F	R	[-]	3	1	1	Example	Delete

Format *HMBCT*

File name: bk_64h11s2.scf , where '64' - equals plate name, H11 - position, s - direction of the read (forward)

Format ***VXG-short***

File name: 1119_D02_VcxXG002291-D02-F_010.ab1 , where 'VcxXG002291' - equals plate name, D02 - well (second D02), F - read direction

Definition of Discrepancy Types.

Discrepancy types for cDNA and flanking sequences defined by ACE on the nucleotide and polypeptide levels.

Discrepancies defined on nucleotide and polypeptide levels	
**Nucleotide changes**	**Polypeptide changes**
Substitution	Silent substitution
	Conservative substitution
	Non-Conservative substitution
	Missense substitution – any amino acid change
	Truncation – inframe Stop codon
Frameshift deletion	Frameshift deletion
Frameshift insertion	Frameshift insertion
Inframe deletion	Inframe deletion
Inframe insertion	Inframe insertion
No Stop codon	Post-elongation
No Start codon	No Translation
Discrepancies defined on nucleotide level only	
**Discrepancies introduced by sequence ambiguity**	
Start codon substitution	
Stop codon substitution	
Substitution in CDS region	
Frameshift insertion	
Inframe insertion	
**Discrepancies introduced by reference sequence ambiguity**	
Substitution in CDS region	
**Flanking sequence region (5’ and 3’ regions described separately)**	
Substitution – replacement of one base by another	
Deletion / insertion – deletion / insertion of several bases	
Ambiguous substitution - replacement of a base by ambiguous base	
Ambiguous insertion – insertion of ambiguous base	

Plate viewer and sample reports

A screenshot of the plate map with color-coded clone rank produced by the Isolate Ranker. Sample Report provides information about the available end reads, contig collections and fully assembled sequences for the clone; Discrepancy Report provides details about each discrepancy for the contig.

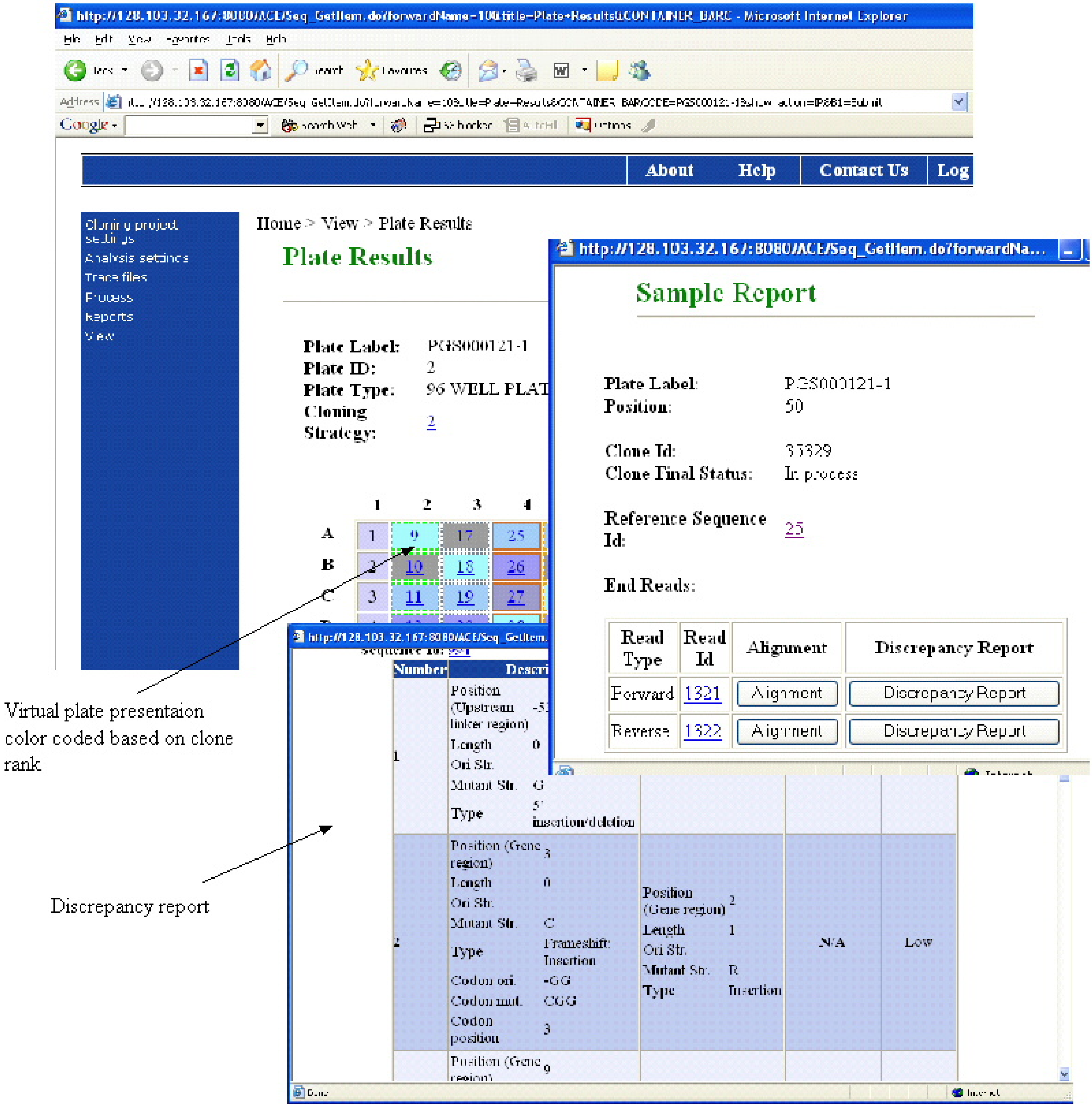

Block diagram of Decision Tool

This figure illustrates the logic used by the Decision Tool to assign clones to their various action groups indicated by the colored blocks. The tool will process any clones in the system. However, as the definition of clone quality is based upon comparing the list of discrepancies for the clone with the users' specifications for discrepancies, sorting clones to certain sub-groups requires a previous run through the Discrepancy Finder. Each action group is a separate tab-delimited list of clones that can be used to initiate the corresponding process.

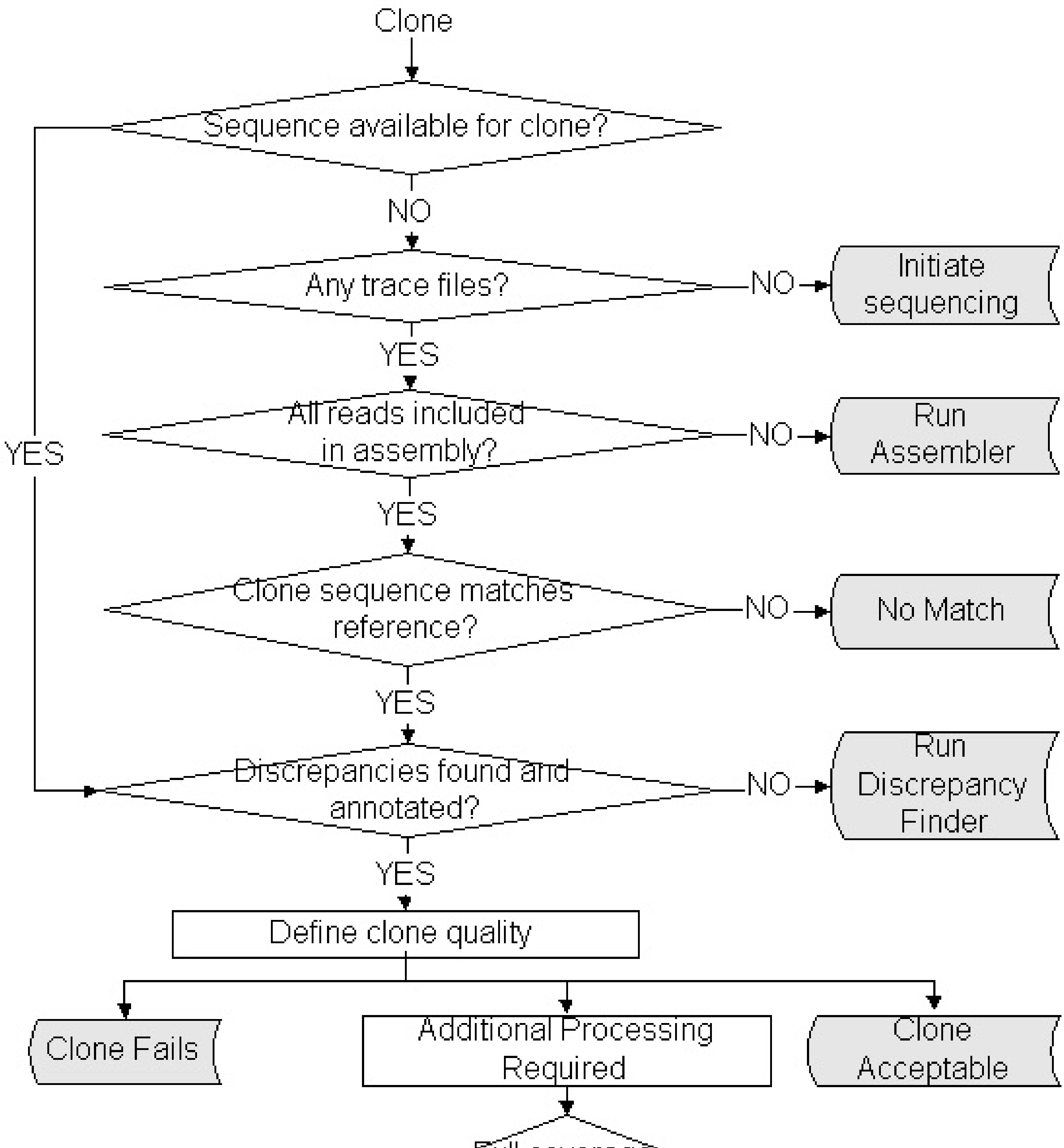

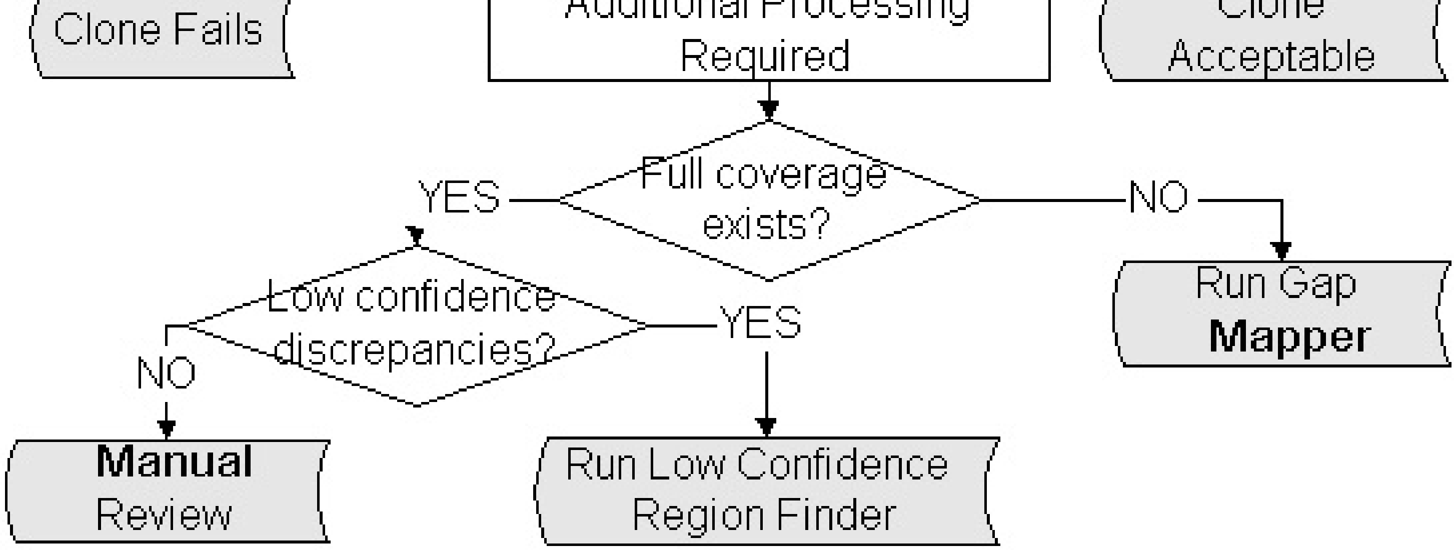

Clone Fails
Additional Processing Required
Clone Acceptable
Full coverage exists?
YES
NO
Run Gap Mapper
Low confidence discrepancies?
YES
NO
Manual Review
Run Low Confidence Region Finder

Gap Mapper contig calculation

Clone reads deemed to be of sufficient quality are supplemented by a pseudo read generated from the reference sequence with all bases set to a confidence score of 19 and submitted to the Phrap assembly process. The alignment matrix of clone reads is retrieved from the Phrap output in order to determine the positions of each read relative to the reference sequence. Consensus sequences and confidence scores are computed for the various contigs using a naïve Bayes classifier, and the results are reported. The calculated contigs are trimmed to drop low confidence tails.

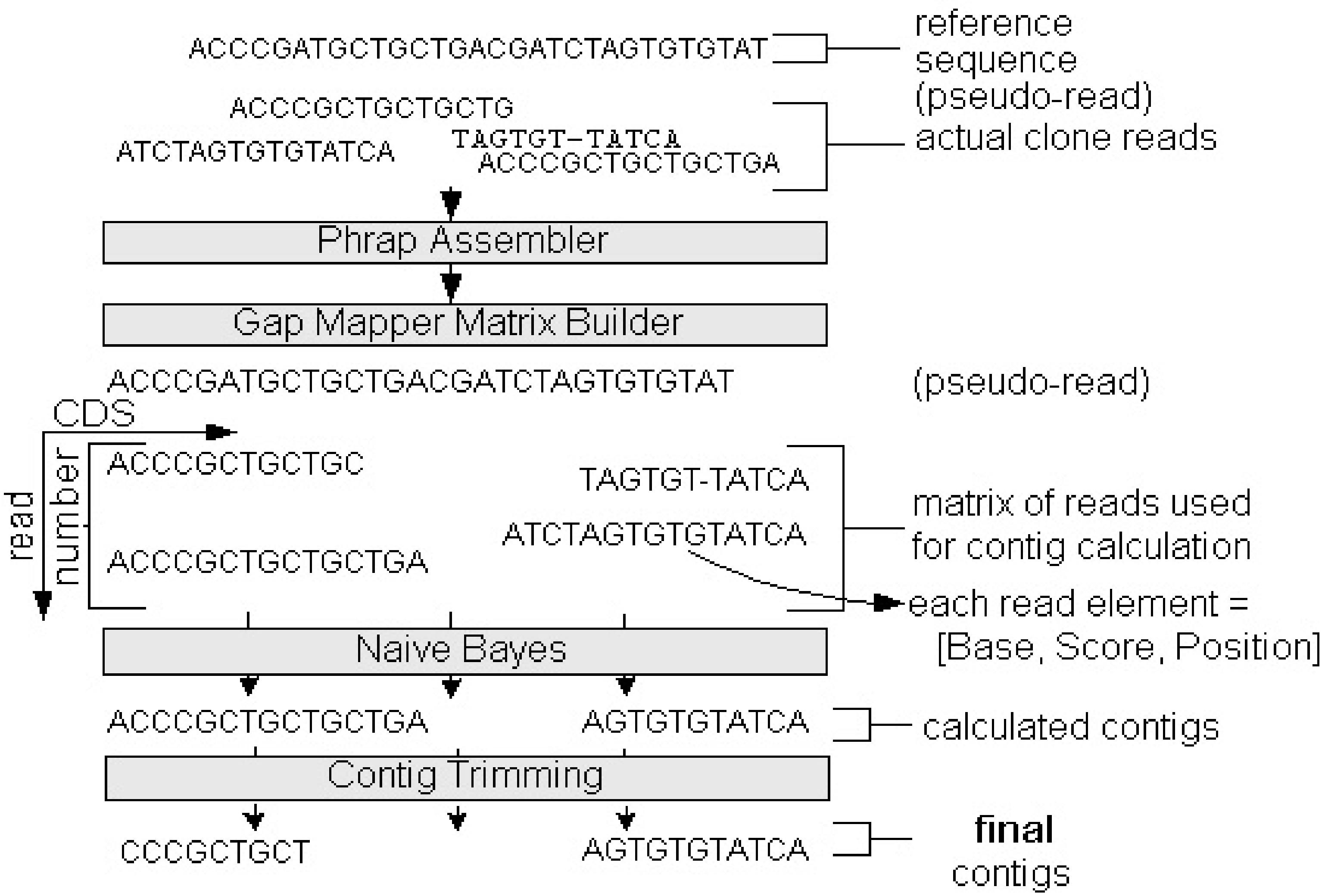

Parameter Settings for Sequencing Primer Design

For primer design ACE uses 'Primer3' - freely distributed software developed by the Whitehead Institute for Biomedical Research. 'Primer3' is specifically designed to pick primers for PCR reactions and has no built-in algorithm for primer walking. We developed a 'Primer3' wrapper that automates primer design for full-length cDNA sequencing of multiple clones. Specifications for Primer Designer include (1) sequence related parameters used by 'Primer3' (e.g., Tm, GC content - see parameter description below the entry form); (2) sequence processing parameters (e.g., length of reliable part of sequencing reads); (3) type of desired coverage (e.g., single forward, single reverse, double coverage). Sequence processing parameters define the quality of the expected reads and whether universal primers were used for end reads, and how many bases of ORF region these reads are expected to cover. **Note**: use Primer Designer specification with parameters '**Distance between 5p Universal Primer and START codon**' and '**Distance between 3p Universal Primer and STOP codon**' set to 0 when designing primers for stretch collections.

The main form to create a new Primer Design Specification is accessible through '**Analysis Settings > Primer Designer**'

Create New Set of Parameter for Primer Design

View Mine View All Delete

Set Name [ ] - unique specification name

Primer Design Parameters

**Primer Length (bp)**	**Min:** 18	**Optimal:** 21	**Max:** 27
**Primer Tm (C)**	**Min:** 57	**Optimal:** 60	**Max:** 63
**Primer GC%**	**Min:** 30	**Optimal:** 50	**Max:** 70

Sequencing Parameters

☐ **Check here if NO 5p Universal Primer is used (The most upstream forward PCR primer is used when No 5p Universal Primer is checked)**

☐ **Check here if NO 3p Universal Primer is used**

**Distance between 5p Universal Primer and START codon (For a left primer, primer start position is the position of the leftmost base)**	100	**bases**
**Distance between 3p Universal Primer and STOP codon (For a right primer, primer start position is the position of the rightmost base)**	100	**bases**
**Estimated high quality read length (ERL)**	400	**bases**
**Window size for testing primers**	50	**bases**
**Distance between sequencing primer and start of high quality read length**	50	**bases**
**Number of strands to sequence**	◉ **Single Strand (Coding strand, forward primers only)**<br>○ **Single Strand (Compliment to coding strand, reverse primers only)**<br>○ **Both Strands (Both forward and reverse primers until meet in middle)**<br>○ **Both Strands (Both forward and reverse primers, double coverage)**	

Polymorphism Finder Specification

Polymorphism Finder is working in two steps. First, a relatively short sequence segment comprising the discrepancy and flanking nucleotides is compared using NCBI BLAST to data from all user-selected GenBank databases to find a perfect match. The number of flanking nucleotides is defined by the '**Number of flanking bases to append on both sides of discrepancy for search**' parameter. However, it should be less or equal to 20. The list of available GenBank databases on a local non-ACE server is defined by the administrator in the ACE configuration file as set of paired parameters (PF_DB_1...*N* - PF_DB_1...*N*_NAME). The second step in the process is the verification of each 100% hit by comparing the entire clone target sequence with the hit sequence using Pairwise BLAST. The resulting alignment should satisfy the '**minimum match length**' and '**required percent identity**' requirements to confirm polymorphism.

The main form to create a new Polymorphism Finder Specification is accessible through '*Analysis Settings > Polymorphism Detection*'

Create New Set of Parameters for Polymorphism Detection

View Mine **View All**

Set Name [ ] - unique specification name

**Please select GenBank database to search in:**	High Throughput Genomic Sequences<br>month htgs
**Number of flanking bases to append on both sides of discrepancy for search:**	20
**Parameters to confirm similarity between the query and any database hits to confirm they are the same gene:**	
minimum match length:	100
required percent identity:	95

Sequence Trimming Specification

Contigs calculated by Gap Mapper and Low Confidence Region Finder are trimmed at the 5' and 3' ends excluding low quality sequences to ensure that complete coverage will be achieved with primers designed based on the contig information. The low quality base trimming procedure incorporates a sliding window algorithm to scan the sequence for low quality regions. The process requires the following parameters:

- **Phred base score defining high quality sequence** - high quality score cut-off for each base;
- **Sliding window size for low-quality trimming** - sliding window size, which specifies the number of DNA bases of the sliding window;
- **Max number of low-quality bases allowed** - Maximum number of low quality bases per window, above which a window is considered to be a low quality region and is trimmed;

Sequence Trimming Specification guides the treatment of closely located low confidence regions on clone contigs and short contigs surrounded by two gaps in Low Confidence Region Finder and Gap Mapper to make primer placement more efficient. For example, a short contig (length < '**Minimum contig length**') will be dropped, if two gaps surround the contig, and they will be collapsed into one long gap. This long gap will became a subject for primer coverage (see Figure below).

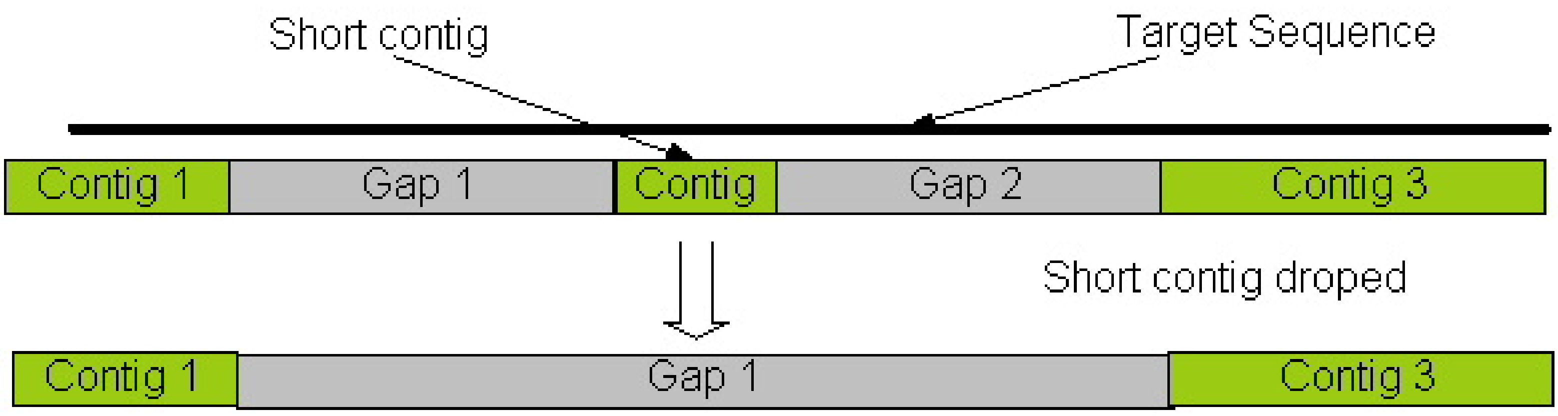

The same rule applies to two low confidence regions located close to each other: if the distance between them is less than defined in '**Minimum distance between sequence stretches**', they will be collapsed into one low confidence region and create a single subject for primer coverage.

The main form to create a new Sequence Trimming Specification is accessible through '*Analysis Settings > Sequence Trimming*'

Create New Set of Parameter for Sequence Trimming

View Mine View All

Set Name [ ] - unique specification name

**Phred base score defining high quality sequence:**	25

**Sliding window size for low-quality trimming:**	25 nt
**Max number of low-quality bases allowed:**	3
**Max number of consecutive low-quality bases allowed (not implemented):**	2
**Sliding window size for ambiguous base trimming (not implemented yet):**	25
**Max number of ambiguous bases allowed (not implemented yet):**	3
**Max number of consecutive ambiguous bases allowed (not implemented yet):**	2

**Minimum distance between sequence stretches needed to treat them separately (Gap Mapper, Low Confidence Finder):**	50
**Minimum contig length (used by GAP Mapper only) :**	50

**Sequence Trimming Type:**	Moving Window with extension for no

XML files for plate upload

---

1. Reference sequence file

The reference sequence file describs target sequences for clone(s). All steps on the '*Cloning project settings*' menu (see ACE tutorial) MUST be performed before the XML file can be written, because some items, like species, should be defined and get their unique ACE IDs before they can be referenced in the XML. Example of Reference sequence description XML file.

Elements

Sequence-info

**Element**	sequence-info				
**Description**	Root element, container for all reference sequences				
**Attributes**	**Name**	**Type**	**Optional/ Required**	**Default Value**	**Description**
	none				
**Contents**	refsequence*				

* - Zero or more ocurrences of an element

Refsequence

**Element**	refsequence
**Description**	represent one target (reference) sequence
**Contents**	refsequence-id, refsequence-species, refsequence-cds-start, refsequence-cds-stop, refsequence-cDNAsource, refsequence-chromosome, refsequence-sequence, refsequence-feature*

Refsequence-id

**Element**	refsequence-id
**Description**	user unique ID for target sequence, data type: integer

**Contents**	none

Refsequence-species

**Element**	refsequence-species
**Description**	unique species ID, Species definition MUST be submitted into ACE, see '*System Settings* ' for details
**Contents**	none

Refsequence-sequence

**Element**	refsequence-sequence
**Description**	reference sequence text downloaded from GenBank or other source
**Contents**	none

Refsequence-cds-start

**Element**	refsequence-cds-start
**Description**	CDS start on reference sequence text, 1 based
**Contents**	none

Refsequence-cds-stop

**Element**	refsequence-cds-start
**Description**	CDS stop on reference sequence text, 1 based
**Contents**	none

Refsequence-cDNAsource

**Element**	refsequence-cDNAsource
**Description**	reference sequence source
**Contents**	none

Refsequence-chromosom

**Element**	refsequence-chromosom
**Description**	chromosom of target sequence
**Contents**	none

Refsequence-feature

<table>
<tr><td>Element</td><td colspan="5">refsequence-feature</td></tr>
<tr><td>Description</td><td colspan="5">represent one annotation to reference sequence</td></tr>
<tr><td rowspan="5">Attributes</td><td>Name</td><td>Type</td><td>Optional/
Required</td><td>Default Value</td><td>Description</td></tr>
<tr><td>name_type</td><td>String</td><td>Required</td><td>n/a</td><td>Annotation type defined under 'Cloning Project Settings ' menu</td></tr>
<tr><td>name_value</td><td>String</td><td>Required</td><td>n/a</td><td>Annotation value, e.g., GI for target sequence</td></tr>
<tr><td>url</td><td>String</td><td>Optional</td><td>n/a</td><td>Link to annotation record, e.g., link to PubMed record for GI</td></tr>
<tr><td>description</td><td>String</td><td>Optional</td><td>n/a</td><td>Annotation description</td></tr>
<tr><td>Contents</td><td colspan="5">none</td></tr>
</table>

2. Plate mapping file

The XML file describ the plate mapping information and cloning strategy. All steps on the 'Cloning project settings' menu (see ACE tutorial) MUST be performed before the XML file can be written, because some items, like cloning strategy, should be defined and have their unique ACE IDs before they can be referenced in the XML file. Example of Plate mapping XML file.

Elements

Clone collections

**Element**	clone-collections				
**Description**	Root element, definition of sequencing plate collection				
**Attributes**	**Name**	**Type**	**Optional/ Required**	**Default Value**	**Description**
	none				
**Contents**	clone-collection*				

Clone collection

**Element**	clone-collection				
**Description**	Definition of sequencing plate				
**Attributes**	**Name**	**Type**	**Optional/ Required**	**Default Value**	**Description**
	userid	int	Required	*n/a*	User unique ID, user must be register with ACE
	name	String	Required	*n/a*	Unique plate name
	type	String	Optinal	*n/a*	Container type description
	projectid	int	Required	*n/a*	Project unique ID, see '*System Settings*' for details
**Contents**	sample, construct				

Construct

**Element**	construct				
**Description**	container for all isolate originated from the same clone				
	**Name**	**Type**	**Optional/ Required**	**Default Value**	**Description**

**Attributes**	format	enum	Required	*1*	Defins whether stop codon was replace by non-stop codon or not
	cloningstrategy_name	int	Required	*n/a*	Cloning strategy ID, see '*System Settings*' for details
	refsequenceid	int	Required	*n/a*	Target sequence ID as submited from reference
	constructid	int	Required	*n/a*	Construct unique identifier
**Contents**	sample*				

Construct format:

- 0 - stop codon was replaced by non-stop codon;
- 1 - stop codon preserved;

Sample

**Element**	sample				
**Description**	represent one phisical sample on sequencing plate, clone ID is substance identifier.				
	**Name**	**Type**	**Optional/ Required**	**Default Value**	**Description**

**Attributes**	sampleid	int	Required	*n/a*	Unique sample ID; can be equal to clone ID depending on customer LIMS.
	cloneid	int	Required	*n/a*	Unique clone ID
	well	String	Required	*n/a*	Well name in 'A01' annotation
	sampletype	enum**	Required	*n/a*	Sample type: see below for possible values
**Contents**	empty				

Sample Type:

- CONTROL_POSITIVE
- CONTROL_NEGATIVE
- ISOLATE
- EMPTY

  - empty well

Note: All attributes of any type (int, enum,..) MUST be placed in quots in the XML file, e.g., <construct constructid='27433' format= '0' cloningstrategyid='5' ....

```
<!-- Copyright 2005, 2006 President and Fellows of Harvard College. All Rights
Reserved.  -->
<?xml version="1.0" encoding="ISO-8859-1" ?>
<!DOCTYPE web-app (View Source for full doctype...)>
<!--  XML file with reference sequence descriptions.   -->
- <sequence-info>
  - <refsequence>
      <refsequence-id>id</refsequence-id>
      <refsequence-species>speciesid</refsequence-species>
      <refsequence-cds-start>1</refsequence-cds-start>
      <refsequence-cds-stop>1000</refsequence-cds-stop>
      <refsequence-cDNAsource />
      <refsequence-chromosome>Xq12-q13</refsequence-chromosome>
      <refsequence-
        sequence>AATGGAGTGGATGCGCTATCAAATCTACTACATAGGACAACACACACACCGAAC[illegible]
        -sequence>
      <refsequence-feature name_type="GENBANK_ACCESSION"
        name_value="NM_004299" url="" description="ATP-binding cassette, sub-
        family B (MDR/TAP), member 7" />
      <refsequence-feature name_type="GENE_SYMBOL" name_value="ABCB7" url=""
        description="" />
    </refsequence>
  - <refsequence>
      <refsequence-id>4476</refsequence-id>
      <refsequence-species>2</refsequence-species>
      <refsequence-cds-start>1</refsequence-cds-start>
      <refsequence-cds-stop>2019</refsequence-cds-stop>
      <refsequence-chromosome>14</refsequence-chromosome>
      <refsequence-
        sequence>ATGAAGCAGGAGCAGTCCCACGAAGGCGACTCATACAGCACGGAATTCATAAAT[illegible]
        -sequence>
    - <refsequence-feature>
        <name_type>GENBANK_ACCESSION</name_type>
        <name_value>CAA95979</name_value>
      </refsequence-feature>
    - <refsequence-feature>
        <name_type>SGD</name_type>
        <name_value>YNL103W</name_value>
        <description>member of the leucine zipper family of transcriptional
          activators</description>
      </refsequence-feature>
    - <refsequence-feature>
        <name_type>GI</name_type>
        <name_value>1302018</name_value>
      </refsequence-feature>
    - <refsequence-feature>
        <name_type>GENE_SYMBOL</name_type>
        <name_value>met4</name_value>
      </refsequence-feature>
    - <refsequence-feature>
        <name_type>ORF_TYPE</name_type>
        <name_value>Verified</name_value>
```

</refsequence-feature>
- <refsequence-feature>
<name_type>**ORF_STATUS**</name_type>
<name_value>**No change**</name_value>
</refsequence-feature>
</refsequence>
</sequence-info>

```
<?xml version="1.0" encoding="ISO-8859-1" ?>
<!DOCTYPE web-app (View Source for full doctype...)>
<!--  XML file with clone mapping information.   -->
<!--  XML file contains clone mapping data for the plate 'YGS000374-1'. The
plate is located at http://bighead:8080/ACE_DEMO/   -->
- <clone-collections>
  - <clone-collection userid="1" name="YGS000374-1" project_id="3">
    - <construct constructid="32316" format="1" cloningstrategyid="2"
        refsequenceid="4063">
        <sample sampleid="549322" cloneid="5272" well="A02"
          sampletype="ISOLATE" />
        <sample sampleid="549323" cloneid="5273" well="B02"
          sampletype="ISOLATE" />
        <sample sampleid="549324" cloneid="5274" well="C02"
          sampletype="ISOLATE" />
        <sample sampleid="549325" cloneid="5275" well="D02"
          sampletype="ISOLATE" />
      </construct>
    - <construct constructid="32441" format="1" cloningstrategyid="2"
        refsequenceid="4202">
        <sample sampleid="549354" cloneid="5408" well="E02"
          sampletype="ISOLATE" />
        <sample sampleid="549355" cloneid="5409" well="F02"
          sampletype="ISOLATE" />
        <sample sampleid="549356" cloneid="5410" well="G02"
          sampletype="ISOLATE" />
        <sample sampleid="549357" cloneid="5411" well="H02"
          sampletype="ISOLATE" />
      </construct>
    - <construct constructid="32488" format="1" cloningstrategyid="2"
        refsequenceid="4254">
        <sample sampleid="549358" cloneid="5452" well="A03"
          sampletype="ISOLATE" />
        <sample sampleid="549359" cloneid="5453" well="B03"
          sampletype="ISOLATE" />
        <sample sampleid="549360" cloneid="5454" well="C03"
          sampletype="ISOLATE" />
        <sample sampleid="549361" cloneid="5455" well="D03"
          sampletype="ISOLATE" />
      </construct>
    - <construct constructid="32528" format="1" cloningstrategyid="2"
        refsequenceid="4298">
        <sample sampleid="549374" cloneid="5504" well="E03"
          sampletype="ISOLATE" />
        <sample sampleid="549375" cloneid="5505" well="F03"
          sampletype="ISOLATE" />
        <sample sampleid="549376" cloneid="5506" well="G03"
          sampletype="EMPTY" />
        <sample sampleid="549377" cloneid="5507" well="H03"
          sampletype="ISOLATE" />
      </construct>
      <sample sampleid="549306" cloneid="0" well="A01"
        sampletype="CONTROL_POSITIVE" />
```

```
    <sample sampleid="549307" cloneid="0" well="B01"
      sampletype="CONTROL_POSITIVE" />
    <sample sampleid="549308" cloneid="0" well="C01"
      sampletype="CONTROL_POSITIVE" />
    <sample sampleid="549309" cloneid="0" well="D01"
      sampletype="CONTROL_POSITIVE" />
  </clone-collection>
</clone-collections>
```

Information for General Report

Tool goal:	
General Report is a tool designed to assist the researcher in accessing information about clones in ACE. The output, tab delimited file is send by e-mail to the user. There is no limit on number of submitted items.	
**Information provided by report**	
Field Name	***Description***
**Information type: Clone Information:**	
Clone ID	Clone ID is a unique clone identifier
Clone final status	Possible values: 'In Process', 'Accepted', 'Rejected'. If clone final status is set by user to 'Accepted' or 'Rejected', clone data cannot be modified
Sample ID	ACE sample ID
Plate label	Plate label
Sample type	Sample Type (Isolate, Empty, Control)
Sample well	Position on plate (1 - 96)
Clone score	Clone penalty score, calculated
Clone rank	Clone Rank, defines whether a clone (a) matches expected ORF; (b) is acceptable based on user criteria; (c) how the clone quality compares with other clones originating from the same isolate
**Information type: Clone raw data information**	
Directory name	Name of clone trace file directory
End read length (high quality region)	Length of high confidence region for forward and reverse end read
Status of clone sequence assembly	Status shows: (a) whether clone went throw assembly process; (b) assembly succeeded or not; (c) reason for assembly failure

**Information type: Reference Sequence Information**	
Sequence ID	ACE/User defined sequence ID, both values specified
CDS start	CDS start
CDS stop	CDS stop
CDS length	CDS length
GC Content	GC content of gene sequence
Sequence Text	Gene sequence (text) as was defined on reference sequence upload
CDS	Gene CDS
GI Number	GI for reference sequence
Gene Symbol	Gene symbol for reference sequence
Species specific ID	Species specific identifier as defined by user
All available identifiers	All identifiers for reference sequence, except for species specific identifier
**Infomation type: Clone Sequence Information**	
Clone sequence ID	Clone sequence ID, if clone is not fully assembled sequence ID equals to 0
Status of clone sequence analysis	Available values: 'Analyzed, discrepancies found' or 'Analyzed, no discrepancies found' - sequence was analyzed by Discrepancy Finder; 'Obtained' - sequence was submitted, but not analyzed
Clone sequence CDS start	Clone sequence CDS start
Clone sequence CDS stop	Clone sequence CDS stop
Clone sequence	Clone sequence text
High quality discrepancies (separated by type)	Summary for all high confidence discrepancies identified in assembled sequence. If sequence was submitted without Phred quality scores all discrepancies are assigned an 'UN KNOWN' confidence status and treated as high confidence discrepancies

Low quality discrepancies (separated by type)	Summary for all low confidence discrepancies identified in assembled sequence

Discrepancy Report

Sequence Id: 129992

Number	Description	Protein Description	Polymorphism	Confidence
1	Position (Gene region) 84<br>Length 1<br>Position (ExpSequence) 174<br>Ori Str. T<br>Mutant Str. C<br>Type Silent<br>Codon ori. TTT<br>Codon mut. TTC<br>Codon position 3	Position (Gene region) 28<br>Length 1<br>Ori Str. F<br>Mutant Str. F<br>Type Silent	**Y**<br>Details	High
2	Position (Gene region) 1313<br>Length 1<br>Position (ExpSequence) 1403<br>Ori Str. T<br>Mutant Str. C<br>Type Missense<br>Codon ori. GTT<br>Codon mut. GCT<br>Codon position 2	Position (Gene region) 438<br>Length 1<br>Ori Str. V<br>Mutant Str. A<br>Type Conservative Substitution	**Y**<br>Details	High

Polymorphism report for discrepancy

Polymorphism hits.

Clone Sequence Id: 129992
Discrepancy Id: 307298
GI Hits:

55625785
62087431
50345287
66898650
68283146
68223217
66256539
52289403
52119799

Gap Mapper Report

Clone ID	Ref Sequence ID	CDS Length	Region Type	CDS Start	CDS Stop	Sequence ID
134915	42988	1319	Contig	-49	34	206907
134915	42988	1319	LQR	17	34	206907
134915	42988	1319	Gap	35	549	N/A
134915	42988	1319	Contig	550	-1000	206908
134915	42988	1319	LQR	550	600	206908

Detailed Low Quality Finder Report

Clone ID	Ref Sequence ID	CDS Length	Clone Sequence ID	Sequence Analysis Status	Discrepancy ID	Discrepancy Type	Quality	CDS start	Discrepancy Length	Forward Read Needed	Internal Reads Needed	Reverse Read Needed
119565	42808	1412	55117	ADY	125952	Silent	Low	687	1	No	Yes	No
119612	42773	1505	55108	ADY	125768	Frameshift: Insertion	Low	726	0	No	Yes	No
119612	42773	1505	55108	ADY	125770	Amb. substitution	Low	865	1	No	Yes	No

Sequence Analysis Status: ADY - Analyzed Discrepancy Yes

Primer Designer Report

Clone ID 119721
Ref Sequence Length 1799
Ref Sequence ID 40842

Primer3 Spec ID 23 Name Position Sequence Tm Direction
F1 225 CTGGCTCCGGAATTCACT 58.7 Forward
F2 490 ACCAAAGGGAAGAAGCAGTT 57.9 Forward
F3 806 CTGTGTTGATGCACTACCCC 59.0 Forward
F4 1095 GTTCGGTGTCCAGTTCCAATAC 60.7 Forward
F5 1387 AATGAGTACCGCAAGAGGTTT 57.9 Forward

Primer3 Spec ID 36 Name Position Sequence Tm Direction
F1 10 GAGTCTCTTGCTCCGGTTC 57.5 Forward
F2 304 TGTCAATGCCACCTTCATC 57.9 Forward
F3 611 TTCACCCACCAGTTCTTCA 57.5 Forward
F4 910 CTGGCTACGTGAGCACAAC 58.0 Forward
F5 1235 ATGTTGGTGGACTATGGGG 58.1 Forward
R1 1477 ATTCCTCCAACTCTGCTGC 58.0 Reverse
R2 1190 CCCACCTTGAAGGAGTCAG 58.2 Reverse
R3 900 ATACAGCATGAGCCCAGGA 59.2 Reverse
R4 590 AACATGAGGTTGGTGCCTT 58.0 Reverse

Primer Order (example file 1)

Plate Name	Column	Row	Sequence ID	Primer Sequence
OPLATE000201	01	A	.	empty
OPLATE000201	01	B	748	GCAGCGACAAGAGGAAAAT
OPLATE000201	01	C	748	CGACTCACCAGACCTACCC
OPLATE000201	01	D	748	GGCAGTGATGATTGCCTTT
OPLATE000201	01	E	755	ACCAGACCAAAGTGGATGG
OPLATE000201	01	F	755	TTTGCTCAATTCCTCCTTTG
OPLATE000201	01	G	755	GTGGAGCCTTTTCTTCCTG
OPLATE000201	01	H	755	GAGGAGAGGGGGTGAAGTT
OPLATE000201	02	A	755	AAACAAGGTATCGCCAGAGA
OPLATE000201	02	B	755	GCAGTGGCCTGAACAAAG
OPLATE000201	02	C	759	GAAAAGGCTGGCAGAGAAG
OPLATE000201	02	D	759	CCTCTTTGTTCAGGCCACT
OPLATE000201	02	E	615	TGCAAAGATGACAGTGTGCT
OPLATE000201	02	F	615	TCAAGGGGGATCAGTTTGT
OPLATE000201	02	G	615	GGGACACATTCTCAGGGAC
OPLATE000201	02	H	638	AAGTGTGAGCTCCTCTCGG
OPLATE000201	03	A	638	TCAGCTGACAGCCAGAGAG
OPLATE000201	03	B	645	GAGGACGAGGAGGAAGAGA
OPLATE000201	03	C	.	empty

Primer Order (example file 2)

Row	Column	Sequence ID	Primer Sequence	Plate Name
A	01	182451	TCAAACGTGAAGATCGCC	OPLATE000927
B	01	182452	GCATGACGATGGTGGTTT	OPLATE000927
C	01	182453	CACCACGCCAGACATTAAA	OPLATE000927
D	01	182454	GCAAAACGGTCATTTGGA	OPLATE000927
E	01	182455	AAACGAATTGGCGATGAA	OPLATE000927
F	01	182456	GCAAACAGTTGGGTACGG	OPLATE000927
G	01	182457	ATTTTTGTCGGTGTGCGT	OPLATE000927
H	01	182458	GCGCACTGCTCAAATTCT	OPLATE000927
A	02	182459	ACCGCAATGGTGTTGAAT	OPLATE000927
B	02	182460	CCATCAGCATTCCTGGAG	OPLATE000927
C	02	182461	ACAAGTAGGACAGGCCGAG	OPLATE000927
D	02	182462	CAAATTCCACTTCGCCTTC	OPLATE000927
E	02	182463	CAACGGCAATCTTGTGAGA	OPLATE000927
F	02	182464	TTGGCCCTTCATAACGAAT	OPLATE000927
G	02	182465	ACCGCAATGGTGTTGAAT	OPLATE000927
H	02	182466	CCATCAGCATTCCTGGAG	OPLATE000927
A	03	182467	ACAAGTAGGACAGGCCGAG	OPLATE000927
B	03	182468	CAAATTCCACTTCGCCTTC	OPLATE000927
C	03	182469	CAACGGCAATCTTGTGAGA	OPLATE000927
D	03	182470	TTGGCCCTTCATAACGAAT	OPLATE000927
E	03	182471	GGCTCTTGAGCCCAGTTT	OPLATE000927
F	03	182472	CACCGATTTGCTGGACAT	OPLATE000927
G	03	182473	GCACAACAAAGATGGCCT	OPLATE000927
H	03	182474	CCCAATGCTGACAAGGAT	OPLATE000927
A	04	182475	GGCTCTTGAGCCCAGTTT	OPLATE000927
B	04	182476	CACCGATTTGCTGGACAT	OPLATE000927
C	04	182477	GCACAACAAAGATGGCCT	OPLATE000927
D	04		***** OPLATE000927	
E	04		***** OPLATE000927	

Screenshot of 'View Internal Primers' report for clone

Home > Process > View Results > View Internal Primers

View Internal Primers

Clone Id:	159188
Primer3 Specification:	45
Reference Sequence Id:	42864

Name	Sequence	Tm	Position	Orientation	Status	Submission Type	Submitter
F1	TGAAACCACTGAAAGGGG	56.939	167	Forward	Designed	Gene specific, calculated	System
F2	TCCAAATGTTGATGCTAGATTG	57.781	315	Forward	Designed	Gene specific, calculated	System
F3	TGACAGAAAAAGTAGGCTTGG	57.175	482	Forward	Designed	Gene specific, calculated	System
F4	GGTTTCAAATGTGTGACAGGA	58.456	610	Forward	Designed	Gene specific, calculated	System
F5	GCAATGCAAAACTCTGGC	57.905	769	Forward	Designed	Gene specific, calculated	System
F6	AGCCCACCATGTGTTCAG	57.934	918	Forward	Designed	Gene specific, calculated	System

Primer3 Specification:	40
Reference Sequence Id:	42864

Name	Sequence	Tm	Position	Orientation	Status	Submission Type	Submitter
F1	CCCGATTACACCTCAACAAG	58.077	98	Forward	Designed	Gene specific, calculated	System
F2	CCAAATGTTGATGCTAGATTGT	56.81	316	Forward	Designed	Gene specific, calculated	System
F3	TAGCAGACAGACAGGGCTTT	57.7	521	Forward	Designed	Gene specific, calculated	System
R3	TCTTCGGCAATGTACTGAGC	59.028	707	Reverse	Designed	Gene specific, calculated	System
R2	AATTAAACTGGCAACAAAGCA	57.528	906	Reverse	Designed	Gene specific, calculated	System
R1	GGTCCCATAATGCAGACAAA	58.423	1111	Reverse	Designed	Gene specific, calculated	System

Primer3 Specification: 48
Reference Sequence Id: 42864

Name	Sequence	Tm	Position	Orientation	Status	Submission Type	Submitter
R3	TCATTTGATTCACCATGTCAG	56.935	484	Reverse	Designed	Gene specific, calculated	System
R2	CCTGCTTGATCCAACACA	56.452	671	Reverse	Designed	Gene specific, calculated	System
R1	TTCTGAAATTGGCTCACAAA	56.91	885	Reverse	Designed	Gene specific, calculated	System

Screenshot of 'Oligo Order' report for clone

Home > Process > View Results > View Oligo Order(s) for Clone(s)

Oligos Ordered for Clone

Clone ID	Plate	Well	Plate Status	Plate Order Date	Oligo ID	Oligo Name	Oligo Sequence	Oligo Position
159196	OPLATE000820	71	Used for sequencing	2005-06-23	S148361	F1	CGCCAGGTGTTGATCTACTT	437
	OPLATE000820	72	Used for sequencing	2005-06-23	S148362	F2	ACCTCAACCGAAGCTTTCC	645
	OPLATE000820	73	Used for sequencing	2005-06-23	S148363	F3	AACATAAGGCCACTGGAATCT	816
	OPLATE000820	74	Used for sequencing	2005-06-23	S148364	F4	TATGTGTGGGCCAACTGTTT	1016
	OPLATE000820	75	Used for sequencing	2005-06-23	S148365	F5	ATCACAACAGGCAGATTTGG	1232

Screenshot of Oligo Plate report

Home > Process > View Process Results > View Oligo Plate

View Oligo Plate

Plate label: OPLATE000820
Plate ID: 1654
Order date: 2005-06-23
Plate status: Used for sequencing
Order comments: arrived 08/26/05. Project internal sequencing oligomers
Sequencing comments: shipped to ... for DNA plates .. Used Dec 14, 2005

Well	Clone ID	Oligo Name	Oligo Sequence	Oligo Position on Sequence	Oligo Tm
1	140264	R10	TTCAGATTCCAATTCTGCACT	918	57.406
2	140264	R9	TAAAGGTTGTGGTCTCTCCC	1149	56.671
3	140264	R8	CATTGAAGCCGAATAGAGGT	1341	56.909
4	140264	R7	CAAAGTACCTTACACCTTCACG	1543	56.559
5	140264	R6	GCCATAATCTGTTGGAAGGA	1757	57.61
6	159133	F1	AAGAAGATGGTGCTTTGGAA	1496	57.393
7	159133	F2	TTTTGGAGTCATTCTGGGAA	1693	58.117
8	159133	F3	TTCCCAGCCAGTAAAACAAG	1898	57.879

Example of Gap Mapper Results

Home > Process > View Results > View Latest Contig Collection

View Latest Contig Collection

Clone ID:	**120016**
**Reference Sequence ID:**	42786

Contig Name	Contig Type	Contig ID	CDS Start	CDS Stop	LQR defined	Alignment	Discrepancy Report
Gap 1	Gap	15467	-1000	844			
Contig 1	Contig	15465	845	1154	Yes	Alignment	Discrepancy Report
Gap 2	Gap	15468	1155	2462			
Contig 2	Contig	15466	2463	3295	Yes	Alignment	No discrepancies
Gap 3	Gap	15469	3296	-1000			

Screenshot of 'Low Confidence Regions' report for clone sequence

View Low Confidence Regions for clone sequences

Home > Process > View Results > View Low Confidence Regions for Clone Sequences

Clone Id:	139940
**Reference sequence ID:**	42840
**Clone sequence ID:**	55127
**Clone sequence analysis status:**	
**Clone sequence aligment:**	Alignment
**Specification for LQR definition:**	32

Name	CDS Region	Sequence Region	Sequence	Discrepancy Report
LQR 1	513 - 745	562 - 794	GTGGGACCGCTCCTACTTCGCGCAGAGGCCAGGGGGTAAA<br>GAGGCGCTACCAGGCGGGCGGGCCCCGGCGCTTCTGTACC<br>GCCTGCTGCTTTTGCGGTGAAGACCACCCGCAGCAGGGCA<br>GCACCCTTTACTGCGTGCCCACGAGCACAAATCAAGCGCA<br>GGCGGCTCCGGAGGAGCGGCCGAGGGCCCCCTGGTGGGAC<br>ACCTCCTCTGGTGCGCTGCGGCCGGTGGCGCTC	Discrepancy Report
